# Fat-Secreted Ceramides Regulate Vascular Redox State and Influence Outcomes in Patients With Cardiovascular Disease

**DOI:** 10.1016/j.jacc.2021.03.314

**Published:** 2021-05-25

**Authors:** Nadia Akawi, Antonio Checa, Alexios S. Antonopoulos, Ioannis Akoumianakis, Evangelia Daskalaki, Christos P. Kotanidis, Hidekazu Kondo, Kirsten Lee, Dilan Yesilyurt, Ileana Badi, Murray Polkinghorne, Naveed Akbar, Julie Lundgren, Surawee Chuaiphichai, Robin Choudhury, Stefan Neubauer, Keith M. Channon, Signe S. Torekov, Craig E. Wheelock, Charalambos Antoniades

**Affiliations:** a Division of Cardiovascular Medicine, Radcliffe Department of Medicine, University of Oxford, Oxford, United Kingdom; b Department of Genetics and Genomics, College of Medicine and Health Sciences, United Arab Emirates University, Al-Ain, United Arab Emirates; c Division of Physiological Chemistry II, Department of Medical Biochemistry and Biophysics, Karolinska Institute, Stockholm, Sweden; d Novo Nordisk Foundation Center for Basic Metabolic Research, Faculty of Health and Medical Sciences, University of Copenhagen, Copenhagen, Denmark; e Department of Biomedical Sciences, Faculty of Health and Medical Sciences, University of Copenhagen, Copenhagen, Denmark

**Keywords:** adipose tissue, C16:0-ceramide, cardiovascular disease, metabolomics, sphingolipids, vascular redox state, AT, adipose tissue, Cer16:0, C16:0-ceramide, eNOS, endothelial nitric oxide synthase, GlcCer16:0, C16:0-glycosylceramide, GLP-1, glucagon-like peptide-1, HAEC, human aortic endothelial cell, L-NAME, N^G^-nitro-l-arginine methyl ester, NADPH, reduced nicotinamide adenine dinucleotide phosphate, PP2A, protein phosphatase 2, ScAT, subcutaneous adipose tissue, SPL, sphingolipid, ThAT, thoracic adipose tissue

## Abstract

**Background:**

Obesity is associated with increased cardiovascular risk; however, the potential role of dysregulations in the adipose tissue (AT) metabolome is unknown.

**Objectives:**

The aim of this study was to explore the role of dysregulation in the AT metabolome on vascular redox signaling and cardiovascular outcomes.

**Methods:**

A screen was conducted for metabolites differentially secreted by thoracic AT (ThAT) and subcutaneous AT in obese patients with atherosclerosis (n = 48), and these metabolites were then linked with dysregulated vascular redox signaling in 633 patients undergoing coronary bypass surgery. The underlying mechanisms were explored in human aortic endothelial cells, and their clinical value was tested against hard clinical endpoints.

**Results:**

Because ThAT volume was associated significantly with arterial oxidative stress, there were significant differences in sphingolipid secretion between ThAT and subcutaneous AT, with C16:0-ceramide and derivatives being the most abundant species released within adipocyte-derived extracellular vesicles. High ThAT sphingolipid secretion was significantly associated with reduced endothelial nitric oxide bioavailability and increased superoxide generated in human vessels. Circulating C16:0-ceramide correlated positively with ThAT ceramides, dysregulated vascular redox signaling, and increased systemic inflammation in 633 patients with atherosclerosis. Exogenous C16:0-ceramide directly increased superoxide via tetrahydrobiopterin-mediated endothelial nitric oxide synthase uncoupling and dysregulated protein phosphatase 2 in human aortic endothelial cells. High plasma C16:0-ceramide and its glycosylated derivative were independently related with increased risk for cardiac mortality (adjusted hazard ratios: 1.394; 95% confidence interval: 1.030 to 1.886; p = 0.031 for C16:0-ceramide and 1.595; 95% confidence interval: 1.042 to 2.442; p = 0.032 for C16:0-glycosylceramide per 1 SD). In a randomized controlled clinical trial, 1-year treatment of obese patients with the glucagon-like peptide-1 analog liraglutide suppressed plasma C16:0-ceramide and C16:0-glycosylceramide changes compared with control subjects.

**Conclusions:**

These results demonstrate for the first time in humans that AT-derived ceramides are modifiable regulators of vascular redox state in obesity, with a direct impact on cardiac mortality in advanced atherosclerosis. (The Interaction Between Appetite Hormones; NCT02094183)

Cardiovascular disease remains the number one killer worldwide, and this is exacerbated by the global obesity pandemic. There is an unmet need to identify the pathophysiological mechanisms linking obesity with cardiovascular disease to develop therapeutic interventions preventing cardiovascular complications of obesity.

Studies of human arteries from patients undergoing cardiac surgery have provided useful insights into the mechanisms of vascular disease pathogenesis in the context of obesity (1). Prior studies have demonstrated that adipose tissue (AT) secretes a wide range of molecules with endocrine and paracrine effects on the cardiovascular system, including redox signaling and other key pathogenic mechanisms of vascular disease (1,2). These findings suggest that the human AT secretome includes multiple, largely unknown bioactive molecules that could serve as mechanistic biomarkers and have therapeutic potential in patients with cardiovascular disease.

In this study, we applied a metabolomics approach to interrogate differences in the secretome profiles of biologically distinct AT depots collected from patients with atherosclerosis. We revealed AT-secreted metabolites that are dysregulated in obesity, exert endocrine effects on the human vascular wall, and regulate vascular redox state.

## Methods

### Study design

**Figure 1:**
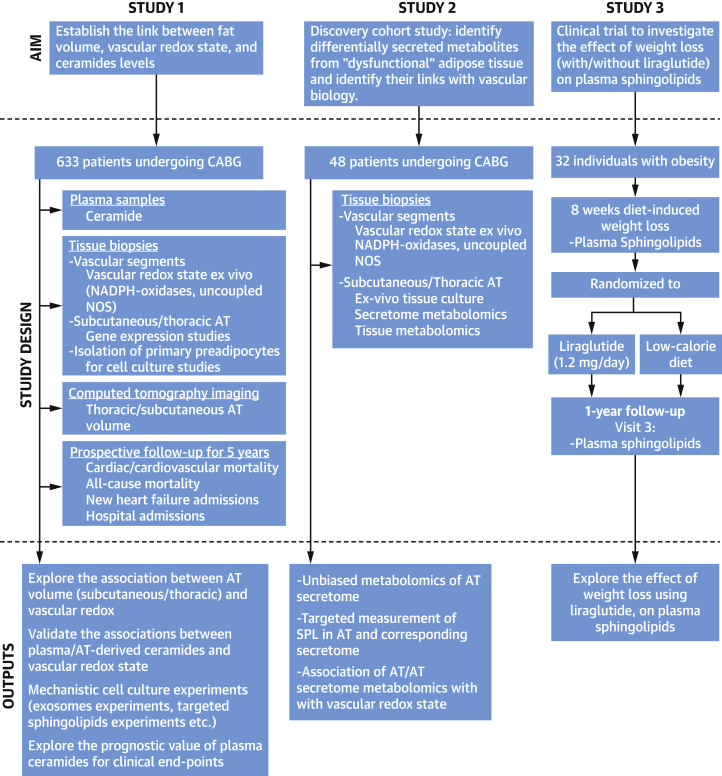
Study Design A schematic diagram depicts the goals, the patient cohorts, and the research methodologies of each study. AT = adipose tissue; CABG = coronary artery bypass graft; NADPH = reduced nicotinamide adenine dinucleotide phosphate; NOS = nitric oxide synthase; SPL = sphingolipid.

The study was approved by the ethics committees in South Central–Oxford C (11/SC/0140) and in Copenhagen (H-4-2010-134). Investigations were conducted according to the principles set forth in the Declaration of Helsinki and European guidelines for clinical research. Informed written consent was obtained from all patients. Study design and experimental details are explained in the Supplemental Methods and illustrated in Figure 1, Supplemental Figure 1, and Supplemental Table 1. Baseline characteristics of study participants are detailed in Supplemental Table 2.

### AT explants

Paired thoracic AT (ThAT) and subcutaneous AT (ScAT) samples were obtained from 48 patients undergoing cardiac surgery and processed as described (3) (Supplemental Methods).

### AT-secreted metabolites profiling

For metabolomics profiling, 4 independent liquid chromatography high-resolution mass spectrometry acquisitions were performed as described (4) (Supplemental Methods).

### Sphingolipid determination

Sphingolipid (SPL) results were validated in AT (n = 96) and the AT secretome (n = 96), as well as in plasma (n = 633), using a targeted liquid chromatography–tandem mass spectrometry platform (Supplemental Methods).

### Vasomotor studies in human vessels

Vascular segments were obtained during coronary artery bypass grafting as described (1). Vasomotor studies were performed on an organ-bath system in which endothelium-dependent and endothelium-independent vasorelaxations were assessed (Supplemental Methods).

### Cell culture studies

Primary adipocytes were isolated from ThAT obtained from patients, and immortalized human aortic endothelial cells (HAECs) and primary HAECs were purchased commercially. Isolation, culturing, differentiation, and treatment of cells are described in the Supplemental Methods.

### Superoxide production measurement

Basal superoxide (O_2_^.−^), reduced nicotinamide adenine dinucleotide phosphate (NADPH)–stimulated O_2_^.−^, and N^G^-nitro-l-arginine methyl ester (L-NAME)–inhibitable O_2_^.−^ production in fresh intact vessels and in HAECs were measured using lucigenin-enhanced chemiluminescence as described (1) (Supplemental Methods).

### Isolation and characterization of extracellular vesicles

Extracellular vesicles were isolated using ultracentrifugation as described (5) (Supplemental Methods).

### Western blot analysis

Cellular protein extracts were analyzed using sodium dodecyl sulfate polyacrylamide-gel electrophoresis on 4% to 12% polyacrylamide gradient gel, transferred to nitrocellulose membranes, and blotted with designated antibodies (Supplemental Methods).

### Biopterin quantification by high-performance liquid chromatography

Tetrahydrobiopterin and oxidized biopterins (7,8-dihydrobiopterin and biopterin) were determined using high-performance liquid chromatography followed by electrochemical and fluorescence detection, respectively, as described (6) (Supplemental Methods).

### Computed tomography imaging

Participants of study 1 underwent noncontrast computed tomographic imaging on a 64-slice scanner (LightSpeed Ultra, GE Healthcare, Little Chalfont, United Kingdom). AT defined as described (7) (Supplemental Methods).

### Cohort-wide gene expression analyses

Total ribonucleic acid was extracted from whole blood, reverse-transcribed, and quantified as described (1) (Supplemental Methods).

### Extraction and definition of endpoints

Collection and classification of mortality data are described in Supplemental Methods.

### Statistical analyses

The differential and enrichment analyses of metabolic data presented in this study were performed using MetaboAnalyst version 4.0 (McGill University, Montreal, Quebec, Canada). SPSS version 25 (IBM, Armonk, New York) was used to perform the statistical analysis.

Non-normally distributed variables are presented as median (interquartile range) and whiskers (Tukey). Normally distributed variables are presented as mean ± SD for large datasets and as mean ± SEM for small datasets. Nonparametric tests were used for non-normally distributed variables. Between-group comparisons of continuous variables were performed using the unpaired Student’s *t*-test for 2 groups (or the nonparametric equivalent Mann-Whitney *U* test) or 1-way analysis of variance (or the nonparametric equivalent Kruskal-Wallis test) for 3 groups followed by calculation of the false discovery rate to correct for multiple testing, as indicated. Paired comparisons were performed using paired Student’s *t*-test (or the nonparametric equivalent Wilcoxon signed rank test), and for 3 or more groups, repeated-measures analysis of variance was used (with Greenhouse-Geisser correction). For between-group serial changes in organ-bath studies, we used 2-way analysis of variance for repeated measures with dose × group interaction terms. All correlations were assessed using bivariate analysis, and Spearman’s rank correlation coefficients were estimated, as indicated in the figure legends.

The prognostic value of ceramides and C16:0-SPL for cardiac or noncardiac mortality was assessed using two multivariate Cox regression models: one after adjustment for age, sex, and smoking and the other with additional adjustment for hypertension, diabetes mellitus, body mass index, total cholesterol, low-density lipoprotein, high-density lipoprotein, and triglycerides. Adjusted hazard ratios are presented per 1-SD increase. The proportional hazard assumption was satisfied for all variables tested in this study, as assessed by the significance of the coefficient of the time-dependent covariates in the Cox models. For the Kaplan-Meier survival curves, we selected the optimal cutoff for circulating ceramide level by identifying the value that maximized Youden’s J statistic (the sum of sensitivity and specificity) on univariate receiver-operating characteristic curve analysis for cardiac mortality to ensure an optimal balance between sensitivity and specificity in our models. Fractional polynomials were also used to model any underlying nonlinear relations of the studied metabolites with time to mortality in adjusted Cox regression models. The linear model was consistently chosen as the best fitting model for all 4 metabolites. All tests were 2-sided, and p values <0.05 were considered to indicate statistical significance. Between-model improvement was tested using the chi-square likelihood ratio test.

## Results

### SPL metabolism is the top-ranked dysregulated metabolic pathway in the secretome of ThAT in obesity

**Figure 2:**
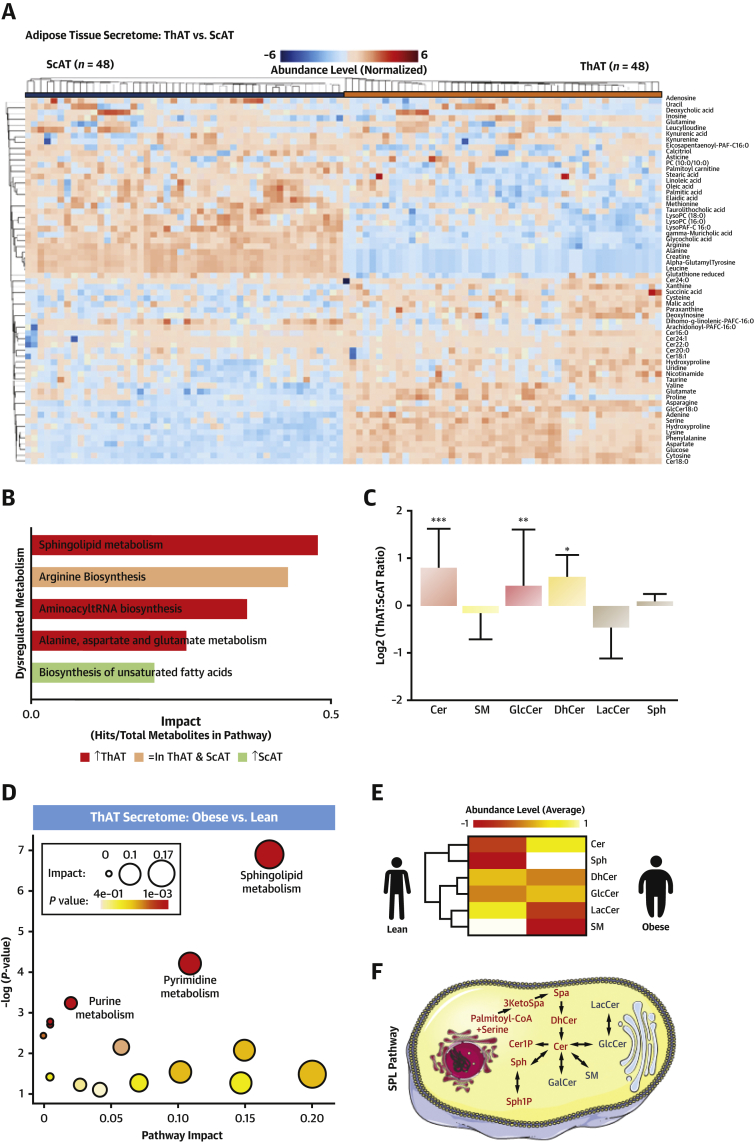
Metabolomics Profiling of AT Secretome **(A)** Heatmap showing the differential secretion patterns for the top 63 differential metabolites (p ≤ 0.001) in thoracic AT (ThAT) versus subcutaneous AT (ScAT) secretome (study 2). **(B)** Pathway enrichment results for the differential metabolites (study 2). The **bar chart** shows the pathways with false discovery rate– and Holm-adjusted p values <0.05, ordered on the basis of their impact and colored on the basis of the percentage of up-regulated metabolites in ThAT secretome versus ScAT secretome. **(C)** Significantly enriched metabolic pathways in the ThAT secretome (study 2) of obese patients (n = 31) versus lean counterparts (n = 17). The node color is based on p value, and the node radius is determined on the basis of pathway impact values. **(D)** Fold change of relative abundance of all measured sphingolipid species containing an 18:1-sphingoid backbone comparing significant enrichment of ceramides in ThAT versus ScAT secretome (study 2). Results are presented as median ± interquartile range; ∗p < 0.05, ∗∗p < 0.01, and ∗∗∗p < 0.001, ThAT versus ScAT. **(E)** Heatmap representing average amounts of sphingolipids in ThAT secretome (study 2) of obese (n = 31) and lean (n = 17) patients. **(F)** Sphingolipid pathway. Cer = ceramide; Cer1P = ceramide-1-phosphate; DhCer = dihydroceramide; GlcCer = glycosylceramide; 3KetoSpa = 3-ketosphinganine; LacCer = lactosylceramide; SM = sphingomyelin; Spa = sphinganine; Sph = sphingosine; Sph1P = sphingosine-1-phosphate; tRNA = transfer ribonucleic acid; other abbreviations as in Figure 1.

We prospectively recruited 633 patients undergoing cardiac surgery at Oxford University Hospitals (Ox-HVF cohort, study 1) (Supplemental Table 2). Vascular O_2_^.−^ was measured in internal mammary arteries and saphenous veins harvested during surgery. In a subgroup of these patients (n = 87), the volumes of ScAT and ThAT (thoracic-visceral AT) were quantified using computed tomography. Bivariate analysis revealed a positive correlation between internal mammary artery resting O_2_^.−^ and both ScAT (rho = 0.234, p = 0.029) and ThAT (rho = 0.269, p = 0.03) volume. However, only ThAT volume was found to be positively related with O_2_^.−^ production in internal mammary arteries after adjustment for traditional risk factors, suggesting a link between dysfunctional ThAT and vascular redox state (Supplemental Figure 2). We then organized a nested study (study 2) in which we cultured 48 paired biopsies of ScAT and ThAT from 31 obese and 17 propensity-matched nonobese patients for 4 h and used the conditioned media for secretome studies (study 2) (Supplemental Table 2). We performed hypothesis-free metabolomics profiling of the AT secretome to identify differentially secreted metabolites related to the AT depot type and the obesity status of the patients. We identified 115 metabolites (54 polar and 61 nonpolar) from ThAT and 116 metabolites (57 polar and 59 nonpolar) from ScAT according to their accurate mass, tandem mass spectrometric pattern, and retention time (Supplemental Table 3) (4). Paired analysis revealed 82 differentially secreted metabolites between the 2 depots (false discovery rate–adjusted p < 0.05) (Figure 2A, Supplemental Table 4, Supplemental Figure 3A). These included amino acids and their by-products (e.g., arginine, serine, glutamine, proline), purines and pyrimidines nucleotides (e.g., adenine, cytosine, uracil), intermediates of glucose and hepatic metabolism (e.g., citric acid, glucose, glycocholic acid), and different lipids (e.g., fatty acids, SPLs).

Functional enrichment analysis mapped the differential AT-secreted metabolites to 20 metabolic pathways (Supplemental Figure 3B). Secreted metabolites were overrepresented in 5 main metabolic pathways (false discovery rate– and Holm-adjusted p < 0.05), including SPL metabolism, arginine biosynthesis, aminoacyl transfer ribonucleic acid biosynthesis, alanine, aspartate and glutamate metabolism, and biosynthesis of unsaturated fatty acids (Figure 2B, Supplemental Figure 3B). As SPL was the top up-regulated metabolism in ThAT, we investigated all SPL species detected in the secretomes of both depots, revealing that ceramides, glycosylceramides, and dihydroceramides were significantly higher in the secretome of ThAT than in that of ScAT (Figure 2C).

To identify dysregulated metabolic pathways in the AT secretome in patients with obesity, we identified metabolites that were differentially secreted from ThAT and ScAT of the obese versus lean participants from study 2 (matched for age, sex, and risk factors) (Supplemental Figure 4). SPL metabolism scored the highest pathway impact in obesity (Figure 2D). Obesity was also related with higher secretion of ceramides from ThAT (Figures 2E and 2F). Hence, we tested the expression of key enzymes involved in ceramide biosynthesis in ThAT biopsies collected from patients in study 1 (Supplemental Results), and we detected a significant obesity-related down-regulation of ceramide kinase (Supplemental Figure 5). In conclusion, the unbiased metabolomic exercise revealed that SPLs were significantly higher in the ThAT secretome compared with the ScAT secretome, a difference driven by the higher levels of ceramides. SPLs were also higher in the secretome of ThAT from obese versus lean subjects, with the ceramides again driving these differences.

### Differential secretion and production levels of ceramides between ThAT and ScAT

**Figure 3:**
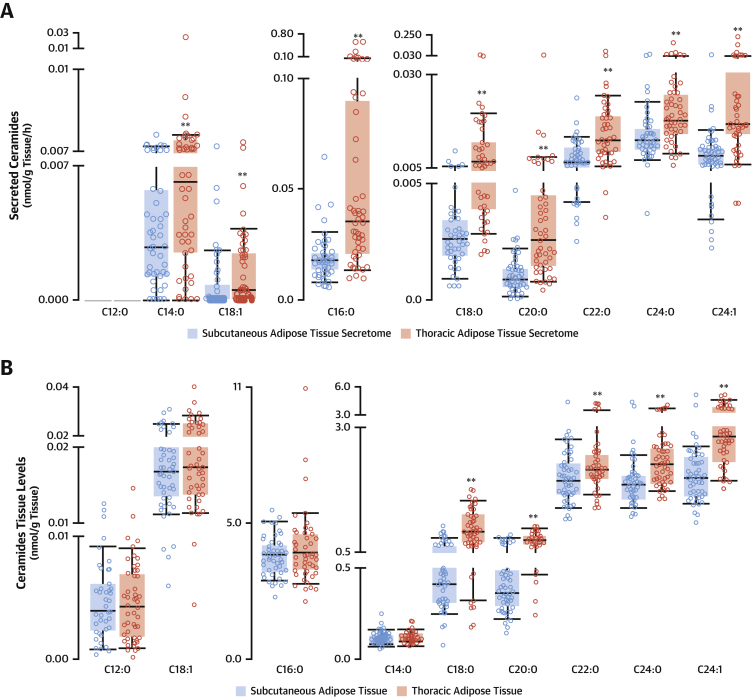
Differential Synthesis and Secretion Patterns of Ceramides in ThAT Versus ScAT Ceramides concentrations were measured in ThAT and ScAT (study 2; n = 96) and their secretome (study 2; n = 96) by liquid chromatography–tandem mass spectrometry. Ceramide secretion **(A)** and synthesis **(B)** pattern in ThAT versus ScAT. Box plots represent median (interquartile range); ∗∗p < 0.05 (false discovery rate adjusted), ThAT versus ScAT (calculated using the Wilcoxon signed rank test). Abbreviations as in Figure 2.

We next explored the role of ThAT-derived SPLs as endocrine regulators of vascular function. We measured the concentrations of all commonly detected SPL metabolites using a targeted platform in the secretome of ThAT and ScAT samples from study 2. Using this additional quantitative approach, we validated the results from the metabolomics screening and also extended the total number of measured SPL species (Figure 3, Supplemental Figure 6). We found that C16:0-ceramide (Cer16:0) (Figure 3A) and other C16:0-SPLs (Supplemental Figures 6A to 6E), namely, C16:0-glycosylceramide (GlcCer16:0) (Supplemental Figure 6C), C16:0-lactosylceramide (Supplemental Figure 6D), and C16:0-dihydroceramide (Supplemental Figure 6B), but not C16:0-sphingomyelin (Supplemental Figure 6A), were the most abundant secreted fatty-acyl chains in ceramides in these AT depots, with levels of Cer16:0 and GlcCer16:0 being 3 times greater in the ThAT secretome.

We next quantified the levels of SPLs in AT biopsies (Figure 3B, Supplemental Figures 6F to 6J) to determine the relationship between SPL levels in AT and SPL concentrations in the AT secretome. We confirmed that C16:0-SPLs were the most abundant species in AT (Figure 3B, Supplemental Figures 6F to 6J). The levels of GlcCer16:0 were 2-fold higher in ThAT compared with ScAT (Supplemental Figure 6H), whereas the levels of Cer16:0 (Figure 3B), C16:0-lactosylceramide (Supplemental Figure 6I), C16:0-dihydroceramide (Supplemental Figure 6G), and C16:0-sphingomyelin (Supplemental Figure 6F) were not significantly different between the 2 depots. This suggests differential production and secretion of GlcCer16:0 in ThAT versus ScAT, but only differential secretion of almost all the other C16:0-SPL species in ThAT versus ScAT. Sphingosine was also identified among the differentially secreted, but not differentially produced, metabolites in ThAT versus ScAT (Supplemental Figures 6E and 6J). Importantly, there was no significant association between triglycerides and Cer16:0 levels in the secretome of ThAT (rho = 0.163, p = 0.267) or ScAT (rho = 0.171, p = 0.239). This finding suggests that the differences in Cer16:0 secreted levels observed between ThAT and ScAT are not due to general leakage of fat from these tissues.

### ThAT primary adipocytes produce and release SPLs via extracellular vesicles

**Figure 4:**
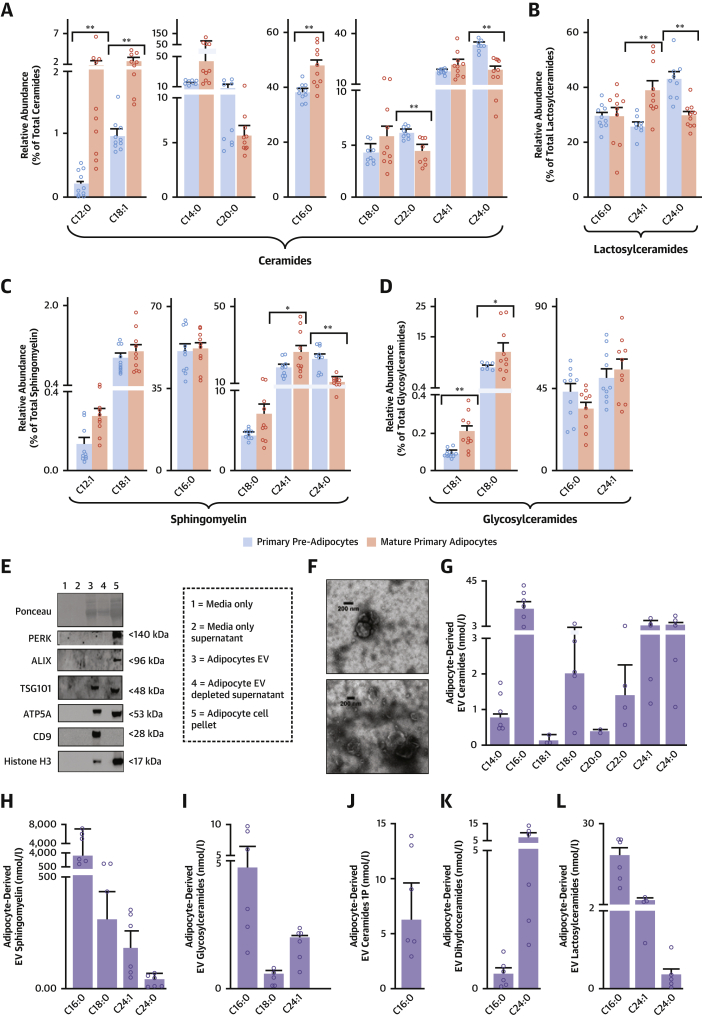
Human Primary Adipocytes Produce SPLs and Secrete Them via EVs SPL intracellular levels in differentiated adipocytes and pre-adipocytes isolated from ThAT biopsies (n = 10 patients) and relative abundance per class **(A to D)**. Adipocyte extracellular vesicles (EVs) (n = 5) were pooled for western blot analysis and were positive for EV markers **(E)**. Adipocyte EVs showed typical morphology by transmission electron microscopy **(F)**. The abundance of each SPL species in EVs released from adipocytes is presented in **G to L**. Bars in **A to D** indicate mean ± SEM; **bars** in **G to L** indicate median ± interquartile range; ∗p < 0.05 (nominal) and ∗∗p < 0.01 (false discovery rate adjusted) for mature adipocytes versus pre-adipocytes (calculated using Wilcoxon signed rank tests). Abbreviations as in Figures 1 and 2.

To examine whether AT SPLs are generated by the adipocytes, we isolated pre-adipocytes from ThAT biopsies of 10 patients undergoing cardiac surgery. We differentiated the pre-adipocytes into mature adipocytes in cell culture and quantified SPL production (Figures 4A to 4D). We observed that both undifferentiated pre-adipocytes and mature adipocytes generated SPLs, with C16:0 species being the most abundant. Differentiated adipocytes produced significantly higher levels of Cer16:0 (Figure 4A), but similar levels of GlcCer16:0 (Figure 4D), compared with the undifferentiated pre-adipocytes. Increased Cer16:0 production was accompanied by a significant reduction in C22:0-ceramide and C24:0-ceramide levels in differentiated adipocytes (Figure 4A), resulting in a >2-fold increase in the respective ratio of Cer16:0 to these species after differentiation (Supplemental Figure 7).

To understand how SPLs are secreted from the human adipocytes, we quantified SPLs in extracellular vesicles from the secretome of ThAT-derived adipocytes (Figures 4E to 4L, Supplemental Figure 8). C16:0-SPLs were the most enriched SPL species in extracellular vesicles, with Cer16:0 being substantially higher compared with other ceramides (Figures 4G to 4L).

### High levels of ThAT-derived SPLs are associated with increased O_2_^.−^ production and impaired endothelial function in human vessels

**Figure 5:**
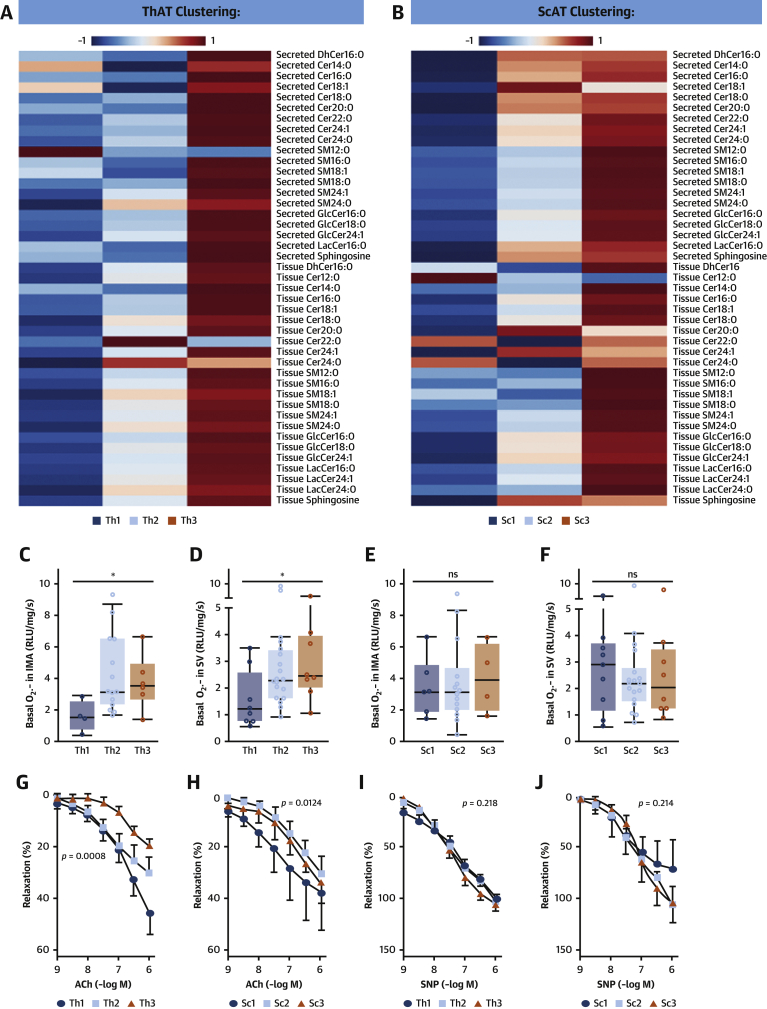
Relationship Between ThAT Ceramides and Vascular Redox State Unsupervised clustering of study 2 patients (n = 48) on the basis of the amounts of SPLs in AT stratified the patients into 3 clusters per tissue: ThAT clusters (Th1, n = 11; Th2, n = 25; Th3, n = 12) and ScAT clusters (Sc1, n = 11; Sc2, n = 26; Sc3, n = 11). Relevant heatmaps showing the intensities of secreted and produced SPLs in the clusters of ThAT **(A)** and ScAT **(B)**. High SPL synthesis in ThAT **(C,D)**, but not in ScAT **(E,F)**, were related with high vascular superoxide (O_2_^.−^) production in both internal mammary arteries (IMAs) and saphenous veins (SV) obtained from the same patients. Similarly, impaired vasorelaxations of human vessels in response to acetylcholine (ACh) were observed in patients with high SPLs in ThAT **(G)**, while there was a similar (but borderline) trend for SPLs in ScAT **(H)** from the same patients, suggesting that high AT SPL levels are related with reduced nitric oxide (NO) bioavailability in vessels. On the contrary, NO-independent vasorelaxations in response to sodium nitroprusside (SNP) vasorelaxations were not related with SPL levels in either ThAT **(I)** or ScAT **(J)**. P values in **C to F** were calculated using the Kruskal-Wallis test. The p values in **G to J** were calculated using 2-way analysis of variance with dose × group interaction terms. RLU = relative light units; other abbreviations as in Figures 1 and 2.

To examine whether AT SPLs affect vascular tone and endothelial function in human vessels, we clustered the SPL profiles of the patients in study 2 on the basis of their intensities using an unsupervised self-organizing map, as illustrated by the heatmaps in Figures 5A and 5B. The algorithm clusters of SPLs produced in ThAT (Figure 5A, Supplemental Figure 9A) and ScAT (Figure 5B, Supplemental Figure 9B) correlated with nearly all ceramides secreted from these tissues (Supplemental Figures 9C and 9D). We explored the association between these clusters and O_2_^.−^ production in human internal mammary arteries and saphenous veins from the same patients. Increased ThAT SPL was correlated with increased O_2_^.−^ generation in human arteries (Figure 5C) and veins (Figure 5D), whereas no such correlation was observed for ScAT SPL (Figures 5E and 5F). Furthermore, high SPL content prominently in ThAT was associated with reduced endothelium-dependent vasorelaxation of human vessels to acetylcholine (Figures 5G and 5H), whereas the endothelium-independent vasorelaxations to sodium nitroprusside were not significantly different between the SPL clusters from either ThAT or ScAT (Figures 5I and 5J), indicating an association between high AT SPL and reduced endothelial nitric oxide bioavailability.

### Elevated levels of plasma Cer16:0 are associated with increased arterial oxidative stress, endothelial dysfunction, and systemic inflammation in humans

To further investigate the role of SPLs as endocrine mediators linking ThAT with the vascular disease pathogenesis, we performed a comprehensive high-throughput quantification of SPL metabolites in 633 plasma samples from patients in study 1 (Supplemental Table 5). The SPLs detected in the plasma of these patients and their circulating concentrations are shown in Supplemental Figure 10 and Supplemental Table 5. Very long chain ceramides (≥20 carbons) were the most abundant ceramides in plasma, yet specific Cer16:0 metabolites were still detected in high concentrations, although they were no longer the dominant species, as was the case for the AT. C16:0-SPL were also enriched in circulating extracellular vesicles (Supplemental Figure 11).

**Figure 6:**
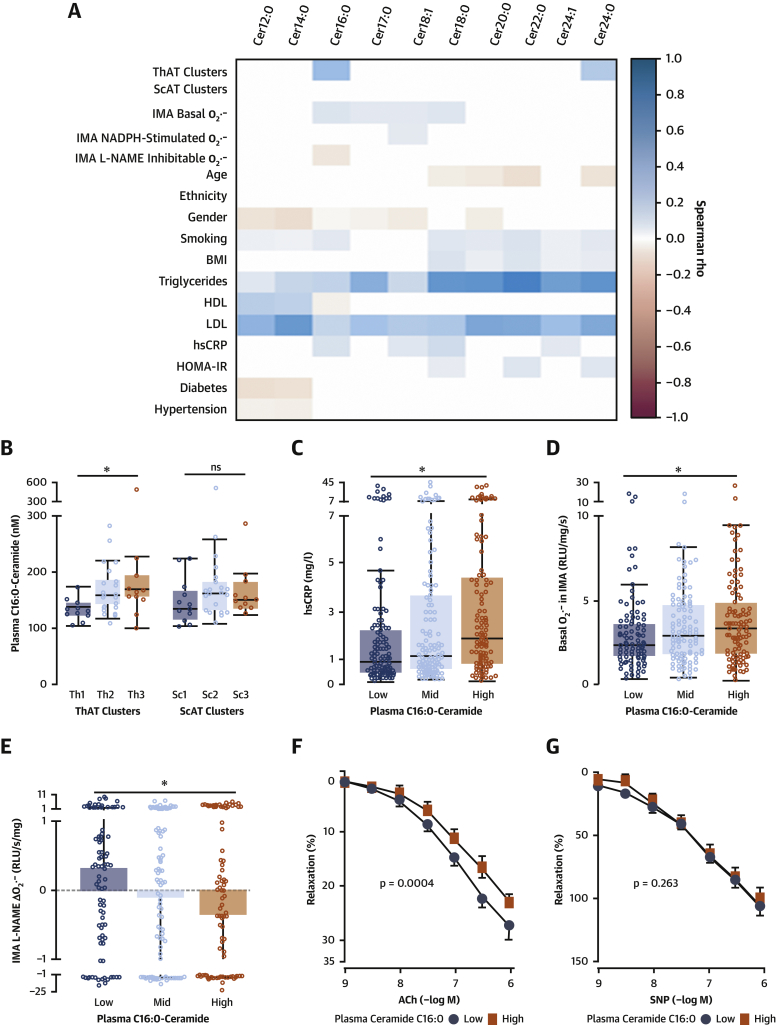
Circulating Cer16:0 Associated With Vascular Redox State Univariate correlations between plasma levels of 10 quantified ceramides, vascular redox state, and risk profile of patients in study 1 **(A)**. Plasma C16:0-ceramide (Cer16:0) levels were significantly related with ceramide levels in ThAT but not ScAT **(B)**. Association between plasma Cer16:0 levels and serum high-sensitivity C-reactive protein (hsCRP) levels (**C**; n = 351), O_2_^.−^ production in human IMAs (**D**; n = 309), endothelial NO oxide uncoupling determined by the N^G^-nitro-l-arginine methyl ester (L-NAME)–inhibitable O_2_^.−^ (**E**; n = 269), and reduced endothelial NO bioavailability measured by the ex vivo vasorelaxations in response to ACh (**F**; n = 128). There was no association between plasma Cer16:0 and endothelium-independent vasorelaxations of human vessels to SNP (**G**; n = 123). P values were calculated using the Kruskal-Wallis test **(B–D)** or 2-way analysis of variance with dose × group interaction terms **(F,G)**. BMI = body mass index; HDL = high-density lipoprotein; HOMA-IR = homeostasis model assessment of insulin resistance; LDL = low-density lipoprotein; other abbreviations as in Figures 1, 2, and 5.

We then explored the associations between AT and circulating ceramides and key mechanisms of vascular disease pathogenesis (Figure 6A, Supplemental Figures 12A to 12H). Cer16:0 was significantly associated with elevated concentrations of ceramides in ThAT but not in ScAT (Figures 6A and 6B), implying that ThAT may drive the increased plasma levels of Cer16:0. Circulating Cer16:0 was also found to be positively associated with high-sensitivity C-reactive protein (Figures 6A and 6C). Plasma Cer16:0 was associated with increased O_2_^.−^ production (Figures 6A and 6D) and with endothelial nitric oxide synthase (eNOS) uncoupling, as determined by L-NAME-inhibitable O_2_^.−^ in human arteries (Figures 6A and 6E). However, plasma Cer16:0 levels were not correlated with NADPH-stimulated O_2_^.−^ generated by NADPH oxidases in patients’ internal mammary artery segments (Figure 6A, Supplemental Figure 12I). Circulating Cer16:0 was also associated with reduced vasorelaxation of human vessels in response to acetylcholine (but not to sodium nitroprusside) (Figures 6F and 6G), suggesting that high levels of circulating Cer16:0 are related with impaired nitric oxide bioavailability in human vessels.

### Direct effects of Cer16:0 on endothelial redox state

**Figure 7:**
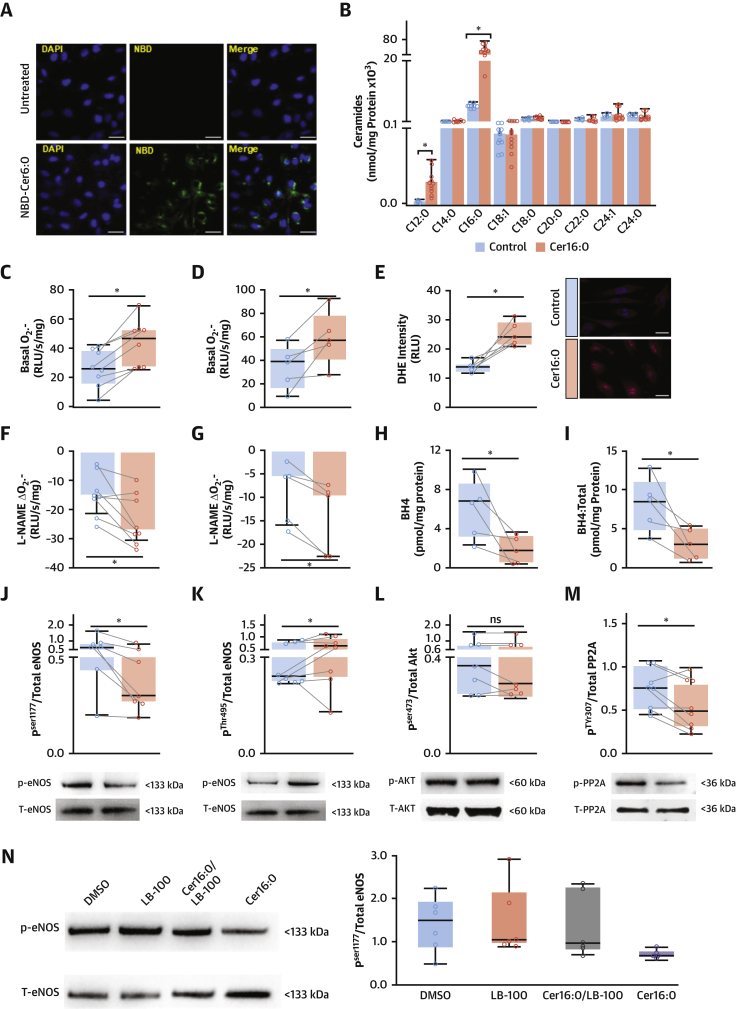
Effects of Exogenous Cer16:0 on Redox State and eNOS Coupling of Human Aortic Endothelial Cells Confocal microscope images of NBD-Cer6:0 **(green, A)**. Cer16:0 treatment significantly increased Cer16:0 in immortalized human aortic endothelial cells (teloHAECs) (**B**; n = 11), resulting in increased intracellular O_2_^.−^ production in teloHAECs (**C**; n = 9) and primary human aortic endothelial cells (HAECs) (**D**, n = 5 different donors). Representative teloHAECs stained with dihydroethidium (DHE) show increased intensity of O_2_^.−^ fluorescence in treated cells (**E**; n = 10). Treatment of teloHAECs (**F**; n = 9) and primary HAECs (**G**; n = 5) with Cer16:0 increased the L-NAME-inhibitable O_2_^.−^, via reduction of intracellular tetrahydrobiopterin (BH4) (**H**; n = 5) and BH4/total biopterin ratio (**I**; n = 5). Treatment of teloHAECs (n = 7 or 8) with Cer16:0 reduced endothelial NO synthase (eNOS) phosphorylation on activation site Ser1177 **(J)** and increased its phosphorylation at the inhibitory site Thr495 **(K)**. Although there was no effect of Cer16:0 on AKT phosphorylation at site Ser473 **(L)**, it induced significant reduction of protein phosphatase 2 (PP2A) phosphorylation at its inhibitory site Tyr307 **(M)**. The Cer16:0-induced reductions in p-eNOS^Ser1177^/eNOS ratio was reversed by 10 μM LB100, a PP2A inhibitor (**N**; n = 6). ∗p < 0.05 versus control (DMSO <1%); all presented as median (interquartile range). Abbreviations as in Figures 1, 2, 5, and 6.

To explore the potential causal effect of Cer16:0 on vascular endothelial dysfunction, we first investigated whether ceramides are taken up by endothelial cells. We exposed immortalized HAECs to NBD-Cer6:0, a fluorescent analogue of ceramide, and examined cells under a confocal microscope. The fluorescently labeled ceramide accumulated intracellularly (Figure 7A). Treatment of cells with natural Cer16:0 resulted in a significant increase in the levels of intracellular Cer16:0 after treatment, as confirmed by liquid chromatography–tandem mass spectrometry (Figure 7B). Cer16:0 treatment of both immortalized HAECs (Figure 7C) and primary HAECs (Figure 7D) significantly increased O_2_^.−^ production. This was confirmed using dihydroethidium staining of these cells (Figure 7E).

Next, to determine the mechanism by which Cer16:0 increases vascular O_2_^.−^ production in human vessels, we explored whether the association observed across the cohort (Figure 6A) between Cer16:0 and eNOS uncoupling is causal. We performed a series of in vitro experiments and found that Cer16:0 induced eNOS uncoupling in both immortalized HAECs (Figure 7F) and primary HAECs (Figure 7G), as evidenced by reduced L-NAME-inhibitable O_2_^.−^. Exogenous treatment of the primary HAECs with a naturally occurring ceramide of a medium-length acyl chain (C12:0-ceramide) had a borderline, nonsignificant effect on basal O_2_^.−^ or L-NAME inhibitable O_2_^.−^ in these cells (Supplemental Figure 13). To understand how Cer16:0 induces eNOS uncoupling, we evaluated its effects on the intracellular levels of the eNOS cofactor tetrahydrobiopterin. Cer16:0 reduced intracellular tetrahydrobiopterin levels (Figure 7H) as well as the ratio of tetrahydrobiopterin to total biopterins (Figure 7I), suggesting that Cer16:0 induces tetrahydrobiopterin oxidation. Following this observation, we examined whether Cer16:0 had any effect on eNOS activation by exploring its impact on the phosphorylation of its activation (Ser1177) and inhibitory (Thr495) sites. Notably, Cer16:0 reduced the phosphorylation of eNOS^Ser1177^ (Figure 7J, Supplemental Figures 14 and 15) and increased the phosphorylation of eNOS^Thr495^ (Figure 7K). To understand how Cer16:0 regulates eNOS phosphorylation, we then demonstrated that although Cer16:0 had no effect on the activation of Akt^Ser473^ (Figure 7L), it increased the activity of protein phosphatase 2 (PP2A) in HAECs, by suppressing the phosphorylation of its inhibitory site PP2A^Tyr307^ (Figure 7M). We then used LB100, a potent inhibitor of PP2A activity, which prevented Cer16:0-induced reductions in p-eNOS^Ser1177^:eNOS (Figure 7N).

### Plasma Cer16:0 levels predict cardiac mortality

**Figure 8:**
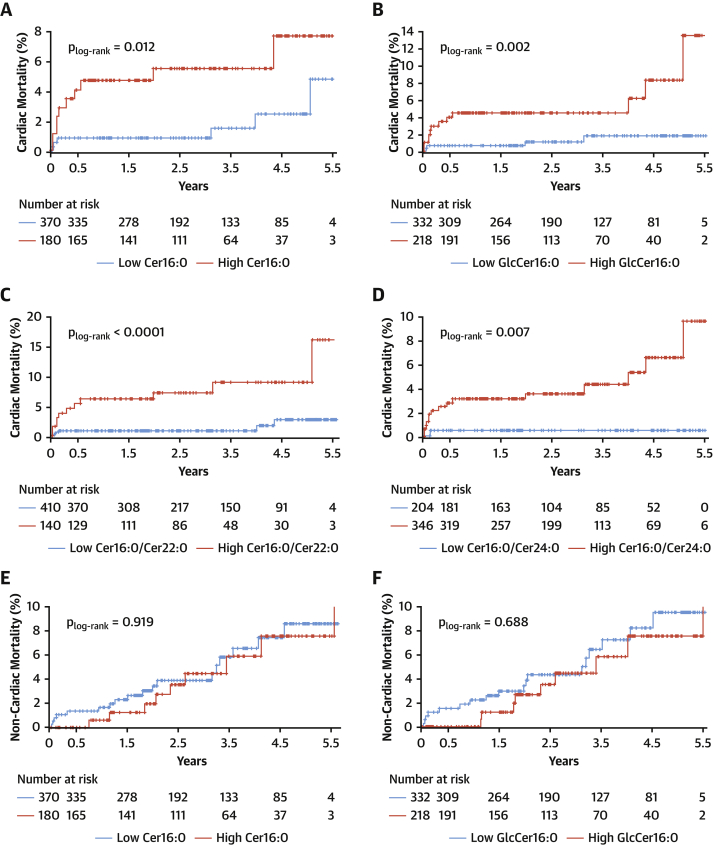

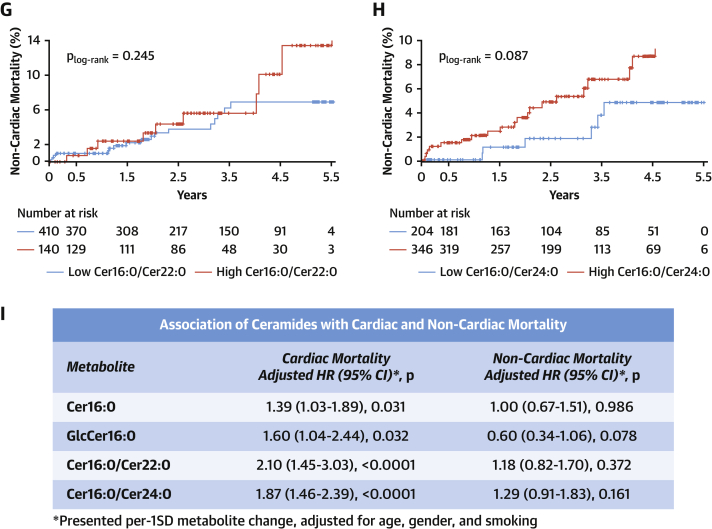
Plasma Cer16:0 and GlcCer16:0 Are Modifiable Risk Biomarkers Kaplan-Meier estimates of cumulative probability of cardiac death by years of follow-up and levels of circulating C16:0-ceramide (Cer16:0) **(A)**, C16:0-glycosylceramide (GlcCer16:0) **(B)**, Cer16:0/C22:0-ceramide (Cer22:0) **(C)**, and Cer16:0/C24:0-ceramide (Cer24:0) **(D)** and estimates of noncardiac death by years of follow-up and levels of circulating Cer16:0 **(E)**, GlcCer16:0 **(F)**, Cer16:0/Cer22:0 **(G)**, and Cer16:0/Cer24:0 **(H)**. Log-rank significance values are presented for the plots. **(I)** Adjusted hazard ratios (HRs) with 95% confidence intervals (CIs) derived from Cox regression after correction for age, sex, and smoking status.

We next explored whether these newly described causal effects of Cer16:0 on vascular dysfunction would translate to clinical phenotypes associated with vascular disease outcomes. The population of study 1 was followed for up to 5.5 years. There were 44 deaths in the cohort, 17 of which were cardiac related. Kaplan-Meier survival functions were evaluated for these metabolites with cardiac (Figures 8A to 8D) and noncardiac mortality (Figures 8E to 8H) and were compared statistically using the log-rank test (Figures 8A to 8H) after ensuring the absence of competing risk (data not shown). The cutoff value for each ceramide species was identified from univariate analysis (Youden index) (Supplemental Table 6). The concentrations of Cer16:0, GlcCer16:0, Cer16:0/C22:0-ceramide, and Cer16:0/C24:0-ceramide were significant predictors of cardiac death (Supplemental Figure 16, Supplemental Table 7, Figure 8). Cox regression was performed to define the predictive value of each ceramide for mortality, after adjustment for age, sex, and smoking (Figure 8F) plus body mass index, cholesterol, low-density lipoprotein, high-density lipoprotein, triglycerides, hypertension, and diabetes (Supplemental Table 7). The tested metabolites were also found to be associated with cardiovascular death (Supplemental Tables 8 and 9), adverse cardiac phenotypes, and endpoints (Supplemental Figure 17, Supplemental Table 10). There was no association between these metabolites and noncardiac mortality in the same cohort (Figure 8I).

### Plasma Cer16:0 levels are modifiable by the glucagon-like peptide-1 agonist liraglutide

**Figure 9:**
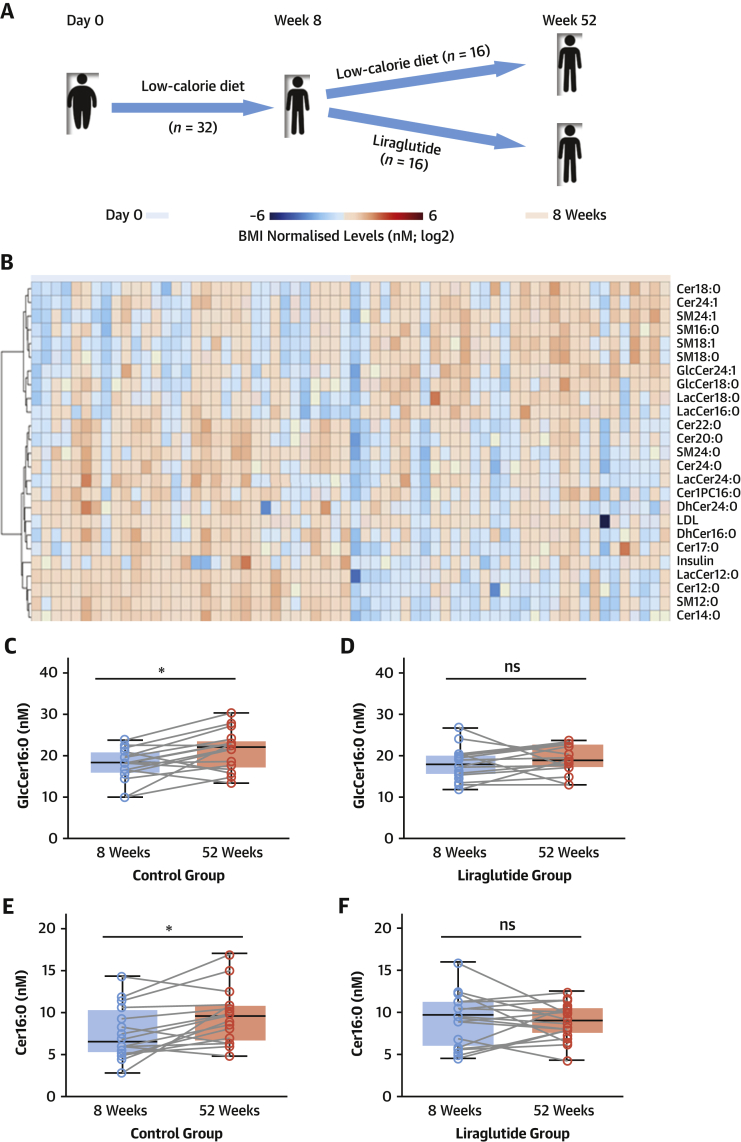
Plasma Cer16:0 and GlcCer16:0 Are Modifiable Risk Biomarkers **(A)** A schematic representation of the randomized controlled trial design. **(B)** A heat map showing the significant differential metabolites between day 0 and week 8. Box plots represent changes in GlcCer16:0 **(C,D)** and Cer16:0 **(E,F)** body mass index–normalized concentrations in patients who were randomized to a low-calorie diet or liraglutide treatment (1.2 mg/day) for 52 weeks, following 8 weeks (baseline) of low-calorie diet–induced weight loss. Box plots represent median (interquartile range). Abbreviations as in Figure 8.

We then explored whether treatments that are known to reprogram AT biology (such as a low-calorie diet with or without glucagon-like peptide-1 [GLP-1] analogs) could also affect Cer16:0 levels. We explored whether plasma SPLs, specifically Cer16:0, are amenable to treatment by the GLP-1 analog liraglutide, in a randomized clinical trial (NCT02094183) (Figure 9A) (8).We measured the circulating levels of SPLs in 32 obese patients (average body mass index 33.5 ± 2.5 kg/m^2^ at inclusion) (study 3 in Supplemental Table 2). Before randomization to treatment, all participants adhered to an 8-week low-calorie diet (800 kcal/day), during which they lost 12.8 ± 6.0 kg of body weight, exhibiting significant reductions in body mass index (p < 0.0001), which were paralleled by significant effects on the circulating levels of SPLs (levels of 27 of 34 SPLs changed, with false discovery rate–adjusted p values <0.05, after correcting for body mass index) (Figure 9B, Supplemental Table 11). Following this initial phase, the patients were randomized to liraglutide treatment (1.2 mg/day) or no treatment for a period of 52 weeks (Figure 9A). Liraglutide treatment differentially affected several SPL species compared with the control group (Supplemental Table 12, Figures 9C to 9F, Supplemental Figure 18) despite no significant changes in body mass index in either of the two groups. Of these, Cer16:0 and GlcCer16:0 were significantly increased in the control group after 52 weeks, while they remained unchanged in the liraglutide-treated group (Figures 9C to 9F), suggesting that metabolic reprogramming of AT by targeting GLP-1 signaling, may reduce cardiovascular risk by modifying circulating Cer16:0.

## Discussion

In this study, we used an unbiased metabolomics approach to identify AT-derived metabolites with endocrine effects upon vascular health. A summary of study tests and key findings are shown in Supplemental Table 13 and the Central Illustration.

**Central Illustration:**
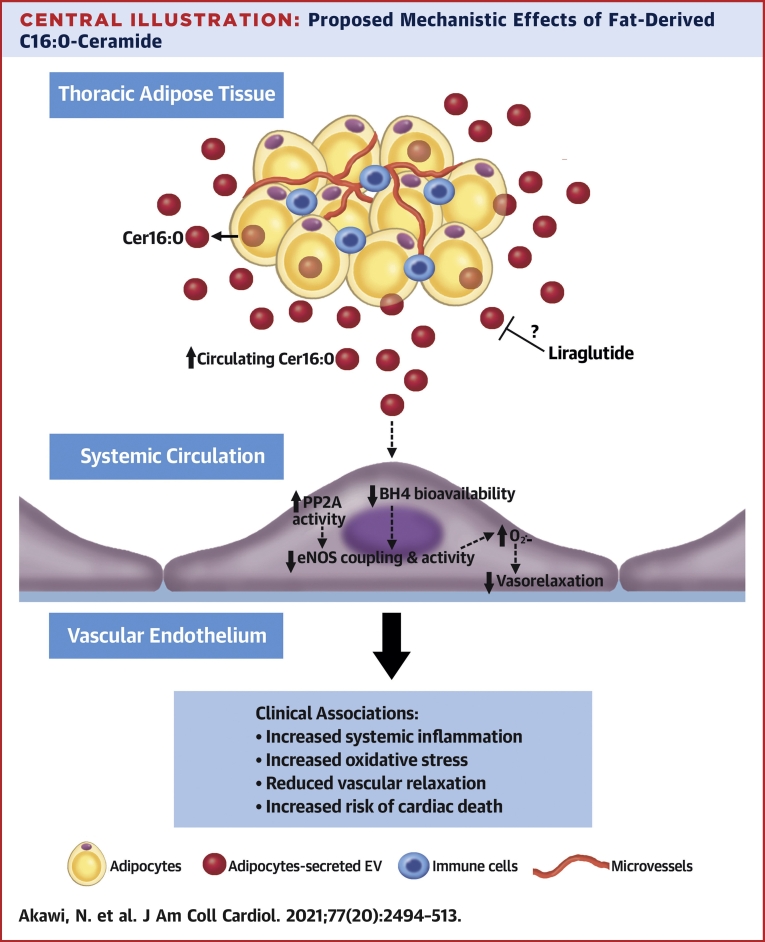
Proposed Mechanistic Effects of Fat-Derived C16:0-Ceramide Dysfunctional thoracic adipose tissue in obese cardiac patients secretes higher levels of C16:0-ceramide (Cer16:0), increasing its circulating levels. Acting via endothelial nitric oxide synthase signaling, circulating Cer16:0 enhances systemic inflammation, amplifies oxidative stress, and reduces endothelium-dependent vasorelaxation, eventually leading to adverse cardiac outcomes including death.

Ceramides have been recognized as key signaling molecules in different cell types regulating vital cellular processes. The origin of circulating ceramides in humans is poorly understood, although the liver and AT might be key sources (9,10). We now demonstrate that SPL is the most prominent dysregulated pathway in obese ThAT compared with ScAT. Cer16:0 is the most abundant ceramide generated in ThAT, and it is secreted largely in extracellular vesicles released from the adipocytes (Supplemental Figures 19 and 20). The secreted Cer16:0 levels from ThAT (but not ScAT) are correlated with plasma Cer16:0 levels, implying that ThAT may contribute to circulating Cer16:0 levels, although this association is suggestive and not conclusive.

Ceramides and their derivatives have been linked with cardiovascular disease (11,12). In particular, plasma Cer16:0 has been associated with increased risk for major adverse cardiovascular events, including heart failure (13,14) and myocardial infarction (11,15), in the general population. However, it is unclear whether AT-secreted ceramides play a role in the vascular complications of obesity. We now demonstrate that ThAT volume is associated with increased arterial oxidative stress and that this depot secretes more ceramides than ScAT, particularly Cer16:0. ThAT-derived ceramide levels were also related with vascular redox state.

Increased vascular oxidative stress and reduced endothelial nitric oxide bioavailability are key features of atherogenesis (16). Nitric oxide is produced by eNOS, an enzyme that becomes a source of O_2_^.−^ when its cofactor tetrahydrobiopterin is oxidized (6). It is unclear whether AT-derived ceramides play a role in the regulation of these fundamental mechanisms of atherogenesis. We now reveal using ex vivo human tissue and in vitro primary cultured cells that increased levels of Cer16:0 in the secretome of ThAT cause arterial oxidative stress. This effect was observed with long-chain ceramides in the initial association screening, but not with medium-chain ceramides such as C12:0-ceramide. We also show that Cer16:0 induces eNOS uncoupling via tetrahydrobiopterin oxidation, while it affects eNOS activity via PP2A. These findings suggest that Cer16:0, the most abundant ceramide in ThAT secretome, has direct effects on the mechanisms of atherogenesis.

Although our findings suggest causal links between ceramides and vascular dysfunction, existing data on the prognostic role of circulating ceramides in humans remain unclear, with some studies reporting positive (11,12) and others negative (17) results. Laaksonen et al. (12) have shown that Cer16:0 predicts cardiac death in large-scale cardiac angiography cohorts with a hazard ratio (per 1-SD increase) of 1.98 (95% confidence interval: 1.49 to 2.62; p < 0.001). Here, we have measured circulating ceramide levels in 633 patients with advanced atherosclerosis who underwent cardiac surgery and demonstrated that plasma Cer16:0 independently predicts cardiac mortality in a similar way (hazard ratio per 1-SD increase: 1.39; 95% confidence interval: 1.03 to 1.89; p = 0.031). Patients with high glycosylated Cer16:0 have 2-fold higher risk for a fatal cardiac event over a follow-up period of 5.5 years, independent of any clinical risk factors.

Promising therapeutic approaches specifically targeting ceramides biosynthesis have been reported (18,19), but it is unknown whether ceramides could be modified by therapeutic interventions that are known to reprogram AT and to reduce cardiovascular disease risk, such as GLP-1 analogs (20). We explored the effects of liraglutide on the ceramide profile in a randomized trial in patients with obesity. Indeed, 52 weeks’ treatment with liraglutide, following 8 weeks of intense, low-calorie diet–induced weight loss, led to better control of Cer16:0 and GlcCer16:0 plasma levels compared with the control group, confirming that ceramides are modifiable therapeutic targets in obesity. This may partly explain the improved cardiovascular disease outcomes observed in patients treated with liraglutide in the LEADER clinical trial (20).

### Study limitations

The metabolomics methodology used in this study confidently screened for 413 pre-determined polar and nonpolar metabolites, and identification of additional metabolites and lipids would require different platforms. Another limitation of this study was the small number of events in the outcomes part of the study, which may have prevented additional ceramides with smaller effect sizes on prognosis from reaching statistical significance. In addition, this study lacked an external validation cohort as well as computed tomographic scans quantifying the volume of other fat depots, such as the gluteal fat. Further mechanistic investigations are needed to confirm the effect of human ThAT secretome–derived Cer16:0 on endothelial cells.

Perspectives **COMPETENCY IN MEDICAL KNOWLEDGE:** Metabolic crosstalk between the cardiovascular system and AT is crucial to normal function of both. Dysregulation of fat-derived SPL in obese patients with cardiovascular disease reflects plasma levels of Cer16:0, an independent predictor of mortality.

**TRANSLATIONAL OUTLOOK:** Further studies are needed to clarify the roles of Cer16:0 and its glycosylated derivative in preventing adverse outcomes among patients with atherosclerosis managed with weight loss and treatment with liraglutide.

## Conclusions

We demonstrate the ThAT secretes ceramides, and particularly Cer16:0, which has an endocrine effect on the vascular wall, inducing vascular redox state. We also show that high plasma levels of Cer16:0 have striking prognostic value for cardiac mortality, and may be a drugable target of GLP1-analogues.

## Funding Support and Author Disclosures

This study was supported by the Novo Nordisk Foundation Tripartite Immunometabolism Consortium Award (NNF15CC0018486), the British Heart Foundation (FS/16/15/32047, PG/13/56/30383, RG/17/10/32859 and RG/F/21/110040) and British Heart Foundation Chair award (CH/16/1/32013), British Heart Foundation Centre of Research Excellence award (RG/13/1/30181), the National Institute for Health Research Oxford Biomedical Research Centre, and the Swedish Heart Lung Foundation (HLF 20180290). The authors have reported that they have no relationships relevant to the contents of this paper to disclose.
