# Supplementary material for: Fat-Secreted Ceramides Regulate Vascular Redox State and Influence Outcomes in Patients With Cardiovascular Disease
Source: J Am Coll Cardiol. 2021 May 25;77(20):2494–513. doi: 10.1016/j.jacc.2021.03.314 (PMC8141611; doi:10.1016/j.jacc.2021.03.314)
Supplement: Supplemental Data [file mmc1.docx]

-**ONLINE MATERIAL**-

**Fat-Secreted Ceramides Regulate Vascular Redox State and Influence Outcomes in Patients with Cardiovascular Disease**

**Brief title:** Adipose C16:0-ceramide modulates vascular redox state

Nadia Akawi, PhD*^a, b^, Antonio Checa, PhD*^c^, Alexios S. Antonopoulos, MD, PhD^a^, Ioannis Akoumianakis, MD, PhD^a^, Evangelia Daskalaki, PhD^c^, Christos P. Kotanidis, MD, MSc^a^, Hidekazu Kondo, MD, PhD^a^, Kirsten Lee, MD^a^, Dilan Yesilyurt, MSc^a^, Ileana Badi, PhD^a,^ Murray Polkinghorne, MD, MSc^a^, Naveed Akbar, PhD^a^, Julie Lundgren, PhD^d, e^, Surawee Chuaiphichai, PhD^a^, Robin Choudhury, MD, PhD^a^, Stefan Neubauer, MD, PhD^a^, Keith M. Channon, MD, PhD^a^, Signe S. Torekov, MD, PhD^d, e+^, Craig E. Wheelock, PhD^c+^, Charalambos Antoniades, MD, PhD^a+^

^a^Division of Cardiovascular Medicine, Radcliffe Department of Medicine, University of Oxford, UK

^b^Department of Genetics and Genomics, College of Medicine and Health Sciences, United Arab Emirates University, Al-Ain, UAE

^c^Division of Physiological Chemistry II, Department of Medical Biochemistry and Biophysics, Karolinska Institute, Sweden

^d^NNF Center for Basic Metabolic Research, Faculty of Health and Medical Sciences, University of Copenhagen, Copenhagen, Denmark

^e^Department of Biomedical Sciences, Faculty of Health and Medical Sciences, University of Copenhagen, Copenhagen, Denmark

*^,+^Contributed equally to the study

**Brief title:** Adipose Cer16:0 modifies vascular redox state

**Corresponding author**:

Professor Charalambos Antoniades

Deputy Head, Division of Cardiovascular Medicine

Radcliffe Department of Medicine, Level 6 West Wing

John Radcliffe Hospital, Headley Way, Headington

Oxford OX3 9DU, United Kingdom

Tel: +44(0)1865-228340; Fax: +44(0)1865-234615

E-mail: [antoniad@well.ox.ac.uk](mailto:antoniad@well.ox.ac.uk)

**-ONLINE METHODS & MATERIALS-**

***Materials & Reagents***

Materials reagents and key resources used in this study are listed in Online-Table 1 and in the text.

***Study design***

Study 1 comprised a total of 633 prospectively enrolled patients undergoing coronary artery bypass surgery (CABG) at Oxford University Hospitals, recruited under the Oxford Heart Vessels and Fat (ox-HVF) cohort (Online-Table 2). Exclusion criteria included chronic inflammatory, neoplastic, renal or hepatic diseases. Plasma samples were obtained on the morning of the surgery while tissue samples were harvested during the surgery. Study 1 served as a platform for validation of observational links between AT secretome and parameters of vascular biology, which were then mechanistically interrogated in appropriate cell culture models.

Study 2 included 48 participants of the Oxford Cohort for Heart, Vessels & Fat (OxHVF) study (subgroup of Study 1), 31 obese (BMI ≥ 25 Kg/m^2^) and 17 lean (BMI < 25 Kg/m^2^) matched for age, sex and risk factors (Online-Table 2). A set of 48 paired samples of ThAT from the paracardial region and ScAT from the incision site as well as segments of patient vessels (*i.e.,* IMA and SV) were collected during CABG surgery and used for metabolomics and mechanistic experiments to explore the relationship between AT metabolites and specifically sphingolipid and vascular redox state. Study 2 was used as an exploratory cohort to identify differentially secreted metabolites from “dysfunctional” AT and their links with vascular biology.

Study 3 consisted of 32 healthy obese participants of a randomized placebo-controlled trial (ClinicalTrials.gov Identifier: NCT02094183;

https://clinicaltrials.gov/ct2/show/NCT02094183?term=NCT02094183&draw=2&rank=1) that was conducted previously by Iepsen *et al* (1) to investigate the effect of weight loss and weight maintenance with and without liraglutide treatment on plasma sphingolipid profiles. The effects of liraglutide treatment on a wider range of lipids have previously been published (2).

The studies were conducted according to the principles set forth under the Declaration of Helsinki (1989) and European guidelines for clinical research. Informed written consents were obtained from all patients.

***Adipose tissue explants***

Adipose tissue (AT) biopsies were immediately transported in warm transfer medium to a tissue culture hood, where they were placed in fresh transfer medium and cut into ∼1 mm^3^ pieces. Minced explants were rinsed in PBS and incubated in 1ml Medium-199 per 200mg tissue, for 4 hours at 37°C in 5% CO_2_. Tissues were separated from the medium after the incubation and stored at -80°C. The medium was also stored at -80°C.

***Adipose tissue secreted metabolites profiling***

On the day of analysis, the samples were thawed at 4°C then vortexed for 30 seconds and 50 µl of each sample were transferred to an Eppendorf tube. Next, 150 μl of liquid chromatography–mass spectrometry grade (LC-MS) methanol (Optima, Thermo Fisher Scientific, PN: 10767665) were added to each Eppendorf tube. Samples were then vortexed for 30 seconds and allowed to equilibrate for 10 minutes at room temperature. Afterwards, samples were centrifuged at 12000 *g* for 15 minutes at 4°C. Then, 150 µl of the supernatant were filtered using Ultrafree-MC VV Centrifugal Filters (Merck-Millipore, PN: UFC30VV00) at 5000 *g* for 3.5 minutes. Finally, four independent 30 µl aliquots were transferred to LC-MS vials. The remainder volume of the extracts was pooled for use as an MS injection quality control (QC). After pooling, the QC sample was aliquoted in different LC-MS vials that were injected during the sequence.

LC- high resolution mass spectrometry (LC-HRMS) experiments were performed on a 1290 Infinity II ultra-high performance liquid chromatography (UHPLC) system coupled to a 6550 iFunnel quadrupole-time of flight (Q-TOF) mass spectrometer equipped with a dual AJS electrospray ionization source (Agilent Technologies, Santa Clara, CA, USA). For polar secreted metabolites, separation was carried out on a SeQuant® ZIC®-HILIC (Merck, Darmstadt, Germany, PN: 1.50441.0001) column 100 Å (100 mm × 2.1 mm, 3.5 µm particle size) coupled to a guard column (20 mm × 2.1 mm, 3.5 µm particle size) (also from Merck, PN: 1.50436.0001) and an inline-filter (Agilent, PN: 5023-02771). Mobile phases consisted of 0.1 % formic acid (Optima, Thermo Fisher Scientific, PN: 10596814) in water (solvent A) and 0.1 % formic acid in acetonitrile (solvent B) (Optima, Thermo Fisher Scientific, PN: 10001334). The elution gradient used was as follows: isocratic step at 95 % B for 1.5 minutes, 95 % B to 40 % B in 12 minutes and maintained at 40 % B for 2 minutes, then decreasing to 25 % B at 14.2 minutes and maintained for 2.8 minutes, then returned to initial conditions over 1 minute, and the column was equilibrated at initial conditions for 7 minutes. The flow rate was 0.3 ml · minutes^-1^, injection volume was 5 µl and the column oven was maintained at 25°C.

For non-polar secreted metabolites, separation was carried out on a Zorbax Eclipse Plus C18, RRHD (Agilent Technologies, PN: 959758-902) (100 × 2.1 mm, 1.8 µm particle size) using a guard column (Agilent Technologies, PN: 821725-901) (5 × 2 mm, 1.8 μm particle size) and an inline-filter. Sample analysis in both positive and negative mode was performed using water with 0.1% formic acid (Optima, Thermo Fisher Scientific, PN: 10596814) (solvent A) and 2-propanol and acetonitrile (90:10, v/v) (both Optima, Thermo Fisher Scientific, PN: 10684355 and 10001334) with 0.1% formic acid (solvent B). The gradient elution was set as follows: isocratic step of 5% B for 3 min, 5 to 30% B in 2 min, then B was increased to 98% in 13.5 min, maintained at 98% B for 1.5 min, returned to initial conditions over 0.5 min, and then held for a further 4.5 min. The flow rate was 0.4 ml/min, injection volume was 5 μl, and the column oven was maintained at 50 °C.

For each chromatographic mode, two independent injections were run for positive and negative acquisition modes. The quad time of flight (Q-TOF) system was calibrated and tuned according to the protocols recommended by the manufacturer. Nitrogen (purity > 99.9990 %) was used as a sheath gas and drying gas at a flow of 8 L/minute and 15 L/ minute, respectively. The drying and sheath gas temperature were set at 250°C, with the nebulizer pressure at 35 psi and voltage 3000 V (+/- for positive and negative ionization mode, respectively). The fragmentor voltage was set at 380 V. The acquisition was obtained with a mass range of 50-1200 *m/z* for both chromatographic modes, where full scan high-resolution data were acquired at three alternating collision energies (0, 10 and 30 eV). The data acquisition rate was 6 scans·sec^-1^. Between 0 and 1 minutes, the LC flow was diverted to the waste. For further details regarding the acquisition methodology please see (3).

Samples were processed using ProFinder B6.00.00 version using the in-house generated library with authentic standards (3). Compounds were identified according to their accurate mass and retention time matching to the library. For further confirmation, matching of compounds MS/MS spectra with those of the library was performed. After peak integration, samples were corrected using the quality control-robust spline correction algorithm implemented in MATLAB (Mathworks, R2015a). The number of metabolites and global %CVs detected for each platform are reported in Online-Table 3.

***Adipose tissue secreted sphingolipids validation***

In order to both confirm the results and extend the coverage of the pathway, secreted metabolite samples were reanalysed using a targeted sphingolipid (SPL) platform. On the day of analysis, samples were thawed in the refrigerator at 4°C. After that, samples were vortexed for 30 seconds and 25 µl of each sample was transferred to an Eppendorf tube. Next, 10 µl of an internal standard solution containing odd-chain sphingolipids, were added to each sample. After 10 seconds vortexing, 200 µl of methanol (VWR; PN 34966-2.5L) were added to each Eppendorf tube. Samples were then vortexed for 30 seconds and allowed to equilibrate for 15 minutes at room temperature. Afterwards, samples were centrifuged at 12000 *g* for 15 minutes at 4°C (Eppendorf centrifuge; model 5430R). Then, 80 µl of the supernatant were aliquoted into an LC-MS vial with pre-slit caps (Waters; PN 600000669CV) equipped with a 150 µl inserts (Waters; PN WAT094171) and 7.5 µl of sample were injected. A pooled sample from all materials was used as a quality control of injection (QCinj) and extraction (QCext).

Chromatographic separation was carried out on an ACQUITY UPLC System with a sample manager cooled to 8°C (both from Waters Corporation, Milford, MA, USA). Sphingolipids were separated on a Zorbax Rapid Resolution RRHD C18 Column, 80Å, 1.8 µm, 2.1 mm X 100 mm (Agilent Technologies; Product Number: 758700-902) using a guard column (Agilent Technologies, PN: 821725-901) (5 × 2 mm, 1.8 μm particle size). Mobiles phases A and B consisted of 5 mM ammonium formate (Sigma; PN: 70221) / 0.2% formic acid (Optima, Thermo Fisher Scientific, PN: 10596814) in water and in methanol (VWR, PN: 34966), respectively. Separation was carried out at a 450 μl/min flowrate and a column temperature was held at 40°C. The following chromatographic gradient was used: 0 min, 75% B; time range 0 → 1 min, 75% B (constant); time range 1 → 5 min, 85 → 100% B (linear increase); time range 5 to 15.2 min, 100% B (isocratic range); time range 15.2 → 15.3 min, 100 → 75% B (linear decrease); time range 15.3 → 16 min, 75% B (isocratic column conditioning). Samples were then analysed on a Waters Xevo® TQ-S system equipped with an Electrospray Ion Source (ESI) and ScanWave™ collision cell technology operating in the positive mode. A class specific single reaction monitoring (SRM) transition for each sphingolipid and internal standard was used. The method does not distinguish glycosylated species (GlcCer) from galactosylated species (GalCer), and Glc sphingolipids are therefore potentially a mixture of the two species.

***Quantifying sphingolipids in adipose tissue***

To quantify sphingolipids in adipose tissue, around 25 mg of tissue sample was cut with a stainless-steel blade scalpel. Twenty volumes (*i.e.,* 1 mg = 20 µl) of ice-cold LC-MS grade methanol (Optima, Thermo Fisher Scientific, PN: 10767665) was added to each sample followed by one spoon (spoon provided by manufacturer) of 0.5 mm zirconium oxide beads (Techtum Lab AB, PN Zr0505). Samples were then vortexed for 10 seconds prior to homogenization by a bullet blender (Next Advance) for three cycles of 2 minutes each at a frequency of 8 units. Samples were then further vortexed for 30 seconds and centrifuged at 12000 *g* for 15 minutes at 4°C. Supernatant was subsequently removed and transferred to a 0.1 µm Ultra Centrifugal Spin Filter tube (Millipore; PN UFC30VV00). The samples were then further centrifuged at 8,000 *g* for 4 minutes at 4°C. A volume of 80 µl of the supernatant were aliquoted into an LC-MS vial with pre-slit caps (Waters; PN 600000669CV) equipped with a 150 µl inserts (Waters; PN WAT094171) and 7.5 µl of sample were injected. Chromatographic and mass spectrometric conditions were as reported for the adipose tissue secreted sphingolipids.

***Quantifying sphingolipids in plasma***

To quantify sphingolipids in plasma, samples were divided in batches of 27 samples, 2 QCext and 1 blank of extraction. For each batch, samples were thawed at 4°C and vortexed for 20 seconds. Afterwards, a volume of 25 µl of each sample was aliquoted on an Eppendorf tubes. Then, 10 µl of the internal standard mixture, containing deuterium labelled sphingolipids (at least one per class) was added to each sample. Tubes were then caped, vortexed for 10 seconds and allowed to equilibrate with the internal standard for 10 minutes at 20°C. Then, a volume of 250 µl of methanol was added to each sample. Eppendorf tubes were then closed and vortexed for 10 seconds. Afterwards, samples were sonicated for 15 minutes on an ice bath to avoid the temperature increasing above 20°C. Samples were then centrifuged at 12000 *g* for 15 minutes. An aliquot of 80 µl were finally transferred to two LC-MS vials equipped with a 150 µl insert. To prepare the QCinj, 20 µl of each sample was pooled into a 4 mL glass vial. All samples and QCs were stored at -20°C until the day of analysis. Chromatographic and mass spectrometric conditions were as reported for the adipose tissue secreted sphingolipids.

***RNA Isolation and Quantitative Real Time-Polymerase Chain Reaction (qRT-PCR)***

***RNA isolation***

Total RNA was isolated by a phenol to chloroform (1:5 ratio) separation protocol followed by a magnetic beads-based RNA purification method on a King Fisher magnetic particle processor (Thermo Fisher Scientific), using the MagMAX mirVana total RNA isolation kit (Thermo Fisher Scientific, Catalogue Number A27828). RNA concentration was assessed spectrophotometrically on NanoDrop ND-1000 as recently described (4).

***Reverse transcription***

RNA was reverse-transcribed to cDNA by using the SuperScript VILO mastermix (Thermo Fisher Scientific) following the manufacturer’s instructions and extending the cDNA synthesis step to two hours at 60^o^C on a Veriti thermal cycler (ABI).

***Quantitative real-time PCR***

Quantitative real-time PCR was performed by TaqMan chemistry, using the standard universal TaqMan protocol as indicated by the Manufacturer, on a QuantStudio 7 flex real-time PCR system (Thermo Fisher Scientific). All samples were run in duplicates using 5 ng of cDNA as starting mass, and data was analysed by the Pfaffl method (5). *PPIA* was used as a housekeeping gene for human adipose tissue. The IDs of the TaqMan probes used are: *ASAH1*: Hs00602774_m1; *CERS6*: Hs00826756_m1; *SPTLC1*: Hs00272311_m1, *CERS5*: Hs00332291_m1, *SMPD1*: Hs00609415_m1, *CERK*: Hs00368483_m1; *SPHK2*: Hs00219999_m1.

***Cohort-wide gene expression analyses***

Target gene and housekeeping gene Ct values were quantified by qRT-PCR. Furthermore, the same standards were run on every qPCR plate to extrapolate the amplification efficiency per plate. Target gene and housekeeping gene Ct values were quantified and used to quantify target copy numbers with endogenous housekeeping correction (Efficiency^–ΔCt^). This corrected value was then normalised to the highest standard of the standard curve, providing a corrected expression value relative to the highest standard (Efficiency^–ΔΔCt^). Hence the provided values are housekeeping-normalised expressions relative to a single reference sample (the highest standard of the standard curve), allowing for accurate interpolate analysis.

***Vasomotor studies in human vessels***

Vascular segments obtained during CABG as we have previously described (4) were equilibrated in Radnotti organ bath chambers for 30 minutes, in oxygenated (95% O2/5% CO2) Krebs-Hensleit buffer (NaCl 120mM, KCl 4.7mM, CaCl_2_ 2.5mM, MgSO_4_ 1.2mM, KH2PO4 1.2mM, NaHCO_3_ 25mM, glucose 5.5mM, pH=7.4) at 37°C to achieve a resting tension of 3g. Contractile responses were tested by exposure to Krebs-Hensleit buffer. Four rings from each vessel were pre-contracted with phenylephrine (3x10^-6^M); then endothelium-dependent relaxations were assessed using acetylcholine (ACh, 10^-9^M to 10^-6^M). Finally, relaxations to the endothelium-independent sodium nitroprusside (SNP, 10^-10^M to 10^-7^M) were evaluated.

***Superoxide (O_2_^.-^ ) production measurement***

***Vascular O_2_^.-^*:** O_2_^.-^ production in fresh intact vessels was routinely measured using a lucigenin (5 μmol/L)-enhanced chemiluminescence as described previously (4,6,7). Theatre vessels were incubated in oxygenated (95% O_2_/5% CO_2_) Krebs-HEPES buffer (pH=7.4) for 20 minutes at 37°C to equilibrate. The amount of O_2_^.-^ that were generated by the inner endothelial surface of the vessels were measured before and after addition of NADPH 100 μmol/L to assess the activity of NADPH-oxidases as we have previously described (6).

The N(G)-Nitro-L-arginine methyl ester (L-NAME) inhibitable O_2_^.-^ was assessed by quantifying the difference in arterial and cellular O_2_^.-^ generation before and after addition of L-NAME (Sigma, 1mM), as we have described in the past (7).

***Cellular O_2_^.-^*:** Primary and immortalised human aortic endothelial cells (HAEC) were incubated with C16:0-ceramide (20 nM), or C12:0-ceramide (20 nM) for 20 min, then scraped in ice-cold buffer (KH_2_PO_4_ 50mM, EGTA 1 mM, sucrose 150 mM, pH=7) in the presence of a protease inhibitor cocktail (Roche Applied Science, Indianapolis, IN) and lysed using an ultrasound bath. The production of O_2_^.-^ was quantified in the presence and the absence of L-NAME (1mM). Production of O_2_^.-^ was measured using a lucigenin (5 μmol/L)-enhanced chemiluminescence assay as we have previously described (6). The difference in intracellular O_2_^.-^ generation before and after addition of L-NAME was calculated to assess the coupling status of endothelial nitric oxide synthase (eNOS).

***Primary adipocyte isolation and differentiation***

Thoracic adipose tissue was cut in small pieces and subjected to collagenase (1 mg/ml) digestion at 37°C for 45 minutes in PBS buffer, then centrifuged at 150×g for 5 minutes at room temperature. Cell pellets were washed once with PBS and re-suspended in DMEM/F12 (Sigma, D6421) containing 10% FBS (Sigma, F9665), 1% glutamine (Invitrogen, 15140-122), 1% penicillin-streptomycin (Invitrogen, 25030-024) and 1% of 0.5 ng/ml FGF, 1 ng/ml EGF (Sigma) and. For differentiation assays, confluent pre-adipocytes were stimulated with DMEM/F12 culture medium containing 100units/ml penicillin-streptomycin supplemented with 45mM isobutylmethylxanthine (Sigma, I7018), 17μM pantothenate (Sigma, P5710), 820μM Biotin (Sigma, B4639), 4.14μm dexamethasone (Sigma, D2915), 688μm insulin (Invitrogen,12585-014), 1.5mM triiodothyronine (Sigma, T5516), and 4.5mM troglitazone (Sigma, T2573) for 4 days then cultured in same medium but lacking isobutylmethylxanthine and troglitazone for 10 more days.

***Endothelial cells culture and treatment***

Immortalized human aortic endothelial cells (teloHAEC) (ATCC, CRL-4052) and Primary HAEC from different donors (Lonza Bioscience, CC-2535) were grown in EBM-2 basal medium (Lonza Bioscience, CC-3156) and EGM-2 SingleQuots Supplements (Lonza Bioscience, CC-4176). At 70-80% confluency cells were subcultured in 6 well plates and treated for 20 minutes with 20 μM C16:0-ceramide (Avanti Polar Lipids, 860516P), 20 μM C12:0-ceramide, 20μM C6:0-ceramide, < 0.1% Dimethyl sulfoxide (DMSO, Sigma), 5 μM NBD C6-Ceramide (Thermo Fisher Scientific, N22651), 10 μM LB100 (Cambridge Bioscience, B2031-5), 10 μM Dihydroethidium (DHE; Thermo Fisher Scientific, D11347) staining following the manufacturers protocols.

***Isolation and characterisation of adipocyte-derived extracellular vesicles***

Primary adipocytes were cultured in T175cm^2^ flasks in 15ml media without fetal bovine serum (FBS). Cells were grown to 80-90% confluence and cell culture supernatants harvested, centrifuged for 10 minutes at 1000*g* to pellet cells and cellular debris. Subsequently, cell culture supernatants were transferred to polyallomer Quick-Seal ultra-clear 16 mm × 76 mm tubes, Beckman Coulter) ultracentrifugation tubes and centrifuged for 2 hours at 120,000g. Pelleted EV were washed by re-suspending adipocyte EV pellets in 13mL sterile phosphate buffered saline (PBS) and centrifuged 120,000g for 1 hour. Isolated and washed adipocyte EV were characterised as detailed below in agreement with guidelines published by the International Society for Extracellular Vesicles (ISEV) (8). Media controls consisted of cell culture media that had not been exposed to cells were treated in the same way and served as negative controls.

Adipocyte size and concentration profiles (Figure 4E-F; Online-Figure 17) were determined on a ZetaView (Particle Metrix, Germany) fitted with a 40mW and 488nm laser by diluting samples 1:1000 in PBS to a final volume of 1mL using the following settings for each measurement: 11 scanning cell positions, 30 frames per position, autofocus, camera sensitivity 65 and shutter 100. Measurements were analysed on the ZetaView software.

Adipocyte EV were immunoblotted for EV markers ALIX, TSG101 and CD9 (Figure 4E-F, Online-Figure 19) and markers of cellular contamination by probing for ATP5A (mitochondria), histone H3 (nuclear fragments) and PERK (endoplasmic reticulum) as previously described (9). Briefly, washed adipocyte EV were lysed in 1x RIPA buffer with protease and phosphatase inhibitors, followed by needle sonication for 10s. Adipocyte EV were separated on Bis-Tris 4-12% gradient gels using LSD-loading buffer under non-reducing conditions. Separated proteins were transferred to nitrocellulose membranes and blocked using 5% milk in PBS-tween (1%) at room temperature for 1 hour. The membranes were cut at respective molecular weights and probed using primary antibodies overnight at 4^o^C anti- ALG-2-interacting protein X (ALIX), (ab117600, Abcam) (1/2000 dilution), anti-ATP5A (15H4C4, Abcam) (1/5000), anti-histone H3 (D1H2/4499P, Cell signalling) (1/2000), anti- protein kinase-like endoplasmic reticulum kinase (PERK) (C33E10 Cell Signalling) (1/1000), CD9 (EXOAB-KIT-1, System Biosciences) (1/1000), TSG101 (MABC786, clone 4A10, Millipore) (1:1000) in 5% milk in PBS-tween (1%). Membranes were washed and incubated with respective secondary antibodies conjugated to horseradish peroxidase. Membranes were washed again before incubation with enhanced chemiluminescence substrate (Pierce ECL, Thermo Fisher Scientific) and imaged on a Bio-Rad ChemiDoc MP Imaging system.

Adipocyte EV morphology was confirmed by transmission electron microscopy using the electron microscopy facility at the Dunn School, Central Oxford Structural Molecular Imaging Center (COSMIC), University of Oxford. For negative staining, grids (300 mesh Cu carbon film) were glow discharged for 20seconds at 15 mA (Leica EM ACE 200). 20 µL of adipocyte EV were applied to the grid for 2 minutes, blotted, stained with 2% uranyl acetate for 20s, blotted and allowed to air dry. Images were acquired on a 120kV Tecnai 12 (Thermo Fisher Scientific) TEM equipped with a OneView digital camera (Gatan).

***Isolation and characterisation of circulating extracellular vesicles***

Plasma extracellular vesicles were isolated by differential centrifugation as previously described (9). In brief, 200uL of plasma was defrosted at 4^o^C and centrifuged for 10 minutes at 5000 x g. The supernatant was transferred to ultracentrifuge tubes (Polyallomer Quick-Seal ultra-clear 16 mm × 76 mm tubes, Beckman Coulter) and centrifuged at 120,000 g for 120 minutes to isolate all small EVs. Isolated vesicles were re-suspended in 100 μL PBS and utilized in studies as described. Plasma EV size and concentration was determined Nanoparticle Tracking Analysis (Online-Figure 20).

***Quantifying sphingolipids in extracellular vesicles***

To quantify sphingolipids in adipose tissue- and plasma-derived extracellular vesicles (10), EV suspensions in PBS were thawed at 4°C. Afterwards, 25 µl of each sample were transferred to an Eppendorf tube. Then, 10 µl of the solution containing labelled internal standards was added to each sample. Samples were then vortexed and allowed to equilibrate at room temperature (22 ⁰C). This was followed by the addition of 100 µl of methanol to each sample. Samples were then vortexed for 30 seconds and sonicated for 15 minutes in an ultrasound bath (Sweep function) with ice. Next, samples were centrifuged at 12000 *g* during 15 minutes at 6⁰C. Finally 100 µl of the supernatant were transferred to an LC-MS amber vial equipped with a 150 µl insert. Chromatographic and mass spectrometric conditions were as reported for the adipose tissue secreted sphingolipids.

***Western blot analysis***

Cells were harvested and lysed in radioimmunoprecipitation assay (11) buffer, protein quantified by the Bicinchoninic acid assay (BCA), 20µg of protein extracts were analysed by SDS-PAGE on 4-12% polyacrylamide gradient gel and transferred to nitrocellulose membranes (Amersham, UK). Then the proteins were blotted with antibodies for human eNOS and phosphorylated eNOS-Ser1177 and -Tyr495 (BD Bioscience, 1:1000), AKT and phosphorylated AKT-Ser473 (Cell Signalling Technology, UK), PP2A and phosphorylated PP2A-Tyr307 (Cell Signalling Technology, UK). Horseradish peroxidase (HRP)-conjugated anti-rabbit (A9169, Sigma, 1:10,000) and HRP-conjugated anti-mouse (A9044, Sigma, 1:15,000) were used as secondary antibodies as appropriate.

For detection of immuno-reactive bands, ECL select Western Blotting Detection Reagent (Amersham, UK) was used. Western blots were quantified using Image Lab Bio-rad software integrated density analysis.

***Biopterins Quantification by HPLC***

Cell pellets were freeze-thawed in ice-cold resuspension buffer (50 mM), pH 7.4, containing dithioerythritol (1 mM) and EDTA (100 μM) and subjected to three free-thaw cycles. After centrifugation (15 min at 13,000 rpm and 4 °C), samples were transferred to new, cooled micro tubes and precipitated with cold phosphoric acid (1 M), trichloroacetic acid (2 M) and dithioerythritol (1 mM). Samples were vigorously mixed and then centrifuged for 15 min at 13,000 rpm and 4 °C. Samples were injected onto an isocratic HPLC system and quantified using sequential electrochemical (Coulochem III, ESA, Inc.) and fluorescence (Jasco) detection. HPLC separation was performed using a 250-mm ACE C-18 column (Hichrom) and a mobile phase comprised of sodium acetate (50 mM), citric acid (5 mM), EDTA (48 μM), and dithioerythritol (160 μM) (pH 5.2) (all ultrapure electrochemical HPLC grade) at a flow rate of 1.3 ml/min. Background currents of +500 μA and −50 μA were used for the detection of BH4 on electrochemical cells E1 and E2, respectively. 7,8-BH2 and biopterin were measured using a Jasco FP2020 fluorescence detector. Quantification of BH4, BH2, and B was done by comparison with authentic external standards and normalized to protein concentration, determined by the BCA assay.

***Computerized tomography (CT) imaging***

Participants in Study Arm 1 underwent a non-contrast CT scan on a 64-slice scanner (LightSpeed Ultra, General Electric). A non-contrast prospectively ECG triggered axial acquisition CT scan was obtained (0.35 sec rotation time, 2.5 mm axial slice thickness, 20mm detector coverage, tube energy of 120 kV and 200 mA) with the carina and the diaphragm used as cranial and caudal landmarks respectively. Lung field of view was extended to cover the entire thoracic soft tissue. The reconstructed images were transferred to an offline image processing and analysis workstation (Aquarius Workstation V.4.4.11 TeraRecon). Adipose tissue was defined as all voxels with attenuation between -190 and -30 Hounsfield Units (HU), as previously described (12). ThAT included all intrathoracic fat located between the pulmonary artery bifurcation and the diaphragm, and was estimated as a surrogate marker of visceral obesity. ScAT was tracked by sampling of all thoracic subcutaneous AT, at the height of the caudal end of sternum and extending cranially for a total of 25 mm, as previously described (12).

***Extraction and definition of endpoints***

Mortality outcome data in the Ox-HVF cohort were collected by linking the Office for National Statistics (ONS) data with the National Health Service (NHS) Digital databases in England and Wales. Patients had previously provided consent, and the collected data was first stored in a secured network and subsequently link-anonymized and analysed. All patients were followed up from the day of surgery until the date of NHS Digital data retrieval (28 March 2017). All causes of death in the Ox-HVF cohort and tested in this study are listed in Online-Table 8.

**-ONLINE RESULTS-**

**Association of fat volume with vascular superoxide**

Further to the age- and sex- adjusted association between ThAT (but not ScAT) volume and superoxide generation in the internal mammary arteries (IMA), we have also explored the same association for superoxide generation in the saphenous veins (SV). No significant association was detected for ThAT (Standardized Beta[95% CI]= -0.016[-0.004-0.003]; *p*=0.87) or ScAT (Standardized Beta[95% CI]= 0.123[-0.001- 0.004]; *p*=0.367), after correction for age and sex.

***CERK* expression is reduced in ThAT obtained from obese patients**

We measured the expression of key enzymes involved in ceramides biosynthesis in ThAT biopsies obtained from 427 patient of the Ox-HVF cohort. The tested enzymes include Ceramide synthases (*CERC5*, *CERC6*) that are known to synthesise Cer16:0, Serine palmitoyltransferase (*SPTLC1*) the rate-determining enzyme in SPL *denovo* synthesis, Acid ceramidase (*ASAH1*), Sphingosine kinase (*SPHK2*), Acid sphingomyelinase (*SMPD1*), and Ceramide kinase (*CERK*). Among all investigated enzymes, a significant reduction of *CERK* expression was observed in ThAT biopsies obtained from obese patients with BMI ≥ 35 Kg/m^2^ (n=22) as compared to its expression in ThAT biopsies obtained from patients with BMI Kg/m^2^ < 35 (n=247) (as shown in Online-Figure 5). CERK enzyme catalyses the conversion of ceramides to ceramide 1-phosphate, a sphingolipid metabolite that reduces pro-apoptotic ceramides and enhances pro-survival signalling.

**Cer16:0 in circulation is bound in extracellular vesicles**

We isolated extracellular vesicles (EV) from plasma samples of 15 patients and quantified the amount of EV-bound sphingolipids (SPL) as well as the amount of SPL in the corresponding plasma samples. As illustrated in Online-Figure 11, Cer16:0 and all its derivatives are detectable in EV derived from plasma. In plasma and circulating EV, ceramides with longer acyl chains (>C20:0) represent the major fraction of all determined ceramides (Online-Figure 11A). Whereas, Cer16:0 derivatives including C16:0-Glucosylceramide (Online-Figure 11B), C16:0-sphingomyelin (Online-Figure 11C), C16:0-Lactosylceramide (Online-Figure 11D) were the dominant species relative to their classes. We observed that, within the ceramides carried by the EV fraction, Cer16:0 contribution was largely increased (two times), relative to their total proportion in circulation (Online-Figure 11A). Longer chain ceramides proportions were lower on the contrary, except for Cer24:1 (Online-Figure 11A). Indeed, all C16:0 species were the major components of circulating EV (Online-Figure 11B-D).

**Cer16:0, GlcCer16:0, Cer16:0/Cer22:0 and Cer16:0/Cer24:0 associated with worse cardiac outcomes.**

We tested the association between Cer16:0, GlcCer16:0 and Cer16:0 ratios tested in this manuscript with cardiovascular (CV) death (n=20) and a composite endpoint including cardiovascular death and/or hospital admission for heart failure (n=29). All the tested metabolites were found to be associated with CV death (Online-Table 9), worse cardiac outcomes (Online-Figure 17) and endpoints (Online-Table 10).

-**ONLINE TABLES**-

**Online-Table 1.** Key resources

| REAGENT or RESOURCE | SOURCE | IDENTIFIER |
| --- | --- | --- |
| Antibodies | | |
| Purified Mouse Anti-eNOS (pS1177) | BD Biosciences | 612393 |
| Purified Mouse Anti-eNOS (pT495) | BD Biosciences | 612706 |
| Purified Mouse Anti-eNOS | BD Biosciences | 610297 |
| Purified Mouse Anti-AKT | BD Biosciences | 610860 |
| Purified Mouse Anti-Akt (pS472/pS473) | BD Biosciences | 550747 |
| PP2A C Subunit (52F8) Rabbit mAb | Cell Signaling Technology | 2259T |
| Phospho-PP2A (Y307) Catalytic Subunit Affinity Purified PAb | R&D Systems | AF3989-SP |
| HRP-conjugated anti-rabbit | Sigma-Aldrich | A9169 |
| HRP-conjugated anti-mouse | Sigma-Aldrich | A9044 |
| Anti-TSG101, clone 4A10 Antibody | Millipore | MABC786 |
| ExoAb Antibody Kit (CD9 rabbit anti-human) with goat anti-rabbit HRP secondary antibody | System Biosciences | EXOAB-KIT-1 |
| Chemicals, Peptides, and Recombinant Proteins | | |
| Protease Inhibitor Cocktail (100X) | Cell Signaling Technology | 5871S |
| Phosphatase Inhibitor Cocktail (100X) | Cell Signaling Technology | 5870S |
| EGM-2 Endothelial Medium BulletKit | Lonza Biologics PLC | CC-3162 |
| EGM-2 MV Endothelial Med BulletKit | Lonza Biologics PLC | CC-3202 |
| DULBECCOS MODIFIED EAGLES MEDIUM/NUTRI | Sigma-Aldrich | D6421 |
| FETAL BOVINE SERUM HEAT INACTIVATED | Sigma-Aldrich | F9665 |
| Accutase® Cell Detachment Solution | BioLegend | 423201 |
| NuPAGE Novex 4-12% Bis-Tris Protein Gels, 1.0 mm, | Life Technologies | NP0321 |
| NuPAGE MOPS SDS Running Buffer (20X)-500 mL | Life Technologies | NP0001 |
| NuPAGE Transfer Buffer (20X)-1 L | Life Technologies | NP00061 |
| D-Pantothenic acid hemicalcium salt | Sigma-Aldrich | P5710  CAS Number: 137-08-6 |
| 3,3′,5-Triiodo-L-thyronine sodium salt | Sigma-Aldrich | T5516  CAS Number: 55-06-1 |
| Biotin | Sigma-Aldrich | B4639  CAS Number: 58-85-5 |
| Dexamethasone-Water Soluble | Sigma-Aldrich | D2915  CAS Number: 50-02-2 |
| 3-Isobutyl-1-methylxanthine | Sigma-Aldrich | I7018  CAS Number: 28822-58-4 |
| Troglitazone | Sigma-Aldrich | T2573 |
| Insulin, human recombinant, zinc solution-5 mL | Life Technologies | 12585-014 |
| Collagenase. Type II. C. histolyticum | Sigma-Aldrich |  |
| β-Nicotinamide adenine dinucleotide 2′-phosphate reduced tetrasodium salt hydrate | Sigma-Aldrich | N1630  CAS Number: 2646-71-1 (anhydrous) |
| RIPA Buffer (10X) | Sigma-Aldrich |  |
| Dulbecco’s Modified Eagle’s Medium/Nutrient Mixture F-12 Ham | Sigma-Aldrich | D6421 |
| Dihydroethidium (Hydroethidine) | Life Technologies | D11347 |
| PBS, pH 7.4-10 x 500 mL | Life Technologies | 10010056 |
| Trypsin-EDTA (0.25%), phenol red-100 mL | Life Technologies | 25200056 |
| X10 16% FORMALDEHYDE (W/V), METHANOL-FREE 10ML | Life Technologies | 11586711 |
| NBD C6-Ceramide Complexed to BSA-5 mg | Life Technologies | N22651 |
| Nω-Nitro-L-arginine methyl ester hydrochloride | Sigma-Aldrich | N5751  CAS Number 51298-62-5 |
| LB100 | Cambridge Bioscience | B2031-5 |
| Methanol Optima^TM^ LC-MS | Fisher Chemicals | A454-212 |
| Methanol Chromasolv^TM^ LC-MS | Honeywell | 34966 |
| Acetonitrile Optima^TM^ LC-MS | Fisher Chemicals | A955-212 |
| Isopropanol Optima^TM^ LC-MS | Fisher Chemicals | A461-212 |
| Formic acid Optima^TM^ 99%LC-MS | Fisher Chemicals | A117-50 |
| Ammonium Formate LC-MS >99% | Sigma-Aldrich | 70221 |
| Sphingomyelin d18:1/C12:0 | Avanti Lipids | 860583P |
| Sphingomyelin d18:1/C16:0 | Avanti Lipids | 860584P |
| Sphingomyelin d18:1/C17:0 | Avanti Lipids | 860585P |
| Sphingomyelin d18:1/C18:0 | Avanti Lipids | 860586P |
| Sphingomyelin d18:1/C18:1 | Avanti Lipids | 860587P |
| Sphingomyelin d18:1/C24:0 | Avanti Lipids | 860592P |
| Sphingomyelin d18:1/C24:1 | Avanti Lipids | 860593P |
| Ceramide d18:1/C6:0 | Avanti Lipids | 860506P |
| Ceramide d18:1/C12:0 | Avanti Lipids | 860512P |
| Ceramide d18:1/C14:0 | Avanti Lipids | 860514P |
| Ceramide d18:1/C16:0 | Avanti Lipids | 860516P |
| Ceramide d18:1/C16:0 | Avanti Lipids | 860517P |
| Ceramide d18:1/C18:0 | Avanti Lipids | 860518P |
| Ceramide d18:1/C18:1 | Avanti Lipids | 860519P |
| Ceramide d18:1/C20:0 | Avanti Lipids | 860520P |
| Ceramide d18:1/C22:0 | Avanti Lipids | 860501P |
| Ceramide d18:1/C24:0 | Avanti Lipids | 860524P |
| Ceramide d17:1/C24:1 | Avanti Lipids | 860650P |
| Ceramide d18:1/C24:1 | Avanti Lipids | 860525P |
| Dihydroceramide d18:0/C16:0 | Avanti Lipids | 860634P |
| Dihydroceramide d18:0/C24:0 | Avanti Lipids | 860628P |
| Glucosylceramide d18:1/C12:0 | Avanti Lipids | 860543P |
| Glucosylceramide d18:1/C16:0 | Avanti Lipids | 860539P |
| Glucosylceramide d18:1/C18:0 | Avanti Lipids | 860547P |
| Glucosylceramide d18:1/C18:1 | Avanti Lipids | 860548P |
| Glucosylceramide d18:1/C24:1 | Avanti Lipids | 860549P |
| Lactosylceramide d18:1/C12:0 | Avanti Lipids | 860545P |
| Lactosylceramide d18:1/C16:0 | Avanti Lipids | 860576P |
| Lactosylceramide d18:1/C17:0 | Larodan | 56-1052 |
| Lactosylceramide d18:1/C24:0 | Avanti Lipids | 860577P |
| Lactosylceramide d18:1/C24:1 | Avanti Lipids | 860597P |
| d18:1-Sphingosine | Avanti Lipids | 860490P |
| d18:0-Sphinganine | Avanti Lipids | 860498P |
| d18:1-Sphingosine 1 Phosphate | Avanti Lipids | 860492P |
| d18:0-Sphinganine 1 Phosphate | Avanti Lipids | 860536P |
| Ceramide-1-Phosphate d18:1/C16:0 | Avanti Lipids | 860533P |
| Ceramide-1-Phosphate d18:1/C24:0 | Avanti Lipids | 860527P |
| d7-Ceramide d18:1/C16:0 | Avanti Lipids | 860676P |
| d7-Ceramide d18:1/C18:0 | Avanti Lipids | 860677P |
| d7-Ceramide d18:1/C24:1 | Avanti Lipids | 860679P |
| d9-Sphingomyelin d18:1/C18:1 | Avanti Lipids | 791649C |
| d18:1-Sphingosine | Avanti Lipids | 860657P |
| d7-d18:1-Sphingosine 1 Phosphate | Avanti Lipids | 860659P |
| Glucosylceramide d18:1/C16:0 | Matreya LLC | 1533 |
| Glucosylceramide d18:1/C18:0 | Avanti Lipids | 860638P |
| Lactosylceramide d18:1/C16:0 | Matreya LLC | 1534 |
| Experimental Models: Cell Lines | | |
| HAEC (Human Aortic Artery Endothelial cells) | Lonza Biologics PLC | CC-2535 |
| TeloHAEC (Homo sapiens aorta normal) | American Type Culture Collection Co. (ATCC) | ATCC CRL-4052 |
| Human primary pre-adipocytes | John Radcliffe Hospital Labs | N/A |
| Primary adipose tissues | John Radcliffe Hospital Labs | N/A |
| Software and Algorithms | | |
| MetaboAnalyst (version 4.0) | Chong, 2019 | https://www.metaboanalyst.ca/ |
| SPSS Statistics (version 25) | IBM | https://www.ibm.com/uk-en/analytics/spss-statistics-software |
| Prism (version 8.0) | GraphPad | <https://www.graphpad.com/scientific-software/prism/> |
| R (version 3.5) | The R Project for Statistical Computing | <https://www.r-project.org/> |
| Matlab (version R2015a) | Mathworks | <https://mathworks.com/products/matlab.html> |
| MassHunter Profinder (version B.06.00) | Agilent Technologies | N/A |
| Masslynx (version v4.1) | Waters Corporation | N/A |
| Targetlynx (version v4.1) | Waters Corporation | N/A |
| Other | | |
| Agilent 1290 Infinity II LC system | Agilent Technologies | N/A |
| Agilent iFunnel 6550 QTOF mass spectrometer | Agilent Technologies | N/A |
| Waters Acquity UPLC system | Waters | N/A |
| Waters Xevo TQ-S mass spectrometer | Waters | N/A |
| SeQuant® ZIC®-HILIC (100 mm × 2.1 mm, 3.5 µm) | Merck-Millipore | 1.50441.0001 |
| SeQuant® ZIC®-HILIC guard column (20 mm × 2.1 mm, 3.5 µm) | Merck-Millipore | 1.50436.0001 |
| Zorbax Eclipse Plus C18, RRHD (100 mm × 2.1 mm, 1.8 µm) | Agilent | 959758-902 |
| Zorbax Eclipse Plus C18 guard column (5 mm × 2.1 mm, 1.8 µm) | Agilent | 821725-901 |
| Amber LC-MS vials with 300 µl inserts | Agilent | 5188-6592 |
| Amber LC-MS vials with pre-slit caps | Waters | 600000669CV |
| LC-MS vial 150 µl inserts | Waters | WAT094171 |
| Ultrafree-MC Centrifugal Filter 0.1 µm PVDF | Merck-Millipore | UFC30VV00 |
| Zirconium oxide beads 0.5 mm | Techtum Lab | Zr0505 |
| Deposited Data |  |  |
| https://data.mendeley.com/datasets/jpwrmnkxn4/draft?a=3ea97781-f2f3-47f9-b73f-2364751b3061 | | |

| **Online-Table 2.** Baseline characteristics of the study participants. | | | | |  |
| --- | --- | --- | --- | --- | --- |
| **Characteristics** | **Study 1** | **Study 2** | | **Study 3** | |
|  |  | **Lean** | **Obese** |  | |
| Patients # | 633 | 17 | 31 | 32 | |
| Age (years) | 66.6±9.7 | 63.4±8.4 | 62.1±11.9 | 51.4±9.9 | |
| Male (%) | 85.8 | 76.4 | 80.6 | 0.125 | |
| BMI (kg/m2) | 28.7±4.6 | 23.7±1.2 | 32.7±4.1*** | 33.5±2.5 | |
| Hypertension (%) | 75.4 | 82.4 | 90.3 | 9.4 | |
| Hyperlipidaemia (%) | 82.6 | 76.5 | 64.5 | - | |
| T2DM (%) | 22.7 | 47 | 32.3 | - | |
| Smoking (%) | 73.5 | 58.8 | 64.5 | 65.6 | |
| Cholesterol (mmol/L) | 3.4±1.0 | 3.1±0.7 | 3.8±1.1 | 4.9±0.9 | |
| Trigylecerides (mmol/L) | 1.4±0.9 | 1.1±0.7 | 1.7±0.8 | 1.1±0.5 | |
| HDL (mmol/L) | 0.9±0.2 | 0.9±0.3 | 0.83±0.2 | 1.4±0.3 | |
| LDL (mmol/L) | 1.9±0.8 | 1.6±0.6 | 2.1±0.9 | 3.0±0.8 | |
| Antiplatelet (%) | 85 | 88.2 | 77.4 | - | |
| βblocker (%) | 70.6 | 82.4 | 64.5 | 9.4 | |
| Statins (%) | 88.3 | 88.2 | 83.9 | - | |
| ACEi (%) | 51 | 58.8 | 61.3 | 9.4 | |
| Insulin (%) | 7.74 | 5.9 | 3.2 | - | |
| Any Oral hypoglycemic | 16.3 | 33.3 | 33.3 | - | |

ACEi, Angiotensin converting enzyme inhibitor; BMI, Body mass index; T2DM, Type 2 diabetes mellitus; Continuous variables are presented as mean±SD; ****P<0.001* Lean vs obese by Chi-square tests.

**Online-Table 3.** Number of metabolites identified (accurate mass, retention time, MS fragments) for each tissue (Study 2) on each mode and their quality control value.

| **Platform** | | **ScAT** | | | **ThAT** | | | |
| --- | --- | --- | --- | --- | --- | --- | --- | --- |
|  |  | ***n*** | **%CV^a^** | | | ***n*** | | **%CV^a^** |
| HILIC+ | 46 | | | 2.9 [0.9-16.0] | 43 | | 1.7 [0.7-7.8] | |
| HILIC- | 11 | | | 2.8 [2.9-18.4] | 11 | | 4.6 [2.5-13.8] | |
| RP+ | 41 | | | 4.3 [2.4-22.1] | 43 | | 4.3 [0.6-23.0] | |
| RP- | 18 | | | 6.4 [0.8-15.5] | 18 | | 4.6[3.1-16.0] | |

^a^ Median [25^th^-27^th^]

HILIC, Polar metabolites; RP, Non-polar metabolites; ScAT, Subcutaneous adipose tissue; ThAT, Thoracic adipose tissue. The analysis was performed in 48 patients.

|  | | |
| --- | --- | --- |
| **Online-Table 4.** Differential metabolites in ThAT secretome versus ScAT secretome in Study 2. | | |
| **Metabolites** | ***P*.value** | **FDR-adjusted *P*.value** |
| Arginine | 7.11E-15 | 5.26E-14 |
| Asparagine | 7.11E-15 | 5.26E-14 |
| Aspartate | 7.11E-15 | 5.26E-14 |
| Cytosine | 7.11E-15 | 5.26E-14 |
| Glucose | 7.11E-15 | 5.26E-14 |
| Lysine | 7.11E-15 | 5.26E-14 |
| Phenylalanine | 7.11E-15 | 5.26E-14 |
| gamma-Muricholic acid | 7.11E-15 | 5.26E-14 |
| Glycocholic acid | 7.11E-15 | 5.26E-14 |
| Serine | 7.11E-15 | 5.26E-14 |
| Hydroxyproline | 7.11E-15 | 5.26E-14 |
| Alanine | 7.11E-15 | 5.26E-14 |
| alpha-Glutamyl Tyrosine | 7.11E-15 | 5.26E-14 |
| Creatine | 7.11E-15 | 5.26E-14 |
| Leucine | 7.11E-15 | 5.26E-14 |
| Adenine | 1.42E-14 | 9.86E-14 |
| Cysteine | 7.11E-14 | 4.64E-13 |
| Elaidic acid | 3.91E-13 | 2.41E-12 |
| Glutamine | 6.25E-13 | 3.65E-12 |
| Glutamate | 1.20E-12 | 6.35E-12 |
| Deoxycholic acid | 1.20E-12 | 6.35E-12 |
| Valine | 1.47E-12 | 7.42E-12 |
| LysoPC (18:0) | 5.41E-12 | 2.61E-11 |
| LysoPC (16:0) | 7.60E-12 | 3.51E-11 |
| Methionine | 8.96E-12 | 3.98E-11 |
| LysoPAF-C16:0 | 1.05E-11 | 4.50E-11 |
| Uracil | 9.23E-11 | 3.80E-10 |
| Nicotinamide | 1.36E-10 | 5.40E-10 |
| Paraxanthine | 7.23E-10 | 2.77E-09 |
| L-Proline | 1.01E-09 | 3.73E-09 |
| Kynurenic acid | 1.72E-09 | 6.17E-09 |
| Hypoxanthine | 1.92E-08 | 6.67E-08 |
| Cer18:0 | 2.52E-08 | 8.47E-08 |
| D-Malic acid | 3.86E-08 | 1.26E-07 |
| Uridine | 4.56E-08 | 1.42E-07 |
| Kynurenine | 4.61E-08 | 1.42E-07 |
| GlcCer18:0 | 1.19E-07 | 3.56E-07 |
| Inosine | 2.25E-07 | 6.56E-07 |
| Oleic acid | 2.61E-07 | 7.43E-07 |
| Succinic acid | 4.06E-07 | 1.13E-06 |
| Histidine | 7.71E-07 | 2.09E-06 |
| Taurolithocholic acid | 1.02E-06 | 2.68E-06 |
| Palmitic acid | 1.98E-06 | 5.12E-06 |
| Stearic acid | 3.53E-06 | 8.90E-06 |
| Eicosapentaenoyl-PAF-C16:0 | 5.69E-06 | 1.40E-05 |
| Calcitriol | 5.79E-06 | 1.40E-05 |
| Linoleic acid | 1.48E-05 | 3.50E-05 |
| Cer16:0 | 1.75E-05 | 4.06E-05 |
| Cer24:0 | 2.19E-05 | 4.87E-05 |
| Taurine | 2.19E-05 | 4.87E-05 |
| Leucylleucine | 3.97E-05 | 8.64E-05 |
| Deoxyinosine | 4.19E-05 | 8.93E-05 |
| Dihomo-g-Linolenoyl-PAF-C16:0 | 4.65E-05 | 9.73E-05 |
| Cer20:0 | 5.72E-05 | 0.00011753 |
| Xanthine | 0.00013968 | 0.00028189 |
| Cer24:1 | 0.00017706 | 0.00035096 |
| Glutathione reduced | 0.00023396 | 0.0004556 |
| Cer18:1 | 0.00032123 | 0.00061477 |
| Arachidonoyl-PAF-C16:0 | 0.00033588 | 0.00063192 |
| PC (10:0/10:0) | 0.00061614 | 0.0011399 |
| Adenosine | 0.00089486 | 0.0016283 |
| Cer22:0 | 0.0013347 | 0.0023896 |
| Palmitoylcarnitine | 0.0016843 | 0.0029676 |
| Tryptophan | 0.00175 | 0.0030351 |
| Chenodeoxycholic acid | 0.0021113 | 0.0036054 |
| Acetyl-L-Leucine | 0.0028397 | 0.0047045 |
| PC (16:0/16:0) | 0.0028397 | 0.0047045 |
| Oleamide | 0.003399 | 0.0055484 |
| Nicotinic acid | 0.003915 | 0.0062981 |
| Citric acid | 0.0048198 | 0.0076429 |
| Dihomo-g-Linolenic acid | 0.0057778 | 0.0090329 |
| Threonine | 0.0074393 | 0.011469 |
| PC (18:0/14:0) | 0.0081973 | 0.012464 |
| Citrulline | 0.0090222 | 0.013533 |
| Ornithine | 0.0093128 | 0.013783 |
| Glycolithocholic acid | 0.010892 | 0.015908 |
| SM18:0 | 0.013904 | 0.020044 |
| alph-Linolenic acid | 0.015477 | 0.022025 |
| Cer14:0 | 0.025441 | 0.035747 |
| GlcCer16:0 | 0.027618 | 0.03832 |
| Pyroglutamate | 0.033316 | 0.045655 |
| Eicosapentaenoic acid | 0.036189 | 0.048987 |

Cer, ceramide; GlcCer, glycosylceramide; PC, phosphatidylcholine; PAF, Platelet-activating factor; SM, sphingomyelin; ScAT, subcutaneous adipose tissue; ThAT, thoracic adipose tissue. ThAT secretome versus ScAT secretome *P* values were calculated in MetaboAnalyst v4.0 by Wilcoxon signed-rank tests. This analyses were performed in 48 patients from study 2.

| **Online-Table 5.** Concentrations of plasma sphingolipids in Study 1. | | | | |
| --- | --- | --- | --- | --- |
| **Sphingolipid metabolite** | **Concentration**  **(nmol/L)** | **QCinj %CV** | **QCext %CV** | **Internal standard** |
| Sph(d18:1) | 3.756±5.706 | 12.1 | 19.4 | d_7_-Sph(d18:1) |
| Spa(d18:0) | 3.562±2.804 | 21.1 | 14.3 | d_7_-Sph(d18:1) |
| Sph1P(d18:1) | 210.585±58.467 | 3.7 | 6.1 | d_7_-S1P(d18:1) |
| Sph1P(d17:1) | 6.156±1.715 | 10.0 | 11.9 | d_7_-S1P(d18:1) |
| Spa1P(d18:0) | 49.671±15.968 | 4.3 | 5.5 | d_7_-S1P(d18:1) |
| DhCer16:0 | 21.174±13.535 | 6.8 | 7.6 | d_7_-Cer18:0 |
| DhCer24:0 | 344.84±227.57 | 19.1 | 10.5 | d_7_-Cer24:1 |
| Cer12:0 | 1.197±0.839 | 17.8 | 19.6 | d_7_-Cer18:0 |
| Cer14:0 | 22.087±11.171 | 18.9 | 15.9 | d_7_-Cer18:0 |
| Cer16:0 | 162.531±43.456 | 12.9 | 9.7 | d_7_-Cer16:0 |
| Cer17:0 | 5.712±1.954 | 5.1 | 9.1 | d_7_-Cer18:0 |
| Cer18:1 | 2.943±1.557 | 11.2 | 9.5 | d_7_-Cer18:0 |
| Cer18:0 | 147.213±61.673 | 6.2 | 6.9 | d_7_-Cer18:0 |
| Cer20:0 | 225.1±111.426 | 6.8 | 9.6 | d_7_-Cer18:0 |
| Cer22:0 | 725.142±351.745 | 13.5 | 8.8 | d_7_-Cer24:1 |
| Cer24:1 | 974.45±305.651 | 2.3 | 6.2 | d_7_-Cer24:1 |
| Cer24:0 | 2559.889±1200.308 | 17.4 | 10.6 | d_7_-Cer24:1 |
| SM12:0 | 452.48±208.961 | 5.7 | 7.7 | d_9_-SM18:1 |
| SM16:0 | 60968.948±11307.856 | 4.2 | 4.9 | d_9_-SM18:1 |
| SM17:0 | 3487.513±969.823 | 8.6 | 6.9 | d_9_-SM18:1 |
| SM18:1 | 7847.036±2022.156 | 3.1 | 5.7 | d_9_-SM18:1 |
| SM18:0 | 16090.87±4119.628 | 3.8 | 5.9 | d_9_-SM18:1 |
| SM24:1 | 42633.652±11075.085 | 4.8 | 6.5 | d_9_-SM18:1 |
| SM24:0 | 20245.469±8279.58 | 18.6 | 9.0 | d_9_-SM18:1 |
| GlcCer16:0 | 580.599±240.072 | 14.4 | 11.4 | d_5_-GlcCer18:0 |
| GlcCer18:1 | 3.108±1.034 | 8.8 | 9.8 | d_5_-GlcCer18:0 |
| GlcCer18:0 | 60.234±22.573 | 6.2 | 7.4 | d_5_-GlcCer18:0 |
| GlcCer24:1 | 863.095±343.251 | 8.9 | 10.3 | d_5_-GlcCer18:0 |
| LacCer12:0 | 3.461±1.573 | 12.5 | 12.5 | d_5_-GlcCer18:0 |
| LacCer16:0 | 1894.434±613.303 | 12.1 | 13.0 | LacCer17:0 |
| LacCer24:1 | 379.079±158.514 | 13.8 | 9.3 | LacCer17:0 |
| LacCer24:0 | 123.1±53.089 | 16.5 | 12.5 | LacCer17:0 |
| Cer1PC16:0 | 91.766±36.144 | 18.4 | 9.9 | d_7_-Cer18:0 |
| Cer1PC24:0 | 29.417±8.526 | 17.5 | 9.7 | d_7_-Cer24:1 |

Cer, ceramide; Cer1P, ceramide-1-phosphate; DhCer, dihydroceramide; GlcCer, glycosylceramide; LacCer, lactosylceramide; SM, sphingomyelin; Spa, sphinganine; Spa1P, sphinganine-1-phosphate; Sph, sphingosine; Sph1P, sphingosine-1-phosphate. Concentrations are represented as mean±SD. The study was performed in 633 patients from Study 1.

| Online-Table 6. Improvement of model performance for cardiac mortality in Study 1 with Cer16:0 and its derivatives. | | | | | |  |
| --- | --- | --- | --- | --- | --- | --- |
| Metabolite | **Youden** | **LR Model-1** | **LR Model-2** | **Change in LR** | ***P.value*** | |
| Cer16:0 | 206 | 23.45 | 29.14 | 5.69 | 0.01704 | |
| GlcC16:0 | 579 | 23.45 | 35.97 | 12.52 | 0.00040 | |
| Cer16:0/Cer22:0 | 0.34 | 23.45 | 32.52 | 9.07 | 0.00260 | |
| Cer16:0/Cer24:0 | 0.07 | 23.45 | 30.7 | 7.25 | 0.00708 | |

LR, Likelihood ratios. The analysis was performed in 633 patients from Study 1, with 17 cardiac deaths.

**Online-Table 7.** Adjusted hazard ratios for the prediction of cardiac mortality in Study 1, using ceramides as continuous variables.

| Metabolite | Adj. HR [95%Confidence Intervals]^ | P value |
| --- | --- | --- |
| Cer16:0 | 1.53 [1.12-2.09] | 0.008 |
| GlcC16:0 | 2.38 [1.59-3.60] | <0.001 |
| Cer16:0/Cer22:0 | 2.77 [1.78-4.29] | <0.001 |
| Cer16:0/Cer24:0 | 2.29 [1.67-3.13] | <0.001 |

^Cox models are adjusted for age, sex, hypertension, diabetes mellitus, smoking status (current, ex-smoker, non-smoker), BMI, total cholesterol, LDL, HDL, and triglycerides. The analysis was performed in 633 patients from Study 1, with 17 cardiac deaths.

**Online-Table 8.** Causes of death in Study 1 (Ox-HVF cohort).

| **#Patients** | **ICD10 code** | **Cause of death** | **Cardiovascular death** | **Cardiac death** |
| --- | --- | --- | --- | --- |
| 1-11 | C02.9;C20;C25.9;  C34.9;C41.1;  C54.1;C67.9;  C71.9; C83.7; D37.4 | Malignant neoplasm | No | No |
| 12, 13 | G12.2; G20 | Neuron disease | No | No |
| 14-16 | I21.9 | Acute myocardial infarction | Yes | Yes |
| 17-20 | I25.1 | Atherosclerotic heart disease | Yes | Yes |
| 21-26 | I25.9 | Chronic ischaemic heart disease | Yes | Yes |
| 27 | I26.9 | Pulmonary embolism | Yes | No |
| 34-37 | J84.9; J18.9 | Other pulmonary disease | No | No |
| 28-30 | I35.0 | Aortic (valve) disease | Yes | Yes |
| 31 | I48.9 | Atrial fibrillation | Yes | Yes |
| 32, 33 | I64 | Stroke | Yes | No |
| 40, 38 | K92.8; J84.9 | Digestive disease | No | No |
| 42, 41, 39 | N39.0; N17.9; K81.9 | Renal disease | No | No |
| 44, 43 | Y83.9; V49.4 | Other | No | No |

**Online-Table 9.** Association of ceramides with cardiovascular death in Study 1.

| Metabolite | Adj. HR [95%Confidence Intervals] | P value |
| --- | --- | --- |
| Cer16:0 | 1.48 [1.07-2.07] | 0.02 |
| GlcCer16:0 | 2.28 [1.53-3.40] | <0.001 |
| Cer16:0/Cer22:0 | 2.71 [1.78-4.13] | <0.001 |
| Cer16:0/Cer24:0 | 2.26 [1.67-3.05] | <0.001 |
| Adjusted hazard ratios are presented per SD increase.  Cox models are adjusted for age, sex, hypertension, diabetes mellitus, smoking status (current, ex-smoker, non-smoker), BMI, total cholesterol, LDL, HDL, and triglycerides. The analysis was performed in 633 patients from Study 1, with 20 cardiovascular deaths. | | |

**Online-Table 10.** Association of ceramides with the composite endpoint in Study 1.

| Metabolite | Adj. HR [95%Confidence Intervals] | P value |
| --- | --- | --- |
| Cer16:0 | 1.31 [0.96-1.80] | 0.09 |
| GlcCer16:0 | 1.72 [1.13-2.62] | 0.01 |
| Cer16:0/Cer22:0 | 2.11 [1.47-3.03] | <0.001 |
| Cer16:0/Cer24:0 | 1.94 [1.48-2.54] | <0.001 |
| Adjusted hazard ratios are presented per SD increase.  Cox models are adjusted for age, sex, hypertension, diabetes mellitus, smoking status (current, ex-smoker, non-smoker), BMI, total cholesterol, LDL, HDL, and triglycerides.  Composite endpoint includes cardiovascular death and/or hospital admission for heart failure. The analysis was performed in 633 patients from Study 1, where 29 patients met the composite endpoint. | | |

| **Online-Table 11.** Differential metabolites in 8 Weeks versus Day 0 of a weight loss period in a randomised clinical trial (Study 3). | | |
| --- | --- | --- |
| **Metabolites** | ***P*.value** | **FDR-adjusted *P*.value** |
| Cer12:0 | 4.6566E-10 | 9.3132E-09 |
| SM12:0 | 4.6566E-10 | 9.3132E-09 |
| LacCer12:0 | 9.3132E-10 | 1.2418E-08 |
| Cer14:0 | 1.397E-09 | 1.397E-08 |
| SM24:0 | 3.2596E-08 | 2.6077E-07 |
| SM18:1 | 5.1223E-08 | 3.4148E-07 |
| Cer24:0 | 6.3796E-08 | 3.6455E-07 |
| SM18:0 | 2.9802E-07 | 1.4901E-06 |
| SM24:1 | 4.2096E-07 | 1.8709E-06 |
| Cer22:0 | 5.872E-07 | 2.3488E-06 |
| LacCer24:0 | 0.000003512 | 0.000012771 |
| Cer20:0 | 4.0215E-06 | 0.000013405 |
| SM16:0 | 5.9749E-06 | 0.000018384 |
| LacCer18:0 | 6.7949E-06 | 0.000019414 |
| GlcCer24:1 | 0.000012635 | 0.000033694 |
| GlcCer18:0 | 0.00016188 | 0.00040471 |
| DhCer16:0 | 0.00023426 | 0.00055121 |
| Cer18:0 | 0.0002804 | 0.00062311 |
| LDL | 0.0003979 | 0.00083769 |
| DhCer24:0 | 0.00047182 | 0.00094363 |
| Cer1PC16:0 | 0.00071318 | 0.0013584 |
| Cer24:1 | 0.00077296 | 0.0014054 |
| LacCer16:0 | 0.00090613 | 0.0015759 |
| Cer17:0 | 0.0010594 | 0.0017656 |
| Insulin | 0.0011443 | 0.0018309 |
| Cholesterol | 0.001548 | 0.0023815 |
| Sph | 0.0047102 | 0.0069781 |
| Glucose | 0.0064787 | 0.0092553 |
| LacCer24:1 | 0.0073333 | 0.010115 |
| SM17:0 | 0.0082841 | 0.011045 |
| GlcCer18:1 | 0.030922 | 0.039899 |
| Spa1P | 0.043329 | 0.054161 |
| Cer18:1 | 0.064984 | 0.078768 |
| Cer1PC24:0 | 0.27813 | 0.32721 |
| Sph1P | 0.48833 | 0.55809 |
| HDL | 0.61169 | 0.67966 |
| Triglycerides | 0.73288 | 0.7923 |
| GlcCer16:0 | 0.87545 | 0.92152 |
| Spa | 0.94854 | 0.94854 |
| Cer16:0 | 0.94854 | 0.94854 |

Cer, ceramide; Cer1P, ceramide-1-phosphate; DhCer, dihydroceramide; GlcCer, glycosylceramide; LacCer, lactosylceramide; SM, sphingomyelin; Spa, sphinganine; Spa1P, sphinganine-1-phosphate; Sph, sphingosine; Sph1P, sphingosine-1-phosphate. *P* values Week 8 vs Day 0 were calculated by Wilcoxon signed-rank tests. The study was performed in 32 patients.

| **Online-Table 12.** Differential metabolites (Study 3) in weight maintenance period of 52 weeks with and without liraglutide treatment. | | | |
| --- | --- | --- | --- |
| **Metabolites** | **Control group**  **(FDR-adjusted *P*.value)** | | **Liraglutide group**  **(FDR-adjusted *P*.value)** |
| Cer12:0 | 0.00020345 | 0.0021085 | |
| Cer14:0 | 0.00020345 | 0.0021085 | |
| Cer1PC16:0 | 0.00020345 | 0.001709 | |
| Cer24:0 | 0.00020345 | 0.0015259 | |
| LacCer12:0 | 0.00020345 | 0.0020345 | |
| SM12:0 | 0.00020345 | 0.0015259 | |
| Cer1PC24:0 | 0.00030518 | 0.027562 | |
| SM24:0 | 0.00030518 | 0.0015259 | |
| Cer20:0 | 0.00067817 | 0.031006 | |
| LacCer24:0 | 0.0012207 | 0.0023475 | |
| Cer22:0 | 0.0014242 | 0.0044759 | |
| DhCer24:0 | 0.0014242 | 0.0021085 | |
| Cer17:0 | 0.0023475 | 0.055222 | |
| HDL | 0.0037493 | 0.0015259 | |
| Insulin | 0.0044759 | 0.50964 | |
| Cholesterol | 0.0047472 | 0.0037493 | |
| DhCer16:0 | 0.0047472 | 0.0023475 | |
| LDL | 0.0047472 | 0.0078987 | |
| Cer16:0 | 0.018372 | 0.97995 | |
| SM18:1 | 0.018372 | 0.0021085 | |
| GlcCer16:0 | 0.034761 | 0.22132 | |
| Cer18:0 | 0.109 | 0.0021085 | |
| SM17 | 0.109 | 0.25978 | |
| SM18:0 | 0.109 | 0.020413 | |
| LacCer24:1 | 0.1332 | 0.3363 | |
| GlcCer24:1 | 0.14376 | 0.8641 | |
| Glucose | 0.20551 | 0.21622 | |
| GlcCer18:1 | 0.20551 | 0.22132 | |
| SM16:0 | 0.21939 | 0.48939 | |
| LacCer16:0 | 0.29832 | 0.97995 | |
| LacCer18:0 | 0.29832 | 0.21622 | |
| Cer18:1 | 0.31528 | 0.0053406 | |
| Cer24:1 | 0.6402 | 0.3363 | |
| Sph | 0.66105 | 0.74283 | |
| Triglycerides | 0.72248 | 0.43549 | |
| GlcCer18:0 | 0.72276 | 0.38432 | |
| SM24:11 | 0.72276 | 0.13439 | |
| Sph1P | 0.74286 | 0.33029 | |
| Spa | 0.76263 | 0.80385 | |
| Spa1P | 0.89993 | 0.60362 | |

Cer, ceramide; Cer1P, ceramide-1-phosphate; DhCer, dihydroceramide; GlcCer, glycosylceramide; LacCer, lactosylceramide; SM, sphingomyelin; Spa, sphinganine; Spa1P, sphinganine-1-phosphate; Sph, sphingosine; Sph1P, sphingosine-1-phosphate. *P* values Week 52 vs Week 8 for Control group (n=16) and Liraglutide group (n=16) were calculated by Wilcoxon signed-rank tests.

**Online-Table 13.** Summary of study tests and key findings.

| *Test* | *Tissue* | *Comparison/Target* | *Question/hypothesis* | *Main findings* |
| --- | --- | --- | --- | --- |
| *Computerized tomography (CT) imaging* | Adipose tissue | ThAT *vs.* ScAT | Relationship of fat volume with O_2_^.-^production in patients' vessels | The volume of ThAT but not ScAT was found to be positively related with O_2_^.-^production in IMA after adjustment for traditional risk factors |
| *Metabolomics profiling* | Adipose tissue secretome | ThAT *vs.* ScAT | Differential metabolites and pathways | 82 metabolites with significant differential patterns between the ThAT and ScAT were revealed and mapped to 21 metabolic pathways |
|  |  | Obese *vs.* lean | Differential metabolites and pathways | Identified differential metabolites in ScAT and ThAT secretome with their overlapping hits |
|  |  |  |  | SPL metabolism scored the highest pathway impact of the mapped metabolic pathways in the secretome of ThAT obtained from obese as compared to lean patients |
| *Sphingolipid (SPL) Targeted measurement* | Adipose tissue secretome | ThAT *vs*. ScAT | Validation of differential patterns and relationship with impaired endothelial function | SPL secretion and production levels were identified in ThAT and ScAT. |
|  |  |  |  | SPL secretion and production levels are higher in ThAT |
|  | Adipose tissue biopsies |  |  | High levels of ThAT-derived SPL are associated with increased O_2_^.-^ production and impaired endothelial function in human vessels |
|  | Plasma | SPL | Measure circulating SPL in cohort | SPL circulating levels were quantified |
|  | ThAT adipocytes extracellular vesicles (EV) | SPL | Measure SPL in EV released from ThAT adipocytes | ThAT primary adipocytes produce and release SPL via EV enriched in Cer16:0 |
| *Cohort-wide association analysis* | Plasma | Ceramides *vs.* CVD traditional risk factors | Identify CVD high-risk ceramides | Circulating Cer16:0 positively associated with hsCRP, a marker of systemic inflammation |
|  | Internal mammary arteries (IMA) | Ceramides circulating levels *vs.*:  1) O_2_^.-^ production in patients vessels  2) L-NAME inhibitable O_2_^.-^  3)Endothelial dependent and independent vasorelaxation | Identify ceramides that associate with pathogenic changes in endothelial redox state | All long chain ceramides (C16:0-C18:1) associated with increased O_2_^.-^ production. |
|  |  |  |  | Only Cer16:0 associated with eNOS uncoupling, as determined by L-NAME-inhibitable O_2_^.-^in human arteries. |
|  |  |  |  | Plasma Cer16:0 levels were not correlated with NADPH-stimulated O_2_^.-^ generated by NADPH-oxidases. |
|  |  |  |  | Circulating Cer16:0 associated with reduced vasorelaxation of human vessels in response to ACh but not to SNP |
|  | Adipose tissue | Ceramides *vs.* adipose tissue-derived SPL | Detect a metabolic link between AT-derived SPL and ceramide pool in circulation | Circulating Cer16:0 positively associated with SPL levels in ThAT but not in ScAT |
| *In-vitro mechanistic investigation* | Human aortic endothelial cells (HAEC) | Effect of Cer16:0 on O_2_^.-^ production and eNOS phosphorylation/coupling in endothelial cells | Role of Cer16:0 in modulating human endothelial redox state via eNOS | Cer16:0increase intracellular O_2_^.-^ production |
|  |  |  |  | Cer16:0 increase L-NAME inhibitable O_2_^.-^ |
|  |  |  |  | Cer16:0 reduce BH4 (leading to eNOS uncoupling) |
|  |  |  |  | Cer16:0 reduce eNOS activity (phosphorylation) via PP2A but not AKT |
| *Survival analysis after 5.5 years of follow-up* | Plasma | Concentrations of circulating C16:0-SPL vsthe number of CVD events after 5.5 years follow-up | Predictive power of Cer16:0 and its metabolites for cardiac mortality | Cer16:0 and GlcCer16:0 predict cardiac death independent of other risk factors |
| *Liraglutide Randomised Clinical Trial* | Plasma | Concentration of circulating SPL after weight loss and 52 weeks treatment with liraglutide | Effect of weight loss and liraglutide on circulating levels of SPL | Liraglutide treatment regulate Cer16:0 and GlcCer16:0 circulating levels |

CVD, Cardiovascular Disease; ThAT, Thoracic Adipose Tissue; ScAT, Subcutaneous Adipose Tissue; O_2_^.-^ , Superoxide; Cer, Ceramide; GlcCer, Glycosylceramide.

**-ONLINE FIGURES-**

**
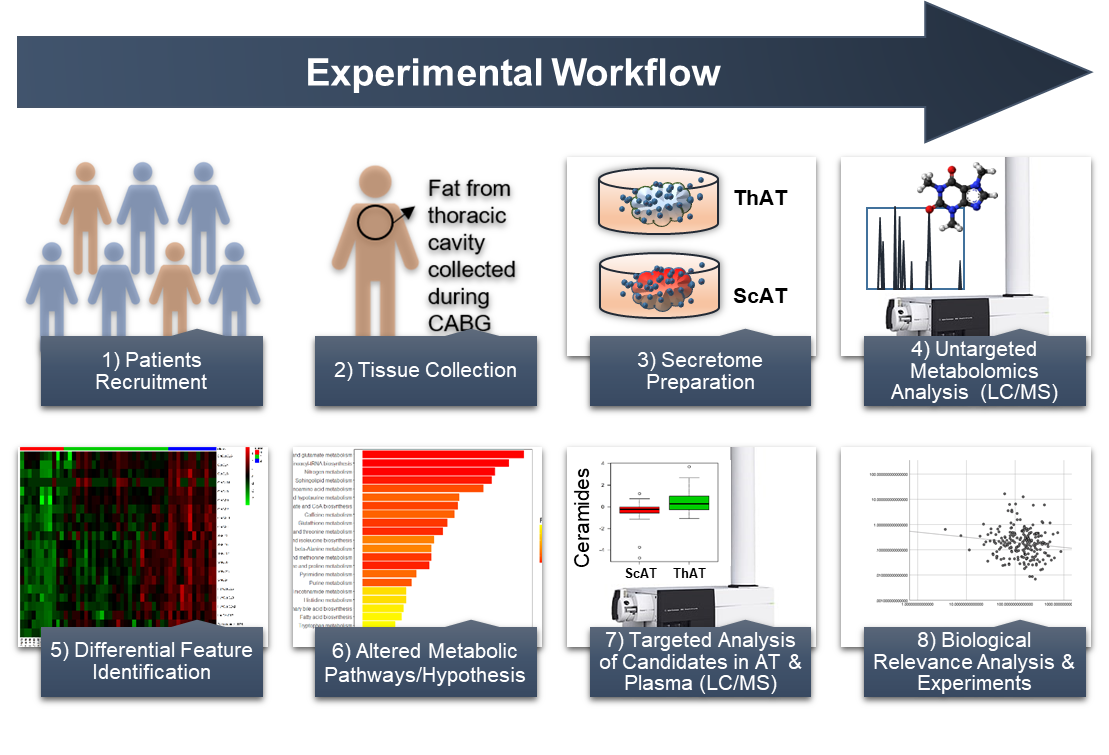
**

**Online-Figure 1: Schematic representation of experimental workflow.** Paired thoracic (ThAT) and subcutaneous (ScAT) depots collected from patients during surgery (Study 2) were immediately transported in warm transfer medium to a tissue culture hood, where they were placed in fresh transfer medium and cut into ∼1 mm^3^ pieces**.** Minced explants were rinsed in PBS and incubated in weight-adjusted volume of Medium-199 for 4 h at 37°C in 5% CO_2_. Tissues were separated from the medium after the incubation and stored at -80°C. The medium was harvested and stored at -80°C. A total of 96 fat samples (48 ScAT and 48 ThAT) and corresponding sets of conditioned media were analysed using the Q-TOF-MS metabolomics and targeted platforms (Agilent-6550). A list of differential metabolites were enriched in several dysregulated metabolisms. Top ranked metabolites were validated using targeted platform in fat tissues (Study 2, n=96) and corresponding secretome (Study 2, n=96) as well as in a large cohort of plasma samples (Study 1, n= 633). Statistical analysis as well as mechanistic lab experiments were carried out to understand the role of discovered candidates (i.e. ceramides) in in the regulation of vascular redox signaling and the vascular complications of obesity.

**
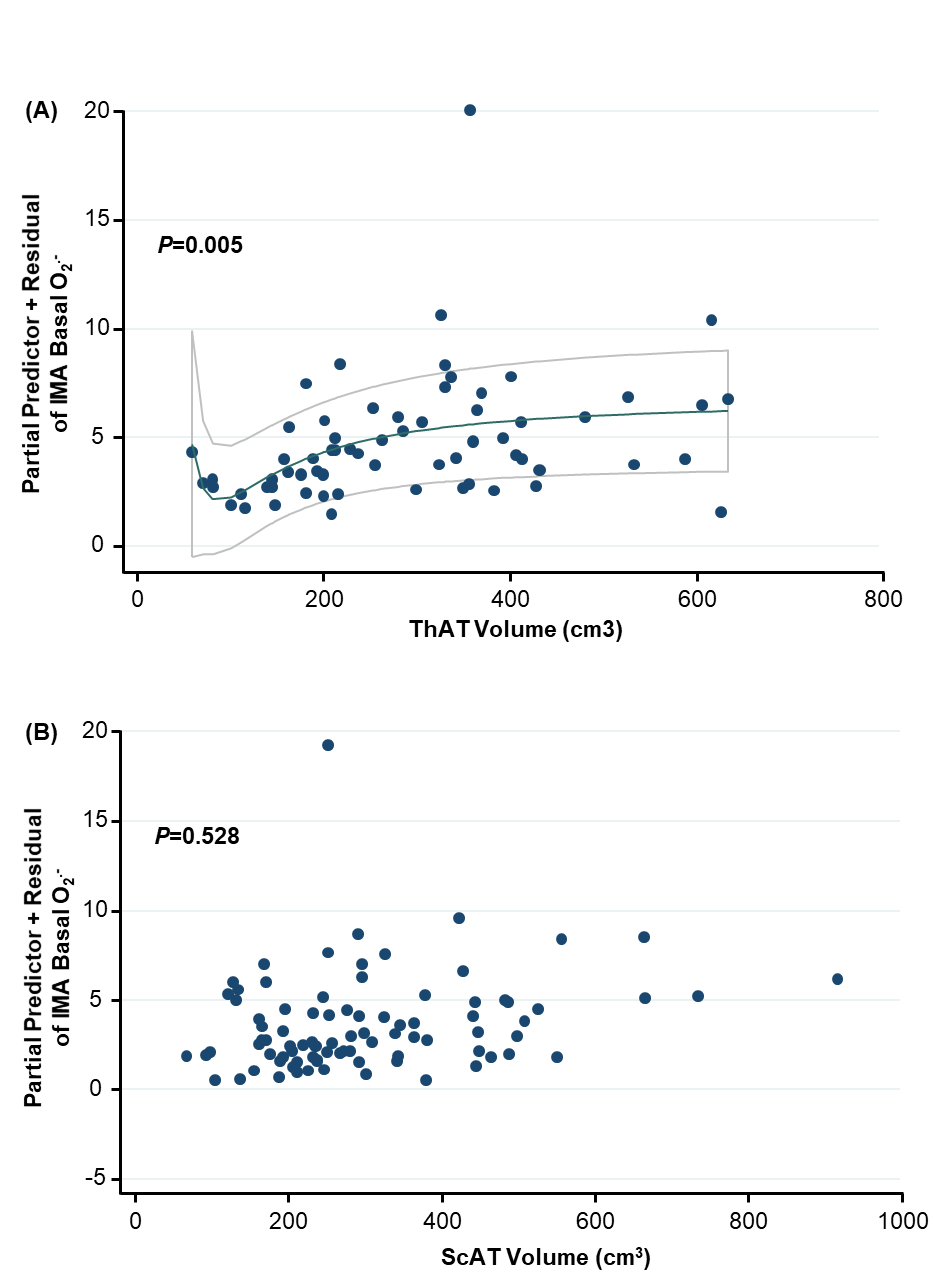
**

**Online-Figure 2: Relationship between vascular superoxide production levels with the volumes of thoracic visceral and subcutaneous fat.**

Fractional polynomial adjusted for covariates (age, gender, diabetes and hypercholestermia) for visceral thoracic fat volume (ThAT) **(A)** and subcutaneous fat volume (ScAT) **(B)** (Study 1, n=87). The best powers of ThAT volume among 44 models were -2 and -2 with the deviance of 307.88, and the best powers of ScAT volume among 44 models were -2 and 2 with the deviance of 414.09.  IMA, internal mammary arteries; O_2_^.-^, superoxide.


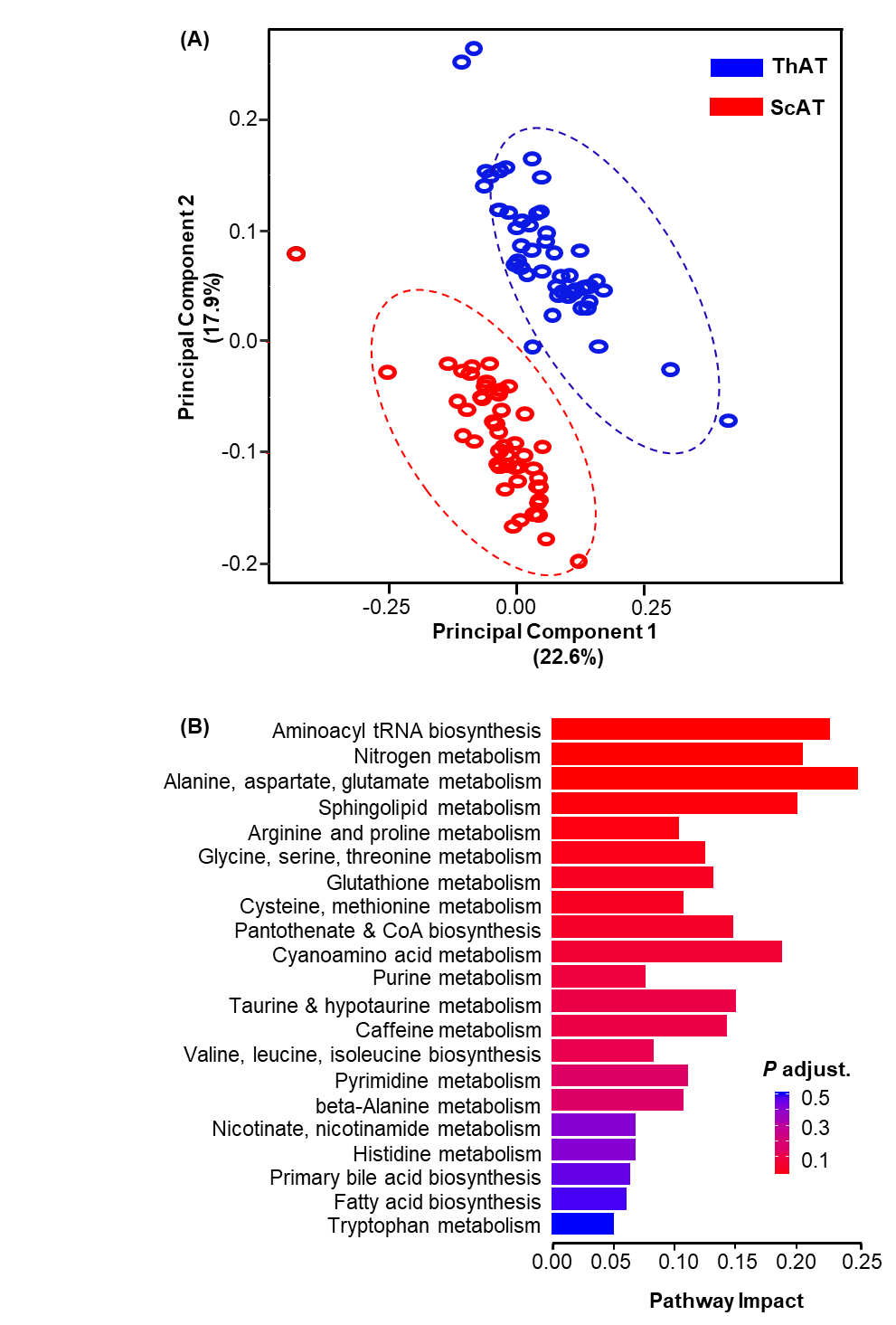
**
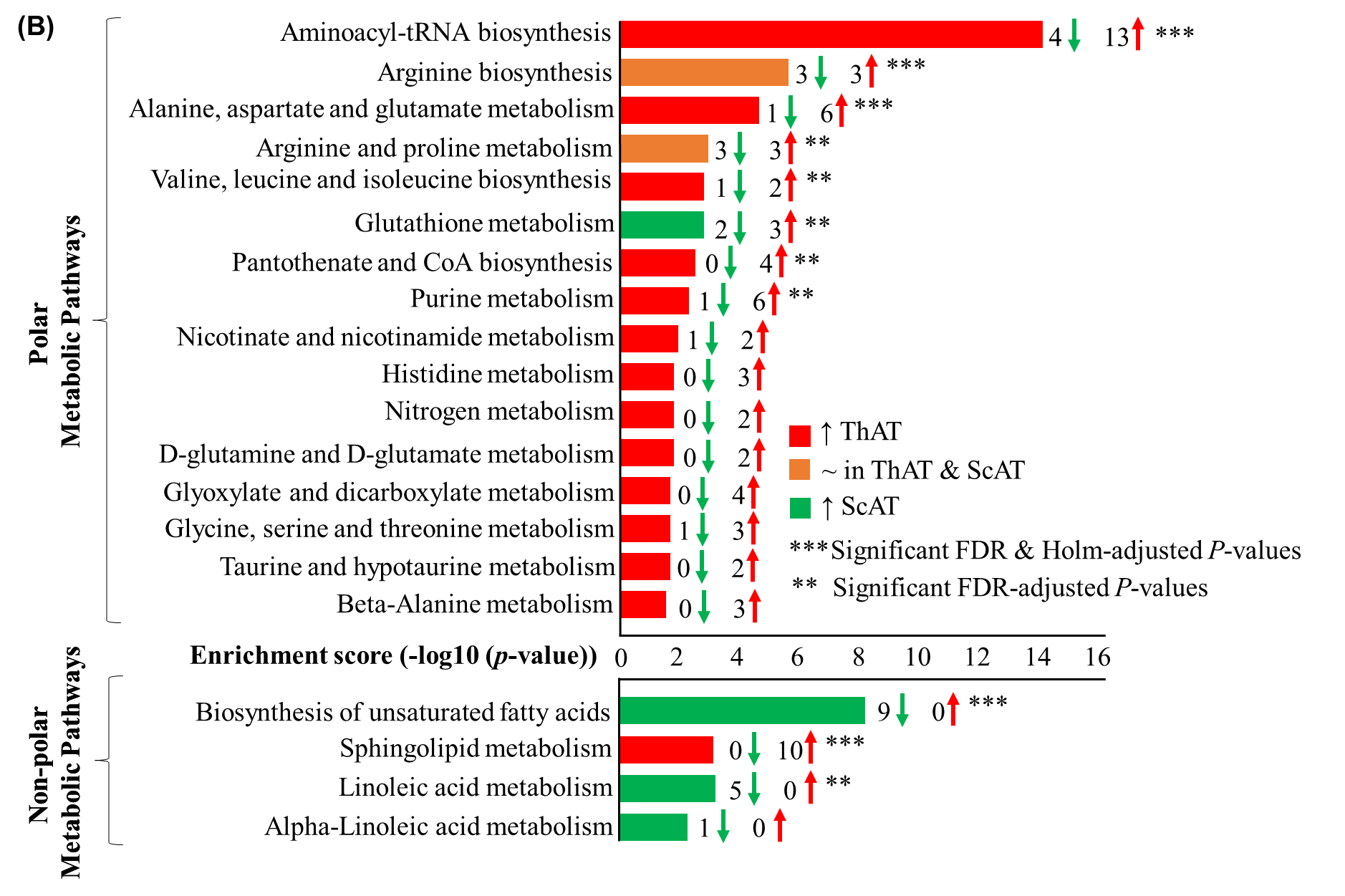
**

**Online-Figure 3: Differential metabolic profiles and pathways in ThAT Secretome versus ScAT Secretome**

**A)** Principal component analysis (PCA) score plot based on 48 thoracic (ThAT) and 48 subcutaneous (ScAT) adipose tissue secretome metabolic profiles of cardiac patients (Study 2, n=96) showed that ThAT and ScAT profiles were clustered separately. **B)** Up and down-regulated polar and non-polar pathways in the secretome of ThAT vs. the secretome of ScAT (Study 2, n=96). The bar chart showing significant pathways with raw *p*-value <0.05, ** indicate pathways with FDR-adjusted *p*-value < 0.05 and *** indicates pathways with FDR & Holm-adjusted *p*-value < 0.05. All pathways are ordered based on their enrichment scores (-log10 (*p*-value)).

**
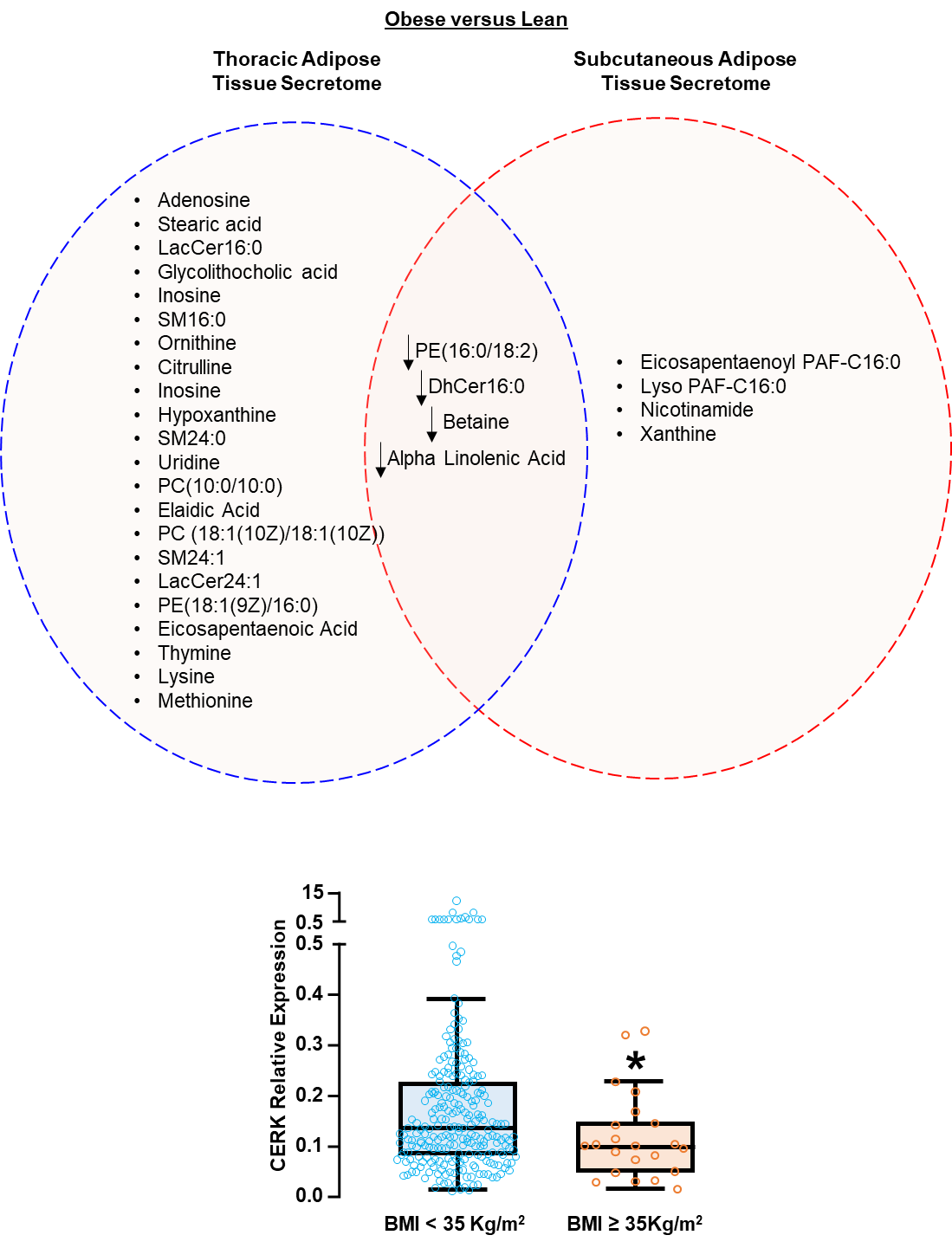
Online-Figure 4. Obesity-related dysregulation of the metabolomic profile of ThAT and ScAT secretome.** Metabolomics profiling of adipose tissue secretome obtained from obese (Study 2, n=31) and lean (Study 2, n=17) patients revealed 26 obesity-related differential metabolites secreted from ThAT and 8 differential metabolites secreted from ScAT. Four metabolites were significantly reduced in the secretome of both adipose tissue depots. PE, phosphatidylethanolamine; DhCer, dihydroceramide; LacCer, lactosylceramide; SM, sphingomyelin; PC, Phosphatidylcholines; PAF, platelet activating factor.


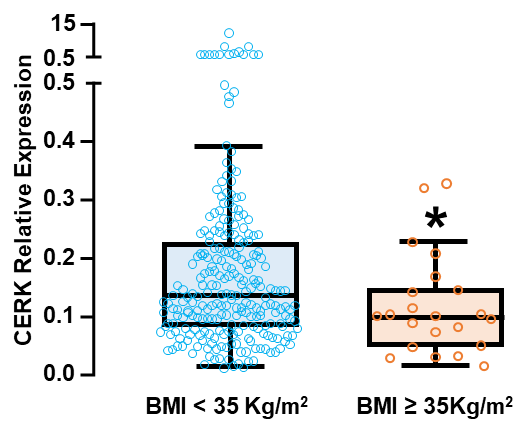


**Online-Figure 5. Reduced expression of CERK enzyme in obesity.** Expression of CERK gene (relative to *PPIA*) in ThAT obtained from obese patients with BMI ≥ 35 Kg/m^2^ (Study 1, n=22) versus ThAT obtained from patients with BMI < 35 (Study 1, n=247). Boxplots represent Median[25^th^-75^th^ percentile]. **p*<0.05 was calculated by Mann-Whitney U test.


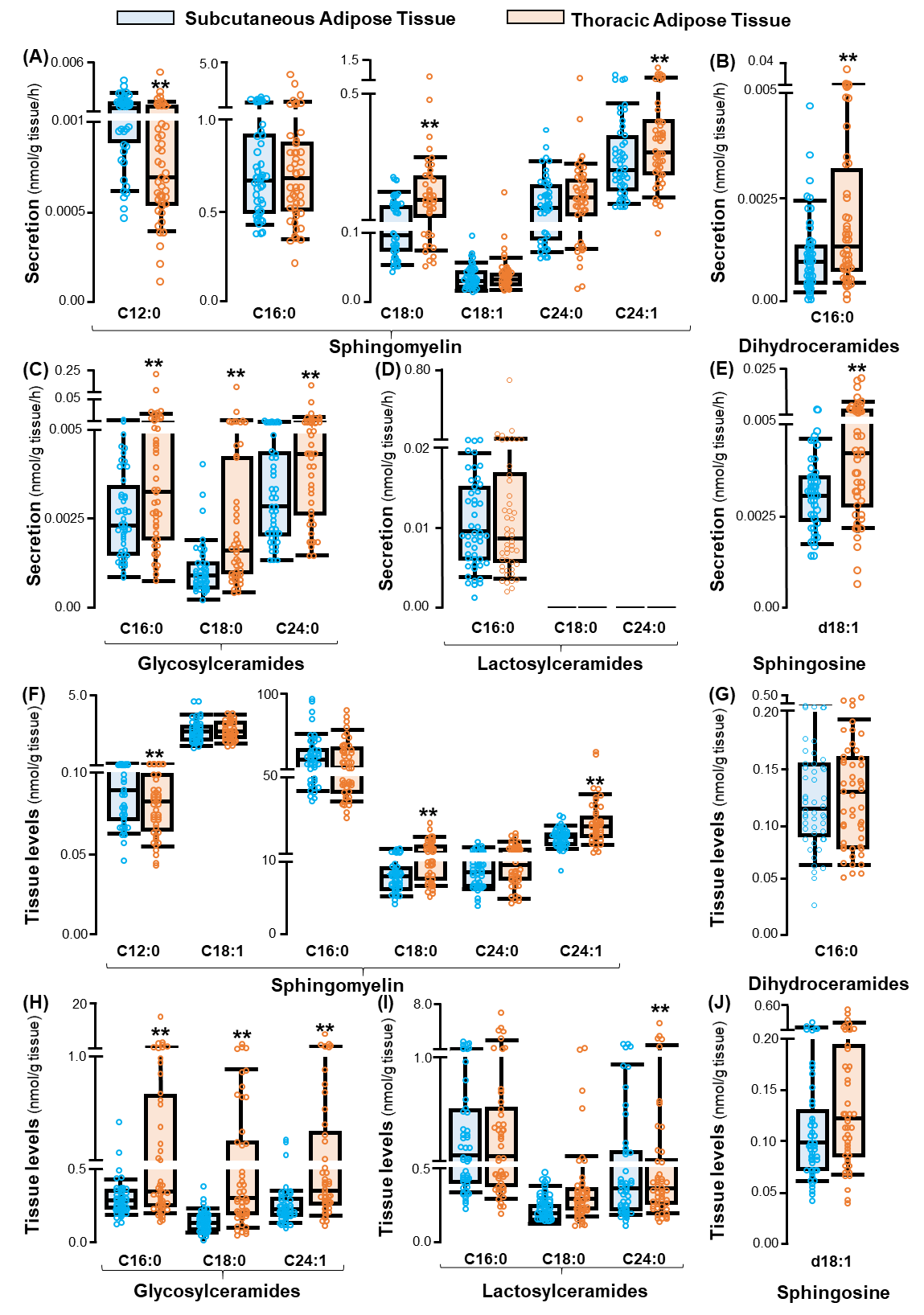


**Online-Figure 6. Differential synthesis and secretion patterns of sphingolipid (SPL) in human thoracic versus subcutaneous adipose tissue**. SPL concentrations were measured in adipose tissues (Study 2, *n*=96) and their secretomes (Study 2, *n*=96) by LC-MS/MS. SPL secretion levels in thoracic (ThAT) and subcutaneous (ScAT) adipose tissue **(A-D)**. SPL synthesis pattern in ThAT versus ScAT **(F-J)**. SPL secretion and synthesis were higher in human ThAT compared to ScAT and C16:0 species were the most abundant adipose SPL. Boxplots represent the Median[25^th^-75^th^ percentile]; ***p*<0.05 (FDR-adjusted) ThAT versus ScAT were calculated by Wilcoxon signed-rank tests.

**
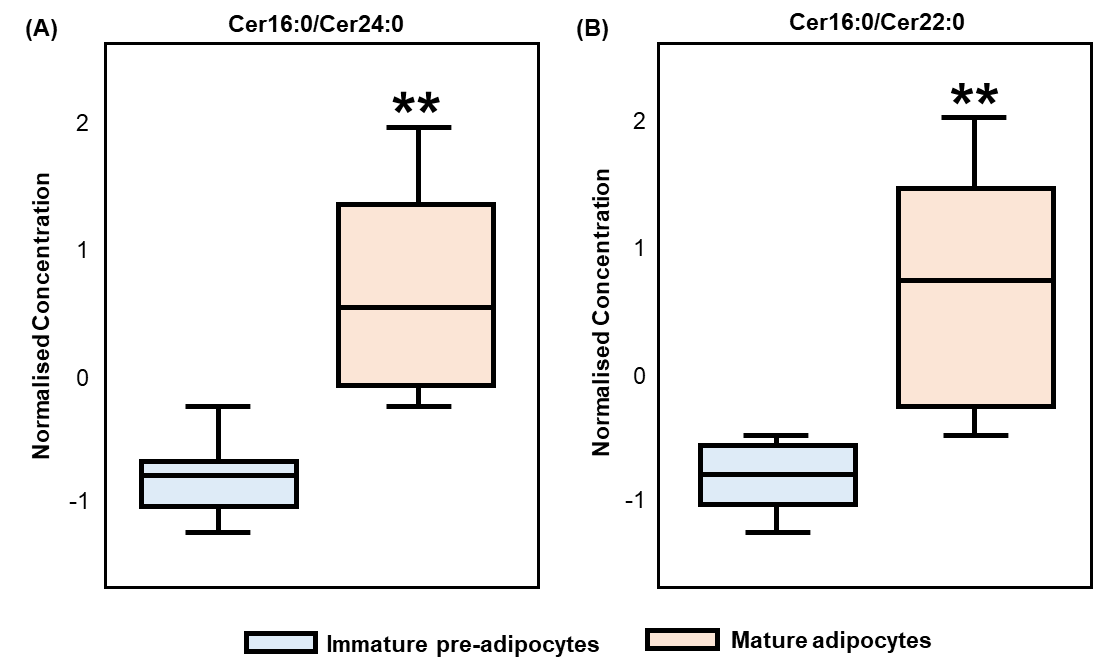
**

**Online-Figure 7. The effect of primary adipocytes differentiation on Cer16:0/Cer22:0 and Cer16:0/Cer24:0 ratios.** Box plots showing the significant difference in the ratios of Cer16:0 to Cer24:0 **(A)** and Cer22:0 **(B)** in primary adipocytes cultured from ThAT biopsies of cardiac patients before and after differentiation (Study 1, *n*=10). **FDR-adjusted *p*<0.05 before vs. after differentiation were calculated by Wilcoxon signed-rank tests. Cer, ceramide.


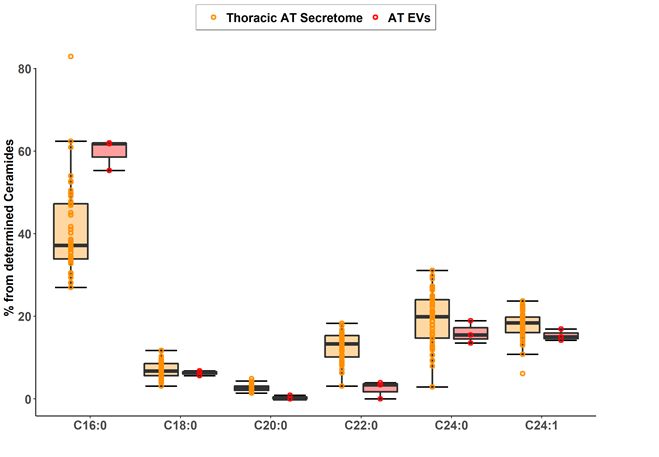


**Online-Figure 8.** **Comparison of the relative ceramide composition between ThAT-derived extracellular vesicles and ThAT secretome.** Ceramides were determined both in conditioned media (Study 2, n=48 patients) and extracellular vesicles from ThAT secretome. The percentage of each ceramide species relative to all determined ceramides was determined in ThAT secretome and corresponding extracellular vesicles. ThAT-derived EVs are enriched in Cer16:0 relative to the other ceramides. Boxplots represent the Median[25^th^-75^th^ percentile].

**
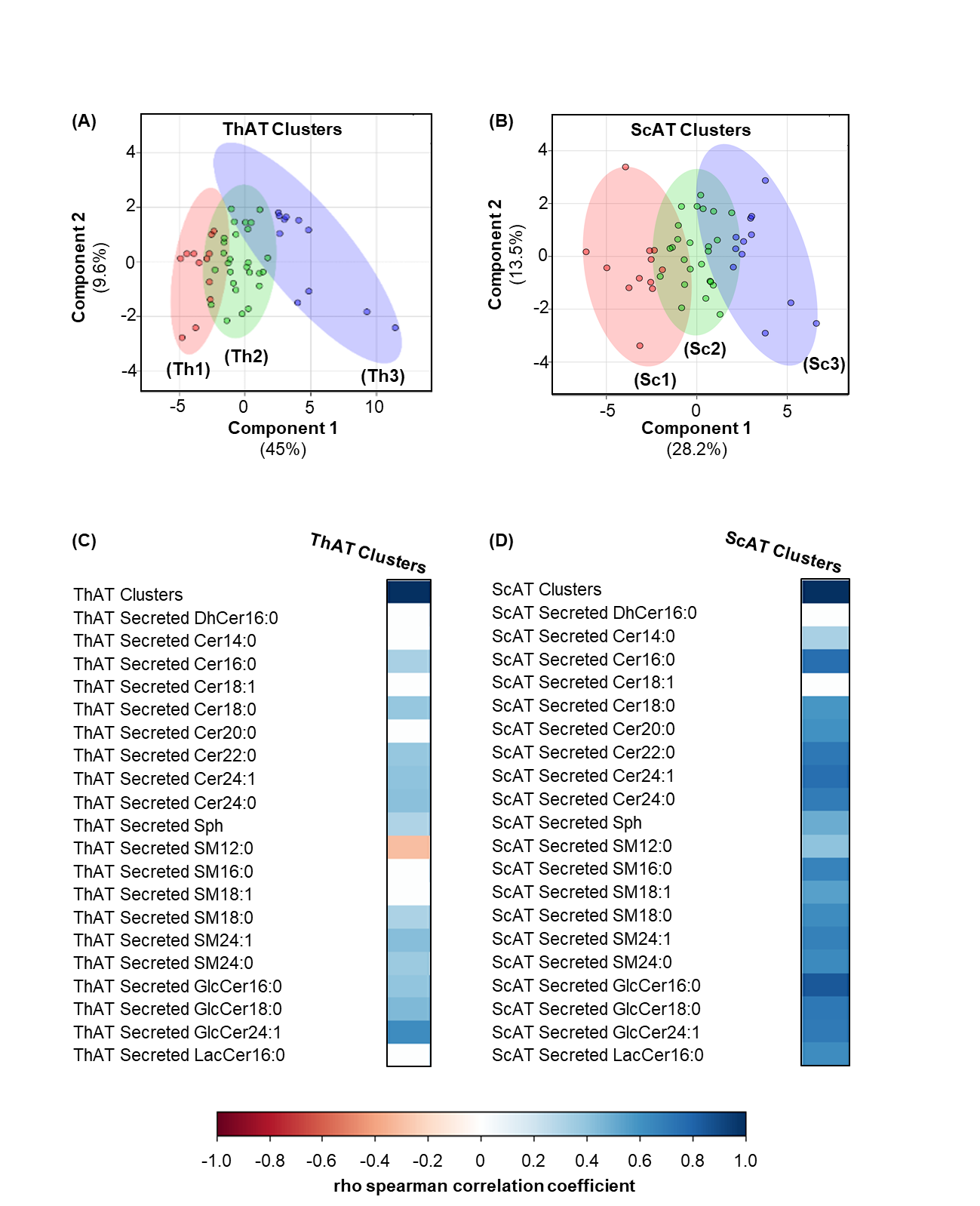
**

**Online-Figure 9. Adipose sphingolipids clusters correlated with thoracic adipose tissue (ThAT) and subcutaneous (ScAT) secreted sphingolipids. (A-B)** Principal component analysis (PCA) score plots showed that ThAT **(A)** and ScAT **(B)** sphingolipids were grouped in separated clusters (Study 2). Th1, Th2, and Th3 are clusters of sphingolipids detected in ThAT biopsies (Study 2, n=48) and Sc1, Sc2 and Sc3 clusters of sphingolipids detected in ScAT biopsies (Study 2, n=48). **(C-D)** Correlation plots showing the association of the measured sphingolipids in the secretomes of ThAT **(C)** and ScAT **(D)** with their corresponding clusters. Blank squares represent Spearman’s rho correlation significance of *p*>0.05. Cer, ceramide; DhCer, dihydroceramide; GlcCer, glucosylceramide; LacCer, lactosylceramide; SM, sphingomyelin; Sph, sphingosine.


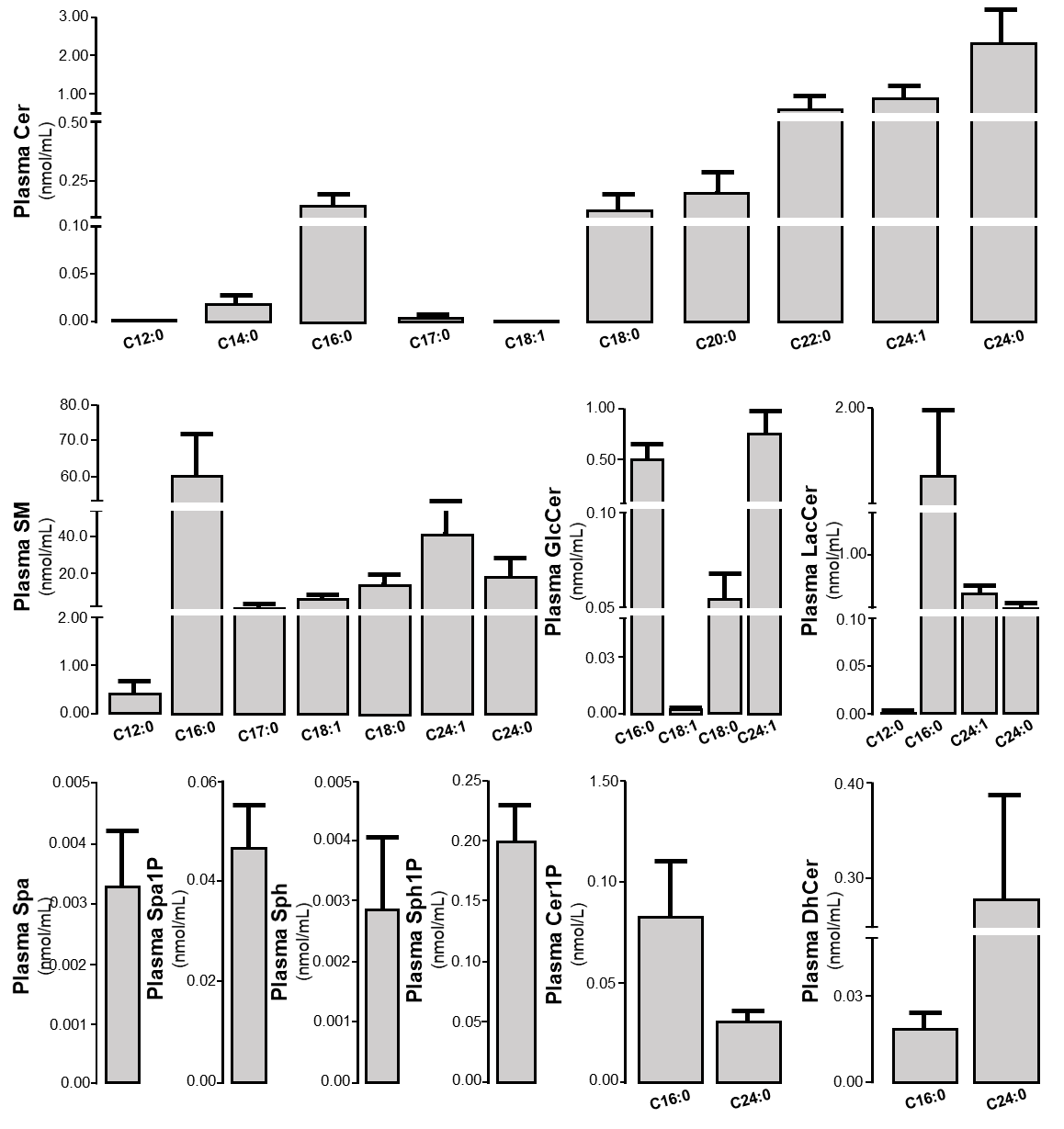


**Online-Figure 10. The metabolic profile of plasma sphingolipids of patients with coronary artery disease.** Concentrations of 33 sphingolipid metabolites in the plasma of 633 cardiac patient (Study 1). Sphingolipids were measured and identified by liquid chromatography tandem-mass spectrometry in plasma samples obtained from patients undergoing coronary artery bypass graft. Results are represented as median±IQR. Cer, ceramide; Cer1P, ceramide-1-phosphate; DhCer, dihydroceramide; GlcCer, glucosylceramide; LacCer, lactosylceramide; SM, sphingomyelin; Spa, sphinganine; Spa1P, sphinganine-1-phosphate; Sph, sphingosine; Sph1P, sphingosine-1-phosphate.

**
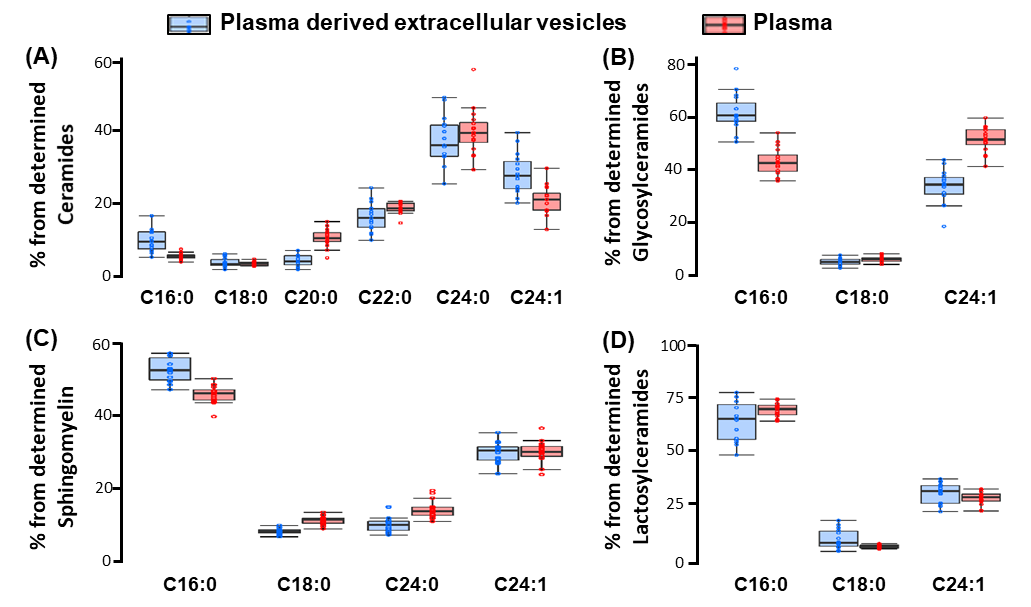
**

**Online-Figure 11.** **Comparison of the relative sphingolipid composition between circulating extracellular vesicles and plasma.** Sphingolipids were determined both in plasma and its derived extracellular vesicles (EVs; Study 1, n=15). Each sphingolipid was determined individually in EVs and plasma. The percentage of each sphingolipid was determined per class (A: Ceramides, B: Glycosylceramides, C: Sphingomyelin, D: Lactosylceramides). Plasma-derived EVs are enriched in C16:0-SPL species (except Lactosylceramide) relative to the other species in every class. Boxplots represent the Median[25^th^-75^th^ percentile].

.

**
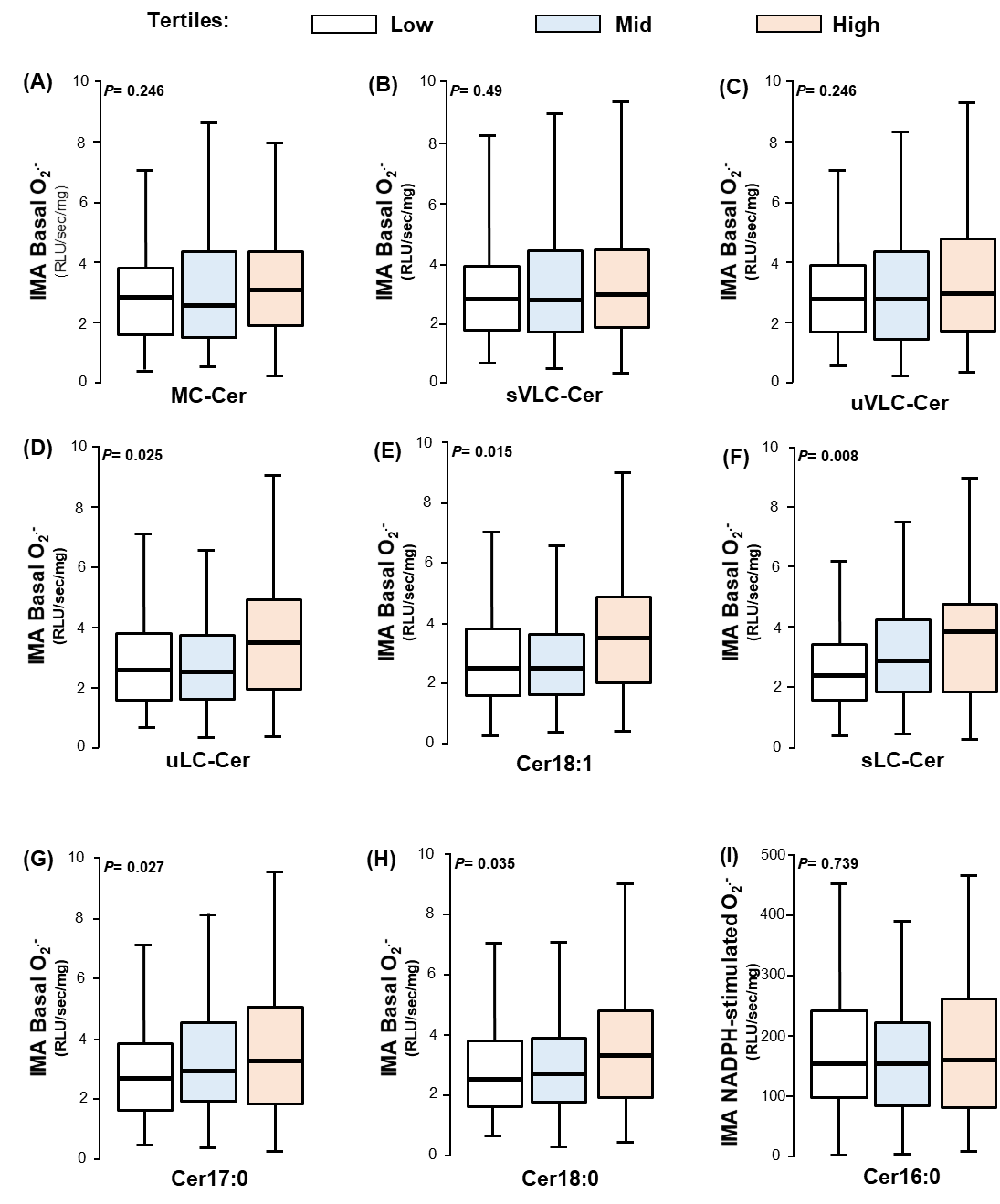
**

**Online-Figure 12. Associations of plasma ceramides with vascular superoxide levels.** Enhanced production of superoxide (O_2_^.-^) in patients’ intermammary arteries (IMA; Study 1) was not increased in patients with high concentrations of MC-Cer **(A)**, sVLC-Cer **(B)**, and uVLC-Cer **(C)**. Higher levels of circulating uLC-Cer **(D-E)** and sLC-Cer **(F-H)** were associated significantly with increased O_2_^.-^ in patients’ IMA. Circulating levels of Cer16:0 were not correlated with NADP-stimulated O_2_^.-^ in patients’ arteries **(I)**. *P* values were calculated by Kruskal Wallis tests. Cer, ceramide; MC-Cer, medium chain ceramides (Cer12:0, Cer14:0); sLC-Cer, saturated long chain ceramides (Cer16:0, Cer17:0, Cer18:0); uLC-Cer, unsaturated long chain ceramide (Cer18:1); sVLC, saturated very long chain ceramides (Cer20:0, Cer22:0, Cer24:0); uVLC, unsaturated very long chain ceramide (Cer24:1). Boxplots represent the Median[25^th^-75^th^ percentile].


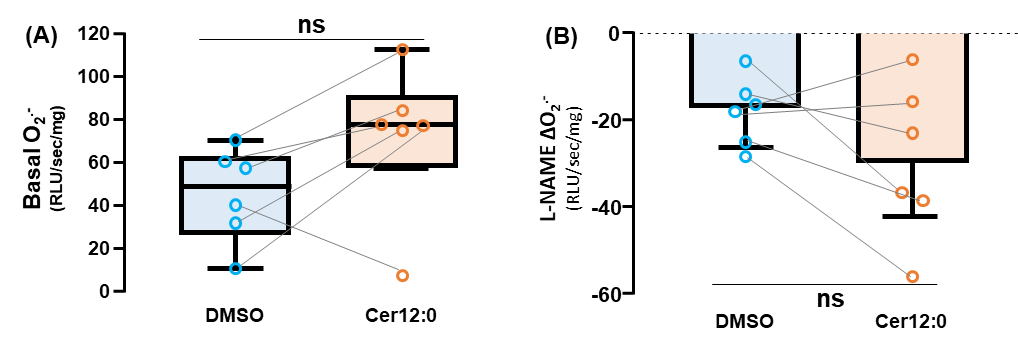


**Online-Figure 13.** **Effect of exogenous ceramides on superoxide (O_2_^.-^) in human aortic endothelial cells (HAEC).** Cer12:0 had no effect on the levels of superoxide (A; n=6) or L-NAME inhibitable O_2_^.-^ (B; *n*=6). **P*<0.05 versus control (DMSO <1%) were calculated by Wilcoxon signed-rank tests. Boxplots represent Median[25^th^-75^th^ percentile] and bar graphs mean+/-SEM.


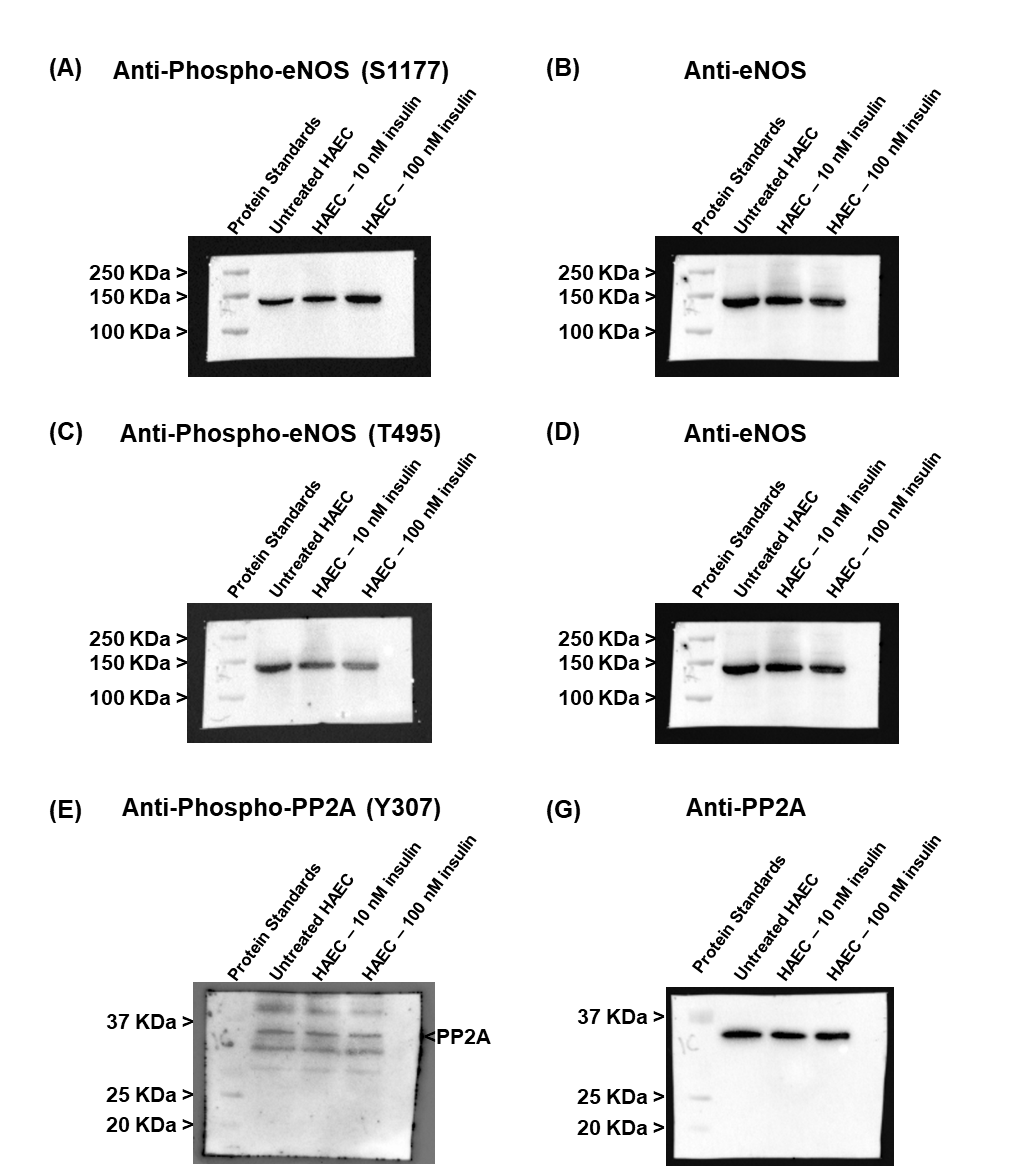


**Online-Figure 14. Antibodies controls.** HAEC were treated for 30 minutes with 10 nM, 100 nM insulin or nothing and assessed for eNOS (A,C), PP2A (E) phosphorylation by Western Blot. As a control also pan-eNOS (133 KDa, B, D), and pan-PP2A (36 KDa, G) were evaluated by Western Blot.

**
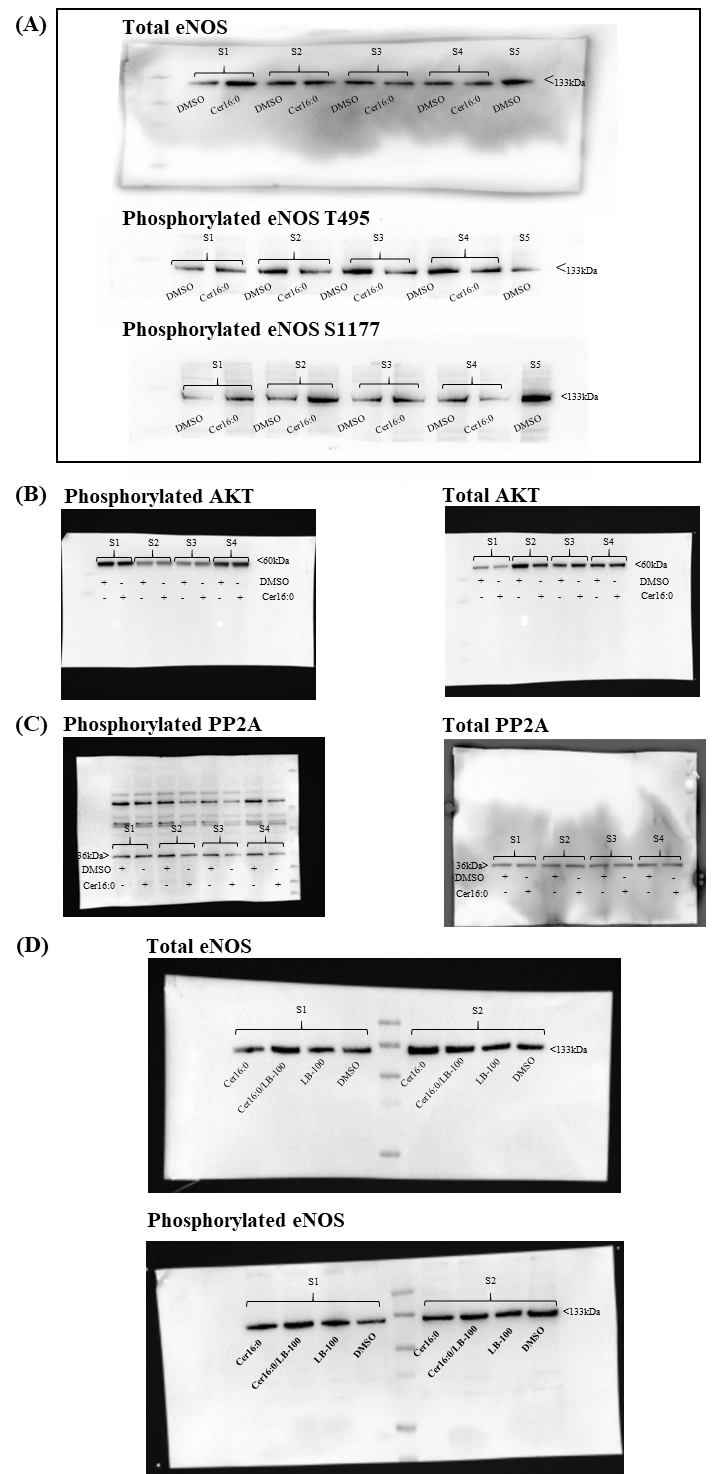
**

**Online-Figure 15. The uncropped representative immunoblots of the *in-vitro* mechanistic investigations.** Uncropped gels of the *in-vitro* mechanistic studies for eNOS (A), AKT (B), PP2A (C), LB100 (D) in Figure 7 of the main manuscript.

**
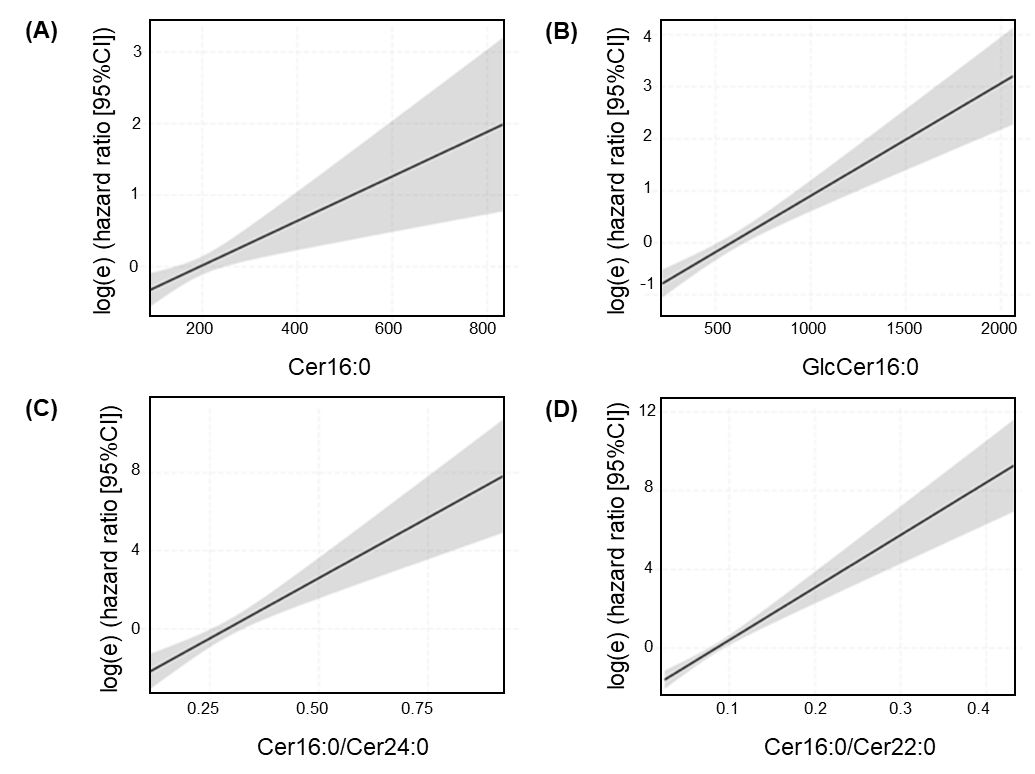
**

**Online-Figure 16. Fractional polynomial models to assess possible non-linear associations between metabolites and mortality risk.** Best fitting models using a fractional polynomials approach for metabolites (A: Cer16:0, B: GlcCer16:0, C: Cer16:0/Cer24:0, D: Cer16:0/Cer22:0) as predictors of cardiac mortality (Study 1). All models demonstrate a linear association (best fit) between metabolites and prospective mortality risk. Models are adjusted for age, sex, hypertension, diabetes, current smoking status, BMI, total cholesterol, LDL, HDL, triglycerides.


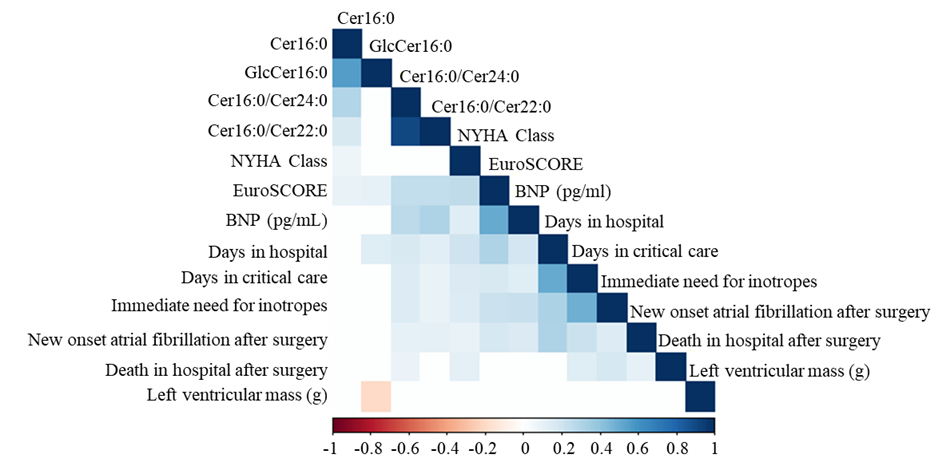
**Online-Figure 17. Associated phenotypes with Circulating Cer16:0, GlcCer16:0, Cer16:0/Cer24:0 and Cer16:0/Cer22:0.** High concentrations of Cer16:0 (Study 1) associated with higher NYHA classes and EuroSCORE, which is calculated based on the model developed by Nashef et al European Journal of Cardio-Thoracic Surgery 2012; 41:734–745. Cer16:0 ratios to Cer24:0 and Cer22:0 were found to be associated with worse cardiac outcomes. Cer, ceramide; BNP, Brain natriuretic peptide; GlcCer, glycosylceramide; NYHA, New York Heart Association.


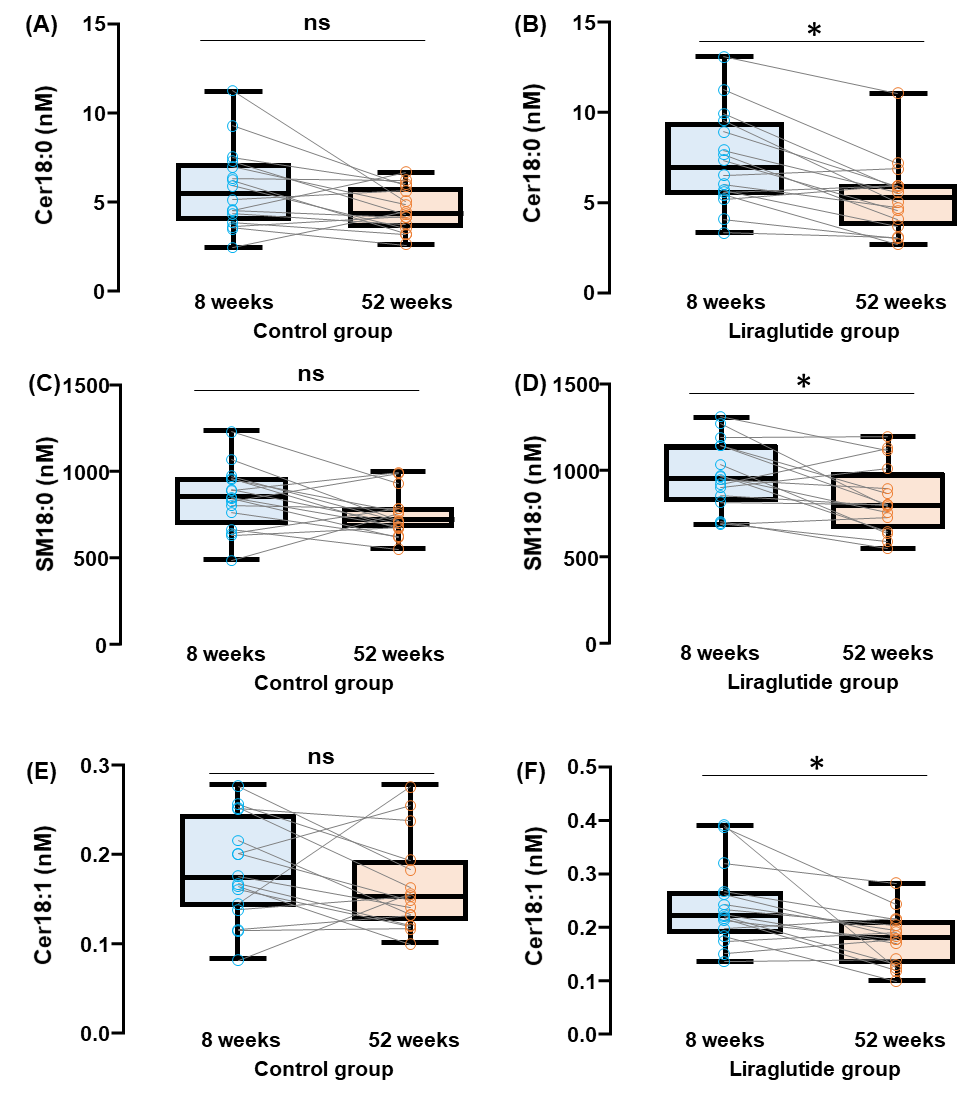


**Online-Figure 18. The effect of weight loss and liraglutide treatment on sphingolipids levels.** Box plots represent changes in Cer18:0 (A-B), SM18:0 (C-D), Cer18:1 (E-F) BMI-normalised concentration in the liraglutide treated group and in the non-treated group after 52 weeks of weight loss maintenance (Study 3, Median[25^th^-75^th^ percentile]). *FDR-adjusted p<0.05 52 weeks vs. 8 weeks were calculated by Wilcoxon signed-rank tests. Cer, ceramide; SM, Sphingomyelin.


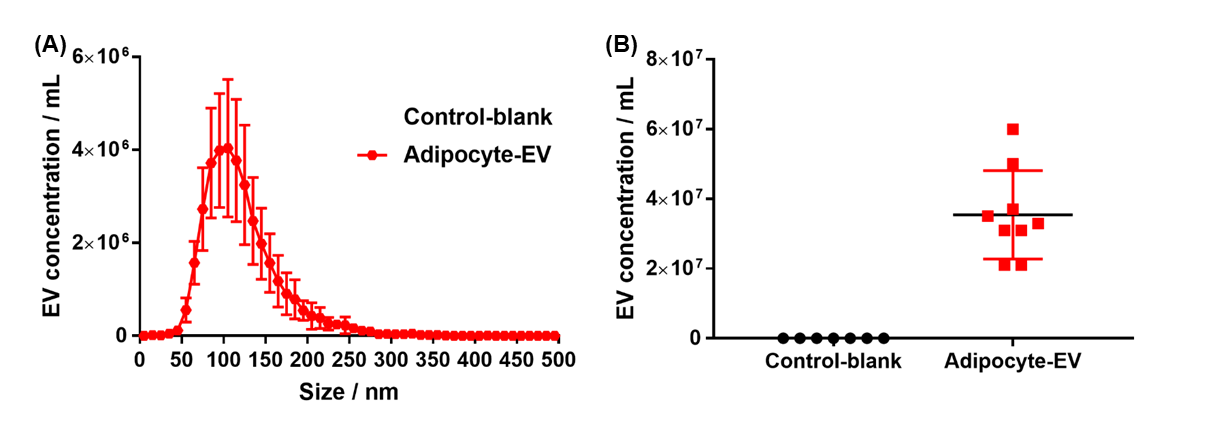


**(C)**

**
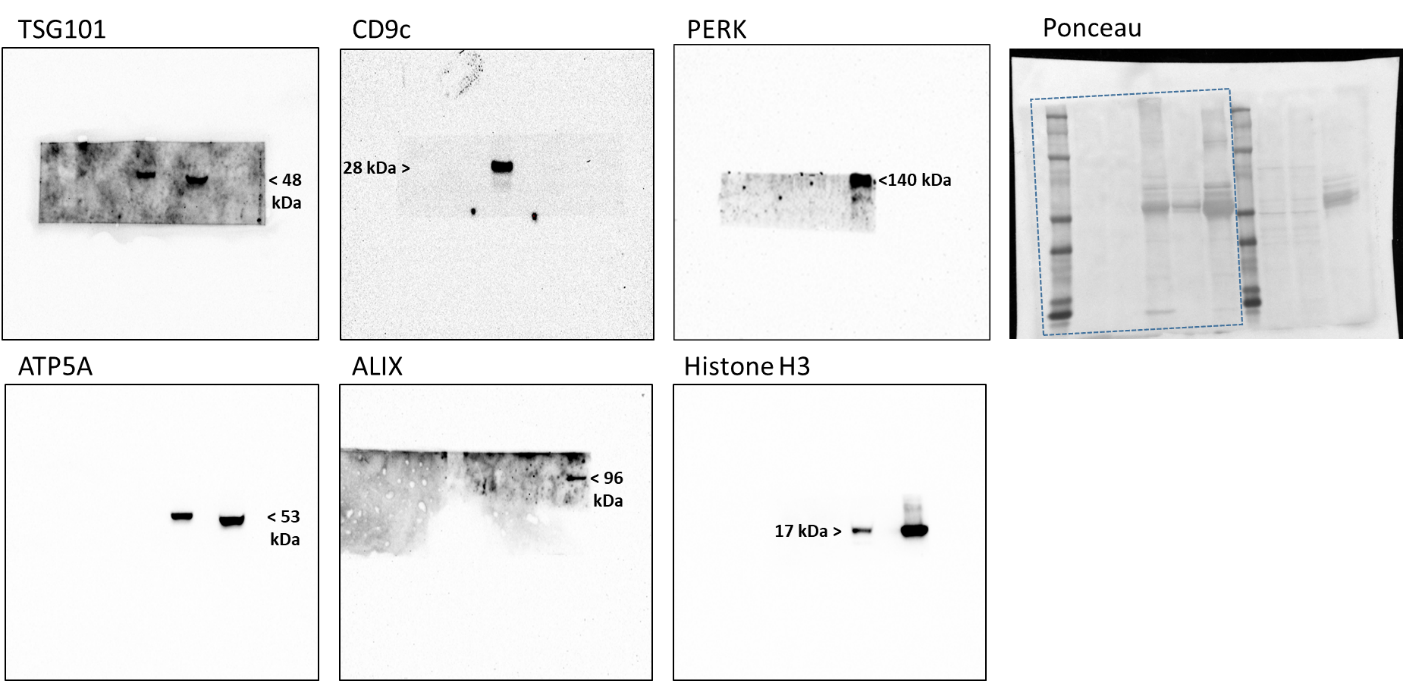
**

**Online-Figure 19. Isolation and characterisation of adipocyte extracellular vesicles.** Adipocyte extracellular vesicles (EV) (*n*=9) size **(A)** and concentration **(B)** profile were determined by Nanoparticle Tracking Analysis using a ZetaView instrument, a media only sham was used as a negative control (*n*=7). Results are mean±SD. Uncropped western blot membranes of EV and cell markers **(C)**.

**Online-Figure 20. Isolation and characterisation of circulating extracellular vesicles.** Circulating extracellular vesicles (EV) (*n*=16) size **(A)** and concentration **(B)** profile were determined by Nanoparticle Tracking Analysis.
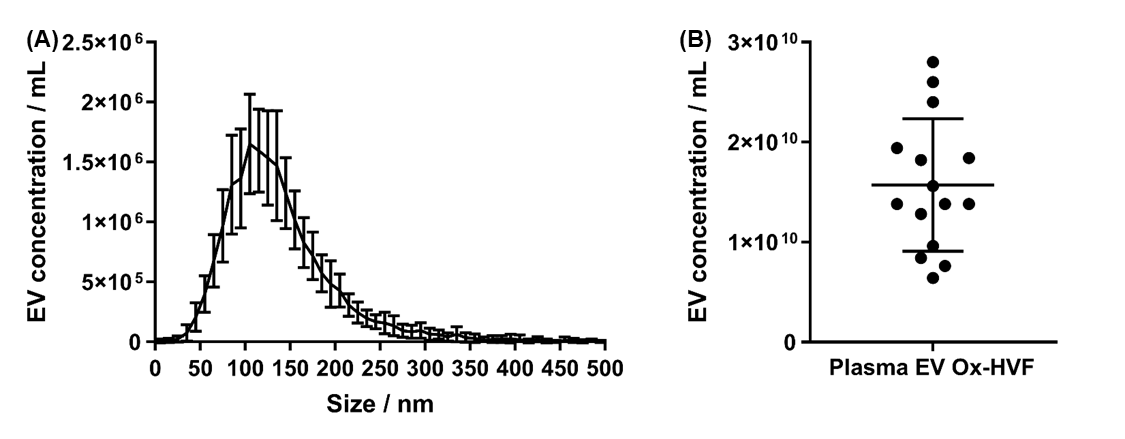


**-ONLINE REFERENCES-**

1. Iepsen EW, Lundgren J, Dirksen C et al. Treatment with a GLP-1 receptor agonist diminishes the decrease in free plasma leptin during maintenance of weight loss. Int J Obes (Lond) 2015;39:834-41.

2. Engelbrechtsen L, Lundgren J, Wewer Albrechtsen NJ et al. Treatment with liraglutide may improve markers of CVD reflected by reduced levels of apoB. Obes Sci Pract 2017;3:425-433.

3. Naz S, Gallart-Ayala H, Reinke SN et al. Development of a Liquid Chromatography-High Resolution Mass Spectrometry Metabolomics Method with High Specificity for Metabolite Identification Using All Ion Fragmentation Acquisition. Anal Chem 2017;89:7933-7942.

4. Akoumianakis I, Sanna F, Margaritis M et al. Adipose tissue-derived WNT5A regulates vascular redox signaling in obesity via USP17/RAC1-mediated activation of NADPH oxidases. Sci Transl Med 2019;11.

5. Pfaffl MW. A new mathematical model for relative quantification in real-time RT-PCR. Nucleic Acids Res 2001;29:e45.

6. Antonopoulos AS, Margaritis M, Coutinho P et al. Adiponectin as a link between type 2 diabetes and vascular NADPH oxidase activity in the human arterial wall: the regulatory role of perivascular adipose tissue. Diabetes 2015;64:2207-19.

7. Margaritis M, Antonopoulos AS, Digby J et al. Interactions between vascular wall and perivascular adipose tissue reveal novel roles for adiponectin in the regulation of endothelial nitric oxide synthase function in human vessels. Circulation 2013;127:2209-21.

8. Thery C, Witwer KW, Aikawa E et al. Minimal information for studies of extracellular vesicles 2018 (MISEV2018): a position statement of the International Society for Extracellular Vesicles and update of the MISEV2014 guidelines. J Extracell Vesicles 2018;7:1535750.

9. Akbar N, Digby JE, Cahill TJ et al. Endothelium-derived extracellular vesicles promote splenic monocyte mobilization in myocardial infarction. JCI Insight 2017;2.

10. Snijder MB, Visser M, Dekker JM et al. Low subcutaneous thigh fat is a risk factor for unfavourable glucose and lipid levels, independently of high abdominal fat. The Health ABC Study. Diabetologia 2005;48:301-8.

11. Teslovich TM, Musunuru K, Smith AV et al. Biological, clinical and population relevance of 95 loci for blood lipids. Nature 2010;466:707-13.

12. Oikonomou EK, Marwan M, Desai MY et al. Non-invasive detection of coronary inflammation using computed tomography and prediction of residual cardiovascular risk (the CRISP CT study): a post-hoc analysis of prospective outcome data. Lancet 2018;392:929-939.
